# Annotation of genomics data using bidirectional hidden Markov models unveils variations in Pol II transcription cycle

**DOI:** 10.15252/msb.20145654

**Published:** 2014-12-20

**Authors:** Benedikt Zacher, Michael Lidschreiber, Patrick Cramer, Julien Gagneur, Achim Tresch

**Affiliations:** 1 Gene Center and Department of Biochemistry, Center for Integrated Protein Science CIPSM, Ludwig-Maximilians-Universität MünchenMunich, Germany; 2 Institute for Genetics, University of CologneCologne, Germany; 3 Department of Molecular Biology, Max Planck Institute for Biophysical ChemistryGöttingen, Germany; 4 Max Planck Institute for Plant Breeding ResearchCologne, Germany

**Keywords:** bidirectional hidden Markov model, chromatin marks, genome annotation, RNA transcription cycle

## Abstract

DNA replication, transcription and repair involve the recruitment of protein complexes that change their composition as they progress along the genome in a directed or strand-specific manner. Chromatin immunoprecipitation in conjunction with hidden Markov models (HMMs) has been instrumental in understanding these processes, as they segment the genome into discrete states that can be related to DNA-associated protein complexes. However, current HMM-based approaches are not able to assign forward or reverse direction to states or properly integrate strand-specific (e.g., RNA expression) with non-strand-specific (e.g., ChIP) data, which is indispensable to accurately characterize directed processes. To overcome these limitations, we introduce bidirectional HMMs which infer directed genomic states from occupancy profiles *de novo*. Application to RNA polymerase II-associated factors in yeast and chromatin modifications in human T cells recovers the majority of transcribed loci, reveals gene-specific variations in the yeast transcription cycle and indicates the existence of directed chromatin state patterns at transcribed, but not at repressed, regions in the human genome. In yeast, we identify 32 new transcribed loci, a regulated initiation–elongation transition, the absence of elongation factors Ctk1 and Paf1 from a class of genes, a distinct transcription mechanism for highly expressed genes and novel DNA sequence motifs associated with transcription termination. We anticipate bidirectional HMMs to significantly improve the analyses of genome-associated directed processes.

## Introduction

An important question in molecular biology is how the occupancy of a genomic position with protein factors relates to the composition of genome-associated protein complexes at this position. This question is of high relevance to fundamental genome-associated processes such as DNA replication, transcription and repair because these generally involve the formation of functional multi-protein complexes that undergo transitions in their protein composition along the genome. For example, during transcription, RNA polymerase (Pol) II progresses through the initiation, elongation and termination phases, which are characterized by the presence of distinct Pol II-associated proteins and various post-translational modifications of Pol II and histones. Analysis of genomewide occupancy maps of Pol II-associated factors obtained by chromatin immunoprecipitation (ChIP) in yeast indicates the presence of distinct protein complexes for the initiation, elongation and termination of transcription, which are formed during a universally conserved mRNA transcription cycle (Venters & Pugh, 2009; Mayer *et al*, 2010; Bataille *et al*, 2012). These conclusions were deduced from metagene analysis, that is, the averaging of occupancy profiles over a pre-selected set of representative genes. In the present work, we check this hypothesis on the single-gene level.

To systematically investigate occupancy profiles in an unbiased, position-specific manner, hidden Markov models (HMMs) (Rabiner, 1989) were used to describe longitudinal observations as a sequence of discrete states (here: genomic states, which model the genome-associated complexes). HMMs have been used to infer chromatin states and annotate enhancers, promoters and transcribed and quiescent regions in the genome of human (Day *et al*, 2007; Thurman *et al*, 2007; Ernst and Kellis 2010; Ernst *et al*, 2011; Ernst & Kellis, 2012; Hoffman *et al*, 2012 2013) and fly (Filion *et al*, 2010; modENCODE Consortium, 2010). For instance, Ernst and Kellis (2010) infer promoter and transcribed chromatin states in human T cells, which occur in a typical order upstream and downstream of annotated transcription start sites (TSSs). However, these state-of-the-art HMM approaches infer genomic states in a non-strand-specific (or undirected) manner. For example, they cannot decide whether a bona fide ‘TSS upstream’ state generally precedes or follows a bona fide ‘TSS downstream’ state. Directionality information needs to be included in a post-processing step. Moreover, these models lack a sound way to integrate strand-specific (e.g., expression) with non-strand-specific (e.g., ChIP) data, which is indispensable to appropriately characterize strand-specific genomic processes.

To address these issues, we develop the theory of bidirectional hidden Markov models (bdHMMs), a novel probabilistic model that annotates directed states from non-strand-specific data (such as ChIP), and optionally strand-specific data (such as RNA expression). We introduce the concept of ‘directed genomic states’, which encode directionality information and thus provide a more realistic model of the underlying genome-associated complexes and their transitions. We present a very efficient algorithm for the learning of the bdHMM, available as an R/Bioconductor package STAN (http://www.bioconductor.org/packages/devel/bioc/html/STAN.html). The broad applicability of our method is demonstrated on two entirely different datasets, namely on a tiling array transcription factor dataset in yeast and a deep-sequencing histone dataset in human. We show that bdHMM produces more accurate genome annotations than standard HMM. Our bdHMM analysis of previously defined chromatin states in human T cells (Ernst & Kellis, 2010) *de novo* identifies directed chromatin state patterns and provides an improved annotation of the human ‘histone code’. Application of the bdHHM method to a set of 22 genomic profiles in the *S. cerevisiae* finds new transcription units and DNA sequence motifs and unveils so far unknown variations in the Pol II transcription cycle. The yeast and human datasets, their state annotation and bdHMMs, which generated them, are available from the website http://www.treschgroup.de/STAN.html. Using essentially the same set of parameters, the bdHMM is as easy to learn as standard HMM while extracting more information. We therefore anticipate bdHMM to replace standard HMM in a wide range of genomic analyses.

## Results

### Annotation of directed genomic states using bdHMMs

**Figure 1:**
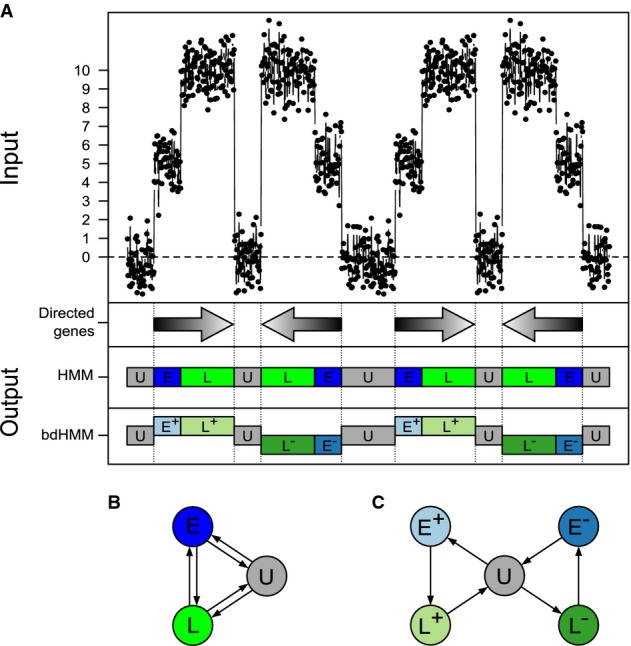
Principle of bidirectional HMM (bdHMM). Simulated occupancy signal (1^st^ track from the top) for a putative factor with a low level (centered at 0) in untranscribed regions (state U), an intermediate level in 5' part of genes (state E), and a high level in 3' part of genes (state L). Arrows (2^nd^ track) depict boundaries and orientation of transcription. Unlike standard HMM (3^rd^ track), bdHMM (4^th^ track) infers strands (+ or −) to expressed states (E, L).HMM transition graph. Because orientation of transcription is not modeled by standard HMM, the spurious reverse transitions (*E* ⇒ *U*,*L* ⇒ *E* and *U* ⇒ *L*) are as likely as the correctly oriented transitions (*U* ⇒ *E*,*E* ⇒ *L* and *L* ⇒ *U*).bdHMM transition graph. In contrast to HMM, bdHMM, which has explicit strand-specific expressed states (*E*^+^/*E*^−^ and *L*^+^/*L*^−^), allows inferring only the correctly oriented transitions.

Standard and bidirectional HMMs are best understood with the help of a simulated dataset. A precise definition of the HMM and a bdHMM is given in the Materials and Methods. The example in Fig1 considers a part of the genome where transcription occurs as a sequence of three different genomic segments. The transcribed regions split into segments of early (E) and late (L) transcription activity, and they are flanked by untranscribed (U) segments. The order of the three segments U, E and L along the genome depends on the orientation of the respective gene (Fig1A, gray arrows). ChIP measurements *o*_0_,*o*_1_,…,*o*_*T*_ for a single protein at genomic positions *t* = 0,1,…,*T* were simulated with low (U), medium (E) and high (L) average occupancy in the different segments. Note that these ChIP signals do not contain strand-specific information. An HMM defines a probability distribution on a sequence of observations *o*_0_,…,*o*_*T*_. It assumes that each observation *o*_*t*_ is *emitted* by a corresponding (unobserved) state variable 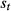, which can assume values from a finite set of hidden states. The value of 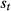 determines the probability of observing *o*_*t*_, 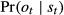. The hidden variables form a first-order Markov chain, which means that the probability for observing 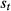 depends only on *s*_*t*−1_, the transition probability 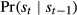. After the learning of these probabilities, the HMM outputs the so-called Viterbi path, which is the most likely state sequence 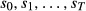 that generated the observations. In our example, the Viterbi path provides a genome annotation.

A standard HMM with 3 hidden states can distinguish the three protein occupancy levels; the three states correspond to the three genomic segments (Fig1B) and are therefore also called U, E and L. However, the transition probabilities in the standard HMM are symmetric because the number of observed transitions between successive segments, say E to L, in the forward direction equals the number of transitions in the reverse direction, L to E. Hence, standard HMMs are neither able to capture the strand specificity of transcription (i.e., the two different directions of transcription along the genome) nor do they infer biologically meaningful transitions along the genome as they occur during transcription.

In order to infer directed transitions and directed genomic states, bdHMMs have ‘twin states’, one for each strand and genomic state. For instance, the early state E is split up into the twin states *E*^+^ and *E*^−^. Twin states are coupled by two symmetry conditions. First, twin states are required to have identical emission probabilities, that is, in our example, 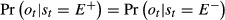, where *o*_*t*_ is the observed data and 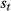 is the hidden (transcription) state at position *t*. Second, twin states satisfy transition symmetry, a novel generalization of reversible Markov chains (see Materials and Methods for details), which requires that state transitions are invariant under reversal of time and direction, that is, 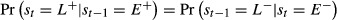. In our example, this results in the bdHMM transition probabilities 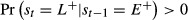 and 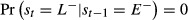, as opposed to 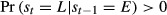 and 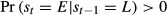 in the HMM (Fig1B and C). These two conditions enable the recovery of the direction of genomic states (Fig1A). Although the formal number of states doubles, the effective number of parameters does not increase due to the bdHMM constraints.

Parameters are inferred using a constrained Baum–Welch algorithm, the validity of which was assessed by simulations showing that model parameters and states were recovered with high accuracy, even when only few training data were used (Materials and Methods, Supplementary Figs S8 and S9). The bdHMM is implemented in the R package STAN (STrand-specific ANnotation of genomic data), which is freely available on Bioconductor (http://www.bioconductor.org/packages/devel/bioc/html/STAN.html).

### Genomic state annotation results in a global, strand-specific transcription map

**Figure 2:**
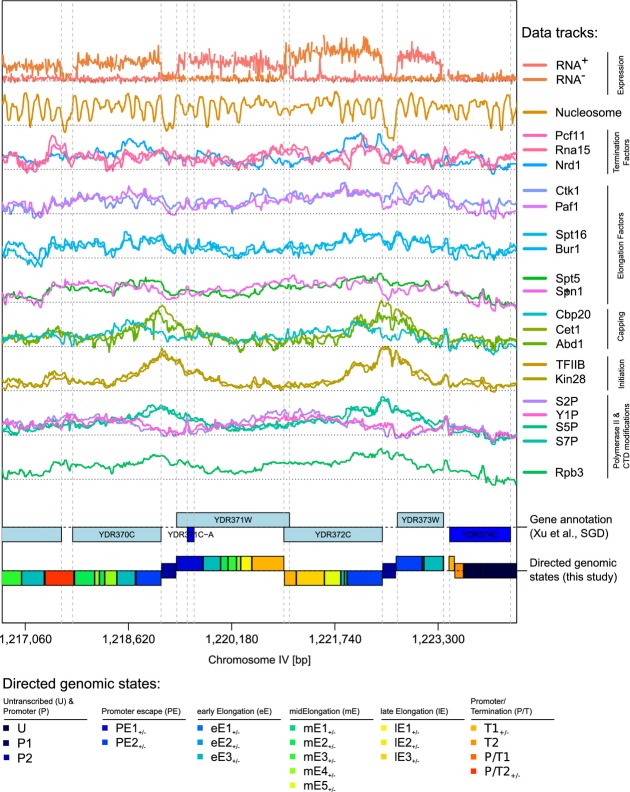
*De novo* annotation of directed genomic states from genomewide transcription data in yeast using bdHMM Inputs for the bdHMM are the following, from top to bottom: strand-specific wild-type RNA levels, occupancy maps of nucleosomes, 3 termination factors, 6 elongation factors, 3 capping factors, 2 initiation factors, 4 CTD modifcations and 1 core Pol II member (Rpb3). Inferred directed genomic states are shown as colored boxes in the lowest track (see color legend beneath) where expressed states on the + (respectively −) strand are positioned above (respectively under) the axis, and not expressed states are centered on the axis. Previous transcriptome annotation is shown in the 2^nd^ track from the bottom.

We applied the bdHMM to ChIP data in *S. cerevesiae*, where high-resolution datasets for dozens of proteins of the transcription machinery are available. We compiled genomewide ChIP-chip experiments for transcription initiation factors (TFIIB, Kin28), elongation factors (Spt5, Spn1, Bur1, Spt16, Ctk1, Paf1), termination factors (Pcf11, Rna15, Nrd1), Pol II and various modifications of its C-terminal domain (CTD) (Tyr1P, Ser2P, Ser5P, Ser7P) and nucleosomes (Lee *et al*, 2007; Mayer *et al* 2010, 2012; Lidschreiber *et al*, 2013). The dataset was complemented by strand-specific mRNA expression data (Xu *et al*, 2009) (Fig2).

The number of bdHMM states needed to be specified in advance. Bearing in mind that our states should distinguish biologically different genomic states, classical model selection criteria (BIC, AIC, MDL) are not useful. Those criteria balance the number of parameters/states against the precision of the data fit. Since our data are very rich, they suggest a very high number of states, which cannot be interpreted. This issue has been reported repeatedly in association with HMMs (Ernst and Kellis, 2010; Hoffman *et al*, 2013, 2012) for integrative analysis of ChIP data. We tried several state numbers (data not shown) and found that 20 states yield an appropriate trade-off between model complexity and biological interpretability (see Materials and Methods). Simulations from the inferred bdHMM recovered model parameters with high accuracy and further confirmed the validity and stability of our model (Supplementary Fig S9).

The genomewide state annotation was derived as the most likely state path (Viterbi decoding, Fig2), which partitioned the 12-Mb yeast genome into 48,507 directed and 10,760 undirected state segments with distinct bdHMM states. This yields a strand-specific partitioning of the yeast genome into segments of directed genomic states. Alternative to Viterbi decoding, posterior decoding or mixed approaches (Posterior-Viterbi decoding, Fariselli *et al*, 2005) could be used. Generally, Viterbi decoding is less subject to state flipping compared to posterior decoding. However, we did not see relevant differences between both approaches in this application (97% of genomic positions are annotated with the same state when comparing Viterbi and posterior decoded state paths).

### bdHMM state annotation recovers annotated genomic features with high accuracy

In principle, the strand-specific expression of this dataset could also be used with standard HMMs to learn directed states. However, fitting a standard HMM did not recognize directed genomic states. In particular—since the HMM is learned without symmetry constraints for twin states—there is no obvious pairing between the forward (+) and the reverse (−) states, demonstrating the need for bdHMM (Materials and Methods, Supplementary Fig S1).

**Figure 3:**
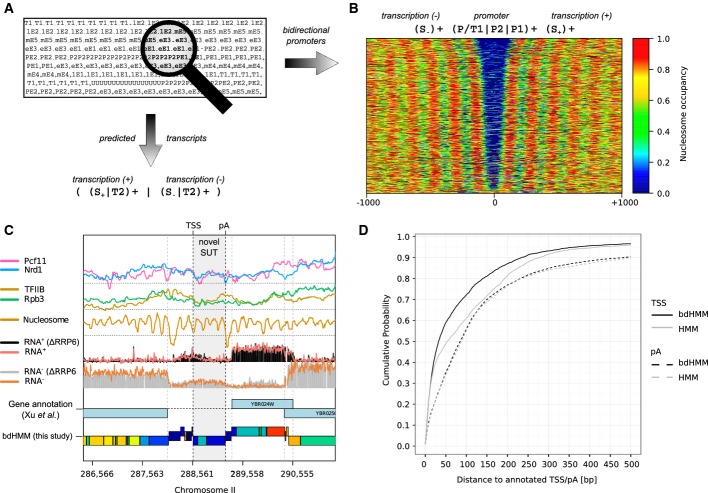
Genomic state annotation predicts bidirectional promoters and (novel) transcripts. The genomic state annotation (viterbi path) was searched with regular expressions (RegEx) defining bidirectional promoters (right) and transcripts (bottom).Nucleosome binding patterns centered at 1,076 identified bidirectional promoters found with the RegEx. Each line in the heatmap corresponds to one pair of transcripts. Binding signal is color-coded (right).A novel SUT (stable unannotated transcript, a stable non-coding RNA, gray area) is identified on the − strand by the bdHMM. The locus shows detectable expression but was too low for the criteria used by Xu *et al* (2009).Estimated cumulative probability of TSS and pA site predictions shows higher accuracy of bdHMM in recovering TSSs. pA site prediction has similar accuracy for both models.

In order to re-annotate transcription throughout the yeast genome and compare the performance of bdHMM and HMM, we applied a regular expression (RegEx) approach (Fig3A), to predict transcribed units as continuous stretches of directed transcribed states with a minimal length of 80 bp on both strands from the bdHMM and HMM annotation. Matching predicted transcript boundaries to previously published ones (Xu *et al*, 2009), 4,186 (82%) of all annotated protein-coding transcripts were recovered from the bdHMM predictions, 11% more than the HMM predicts using the same criteria (3,639 transcripts) (best reciprocal hits, Materials and Methods). Moreover, the predicted transcription start sites (TSS) were consistently closer to the annotated ones (Fig3D). In particular, 60% of the predicted TSSs by the bdHMM were within 50 bp, whereas the best 60% of the HMM TSS predictions were within 100 bp of the published ones. Accuracy of pA site prediction was lower, but comparable between bdHMM and HMM, where approximately 60% of the predicted pA sites were within 100 bp of the annotated ones for both methods. Moreover, 32 novel transcripts were predicted from the bdHMM annotation (four overlapping a coding region, 28 non-coding, Fig3C, Materials and Methods), which is of particular significance because the *S. cerevisiae* transcriptome has been thoroughly studied and annotated.

As another illustration of genomic features that can be extracted from a bdHMM annotation, we searched for bidirectional promoters using a RegEx consisting of a promoter state flanked by an upstream transcript on the Crick strand and a downstream transcript on the Watson strand (Fig3A and B). We detected 1,076 bidirectional promoters in yeast, which agrees well with a previous estimate of 1,049 bidirectional promoters (Xu *et al*, 2009). Altogether, these results demonstrate the high accuracy of the bdHMM for genome annotation and its advance over the standard HMM.

### Transcription cycle phases have a substructure

**Figure 4:**
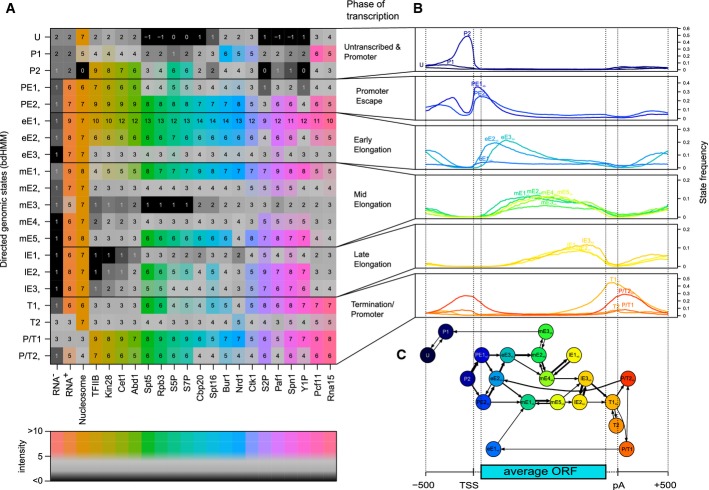
Roles of directed genomic states in the transcription cycle. Mean ChIP enrichment of factors (horizontal axis) indicates the composition of the transcription machinery in each state (vertical axis). Factors were ordered by hierarchical clustering, and states were ordered by position of their most frequent occurrence along the average gene.Each state was assigned to a phase in the transcription cycle by investigating the frequency (*y*-axis) of each state at an average transcript. This spatial state distribution was calculated from the genomic sate sequences (viterbi paths) of 4,362 genes.The flux diagram shows probabilities of state transitions calculated from the viterbi paths. Branches mark alternative successions of states at individual genes and thus reveal extensive variation in the transcription cycle as it is modeled by the genomic states. Each node (state) is positioned according to the most frequent position on a metagene. The diagram contains at least one incoming and one outgoing transition for each state as well as transitions observed with a frequency > 0.01 on the metagene.

To understand how the 20 bdHMM states relate to phases of the transcription cycle, we analyzed their average frequencies along annotated, transcribed genes (Fig4B, Materials and Methods). The states showing a single frequency peak (18 out of 20 states) were grouped into six transcription phases, according to the location of their peak on the average gene: promoter (P, 2 states), promoter escape (PE, 2 states), early elongation (eE, 3 states), mid-elongation (mE, 5 states), late elongation (lE, 3 states) and termination (T, 2 states). Two states showed two peaks in frequency, in each case with one peak upstream of the transcription start site and one peak around the polyadenylation (pA) site. We interpreted these two states as mixed promoter and termination states and labeled them accordingly P/T1 and P/T2 (Fig4A and B). Hence, although overlapping transcription is not explicitly modeled by bdHMMs, this phenomenon could be captured by specific states.

The mean factor occupancy defining a particular state is indicative of the composition of the transcription complex and its activity (Fig4A). Indeed, we found that the enrichment or depletion of protein factors in each state was in accordance with their known roles in transcription (Fig4A). For instance, the initiation factors TFIIB and Kin28 were enriched in promoter and promoter escape states (P2, PE1, PE2) and were depleted in states of other transcription phases (Fig4A and B). States related to the same transcription phase often peaked at successive genomic positions. For instance, the mid-elongation phase comprises successive states mE1–mE5 (Fig4B and C) that were characterized by a gradual decrease in the occupancy of initiation factors, capping-related factors and Nrd1 (Fig4A).

The association of states to phases of transcription is in accordance with state-specific enrichment of DNA sequence motifs (Materials and Methods, Supplementary Information). While promoter state P2 shows enrichment of known promoter-associated motifs, termination state T1 is enriched with known termination signals and mixed state P/T2 contains both promoter- and termination-associated motifs. We also found potentially unknown sequence motifs, which we could not associate to known functions or binders (Supplementary Information). Overall, these results show that unsupervised bdHMM analysis can define meaningful genomic states that reflect phases of transcription at every single gene.

### The transcription cycle shows gene-specific variation

Our bdHMM annotation did not only recapitulate known events during transcription, it also provided unexpected, new insights. For example, the flux diagram (Fig4C, showing the most likely transitions between successive states) indicated variability within the transcription cycle. We found different states at the same position within genes that may reflect alternative functional transcription complexes (promoter: P1, P2, P/T1, or P/T2; promoter escape: PE1 or PE2; Fig4A and B). These alternative states are located within different branches of the flux diagram (Fig4C). A pronounced bifurcation occurs at the transition from P2 to promoter escape, entering either highly productive (PE2) or weak transcription (PE1). These two branches of the transcription cycle converge again during late elongation (lE2, lE3) or termination (T1). Hence, the analysis of state frequency distributions and transition diagrams suggests gene-specific variation of the transcription cycle.

**Figure 5:**
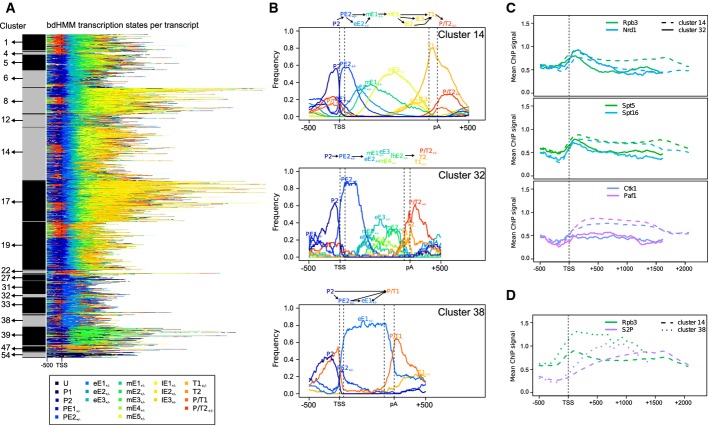
Clustering of state paths reveals gene-specific variations in the transcription cycle. Genomic state sequences of 4,632 genes were clustered into 55 groups (left, only clusters containing at least 20 genes are labeled). Each line corresponds to the state sequence of a single gene. States are colored as shown in the legend.Clusters exhibit distinct state frequency distributions and transition patterns (shown as schematic flux diagrams on top of panels). Cluster 14 shows a transcription cycle closest to the canonical one proposed by Mayer *et al* (2010). Genomic state sequences of clusters 32 and 38 differ from the canonical one, indicating variations in the transcription cycle.Clusters 14 and 32 exhibit distinct recruitment of factors to genes. PolII subunit Rpb3, Nrd1, Spt5 and Spt16 binding is very similar in the beginning of genes, but decreases much more strongly in cluster 32 throughout the transcripts. Ctk1 and Paf1 are depleted at cluster 32, but not at cluster 14 genes.Cluster 14 shows the canonical Pol II (Rpb3) peak in the 5' region of genes, but Pol II reaches a stable, high level downstream of the TSS in cluster 38. This may suggest a lack of the mechanism for Pol II peaking observed in cluster 14. The steep increase of serine 2 phophorylation in cluster 38 might indicate that productive elongation is reached earlier at those genes.

For a systematic investigation of gene-specific variation during the transcription cycle, we clustered genes based on their annotated state path. To that end, the state paths of 4,263 genes were rescaled to a common length and clustered into 55 groups according to their Hamming distance (Fig5A and 5B, Materials and Methods). The obtained gene clusters show distinct patterns of protein occupancies, suggesting mechanistic differences in transcription (Fig5, Supplementary Fig S2 and below). Moreover, the gene clusters differed by gene length, expression level and genomic context (e.g., termination overlaps with neighboring downstream promoters or bidirectionality of promoters). Gene set enrichment analysis showed that clusters also corresponded to distinct functional gene groups (Supplementary Table S1). The functional categories range from housekeeping (e.g., cluster 14, 38), cell cycle (e.g., cluster 17) to stress response (e.g., cluster 39). For instance, the high expression of cluster 38 and 14 is in accordance with their associated functions including ribosome biogenesis, positive regulation of transcription, translation or nucleosome assembly. More strikingly, we found the DNA binding motif of SFP1—a regulator of ribosomal protein and ribosome biogenesis genes—to be enriched in promoter state P/T1 (which is a frequent promoter state of cluster 14 and 38 genes, Supplementary Fig S4). In contrast, stress- and autophagy-related genes in cluster 39 show very low expression and protein binding (Supplementary Fig S2B). Altogether, this suggests that different transcription cycles as they are modeled by the bdHMM correspond to different co-regulated gene sets.

Cluster 14, which contains 694 genes (Fig5B and 5C, Supplementary Fig S2B), shows a transcription cycle most similar to the canonical one proposed previously (Mayer *et al*, 2010). In this cluster, the promoter escape state PE2 was characterized by peak occupancy of the Pol II core subunit Rpb3 between 100 and 200 bp downstream of the TSS, and phosphorylation of the CTD serine 2 residue reaches maximum levels between 600 and 1,000 bp (Fig5D), as observed in previous metagene analysis. The cycle ends with the canonical termination state T1, which is characterized by the presence of elongation factors Spn1, Paf1, Ctk1, Bur1, Spt16 and Spt5 and termination factors Pcf11 and Rna15 (Fig4A).

### Evidence for regulated promoter escape

We next analyzed clusters with variations compared to the canonical transcription cycle. Cluster 32 (43 genes) differs from the canonical cluster 14 in the transition from promoter escape to elongation. State frequency and gene-averaged ChIP signals suggest that transcription is attenuated after promoter escape in cluster 32 (Fig5B and 5C). In this cluster, a strong promoter escape (PE2) is followed by the weak elongation state eE3, which is characterized by low levels of Pol II and elongation factors (Fig5C). Moreover, elongation factors Ctk1 and Paf1 appear to be absent from those genes (Fig5C, Supplementary Fig S2C). In contrast, cluster 14 exhibits similarly strong promoter escape yet transitions into the highly productive elongation states eE2 and mE1, which are characterized by high occupancies of all measured elongation factors (Fig4B and 4C). This comparison supports the existence of a regulatory checkpoint for transcription elongation after promoter escape. This is likely related to transcription attenuation with the help of the early termination factor Nrd1 (Schulz *et al*, 2013) (Fig5C, Supplementary Fig S3). The individual occupancy profiles (Fig5C, Supplementary Fig S2C) indicate that this checkpoint separates the binding events of Spt5, Spn1, Bur1 and Spt16 from the binding of Ctk1 and Paf1. Thus, it appears that attenuated genes recruit early elongation factors including Spt5 and Spt16, but not the later factors Paf1 and Ctk1.

### Evidence for distinct transcription mechanisms for highly expressed genes

Cluster 38 differs strikingly from the canonical transcription cycle during early elongation and termination (Fig5B, Supplementary Fig S2D, 147 genes enriched for genes involved in translation, Supplementary Table S1, Materials and Methods). Cluster 38 is characterized by the high occupancy promoter state P/T1 (Fig 4A) and by the early elongation state eE1 (for 58% of all cluster 38 genes, and in turn, 48% of genes with eE1 state are in cluster 38). During early elongation, serine 2 phosphorylation levels increase more steeply than in cluster 14, indicating that productive elongation is reached earlier at those genes (Fig 5D). Moreover, Pol II does not exhibit the typical occupancy peak 150 bp downstream of the TSS but immediately reaches a stable high level (Fig 5D). This profile could be the consequence of a lower drop-off rate at this position (Mayer *et al*, 2010), a more constant elongation rate along the gene, or a high and uniform coverage by elongating polymerases. Specifically to cluster 38, a sharp decrease of the occupancy of essentially all factors is observed well positioned at the stop codon. The data indicate that most factors (Cbp20, Nrd1, Ctk1, Paf1, S5P, S7P, Spt16 and Bur1) are then released, as their occupancy remains low after the stop codon. Moreover, the Pol II subunit Rpb3, the serine 2 phosphorylation and the elongation factors Spt5 and Spn1 recover their occupancy levels at the pA site, suggesting a higher elongation rate for Pol II and that these factors stay bound to the transcription machinery within the 3' UTR. This indicates that the previously reported early release of elongation factors for ribosomal genes (Mayer *et al*, 2010) is sharply positioned at the stop codon and also involves release of the cap-binding protein Cbp20, the early termination factor Nrd1 and dephosphorylation of the CTD residues Ser5 and Ser7. Taken together, cluster 38 suggests that highly expressed genes exhibit distinct transcription mechanisms, characterized by efficient factor recruitments during early elongation and specific processes of factor release around the stop codon.

### Not all termination regions are depleted of nucleosomes

Nucleosome depletion has been reported at the 3' end of genes (Mavrich *et al*, 2008). However, cluster 19, whose 634 genes terminate in state T1, does not show nucleosome depletion in this region. In contrast, nucleosome depletion is a hallmark of all our promoter states. We therefore hypothesized that the termination of genes in clusters other than cluster 19 overlaps with promoters of downstream genes. Genes in clusters 1, 5, 6, 12, 32, 33 and 38 showed nucleosome-depleted termination states P/T1 and P/T2. Their termination regions indeed overlap with a downstream promoter, as indicated by TFIIB enrichment downstream of their pA site (Supplementary Fig S2). This supports previous reports that nucleosome depletion is not an intrinsic mark of transcription termination (Fan *et al*, 2010). Thus, bdHMM analysis of the genomic context of transcription allows distinguishing canonical binding patterns from spurious ones caused by spillover effects from neighboring genes.

### Comparison to standard HMM on chromatin states of human T cells

**Figure 6:**
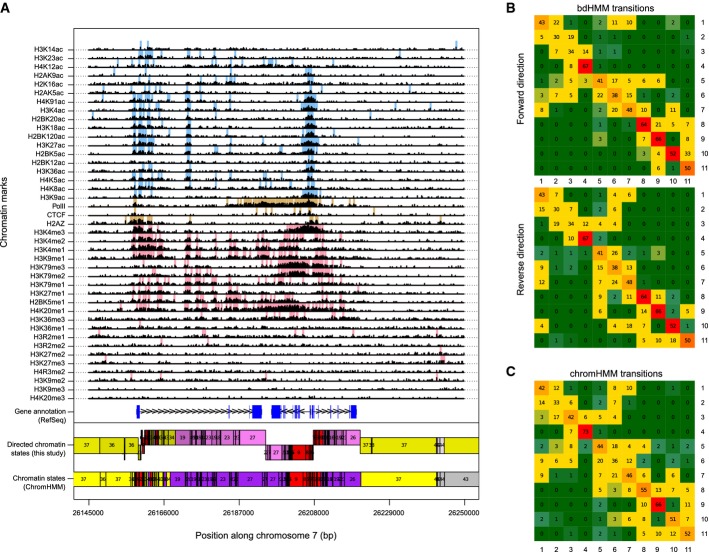
Application of bdHMM to chromatin modifications in human T cells identifies direction of chromatin states. Example of chromatin state annotation of ChromHMM and bdHMM (bottom tracks) with RefSeq gene annotation and input signal. State direction matches gene orientation of annotated convergent genes and divergent genes. The log-transformed signal (Ernst and Kellis, 2010) of all 41 data tracks is shown in black on top. Binarized input signal is shown for 18 acetylation marks in blue, 20 methylation marks in red and CTCF/PolII/H2A.Z in brown.bdHMM transitions between promoter-associated states 1–11 are shown for forward and reverse states. The asymmetric, transposed structure of these two submatrices (i.e., transition probabilities favor one direction for pairs *a*_*ij*_ and *a*_*ji*_) uncouple the two reading directions.The symmetric ChromHMM transition matrix hides the underlying directed flow of chromatin states.

We evaluated the performance of bdHMM on sequencing data and large genomes, by applying bdHMM to a dataset of 41 chromatin marks in human T cells (Ernst & Kellis, 2010). The chromatin mark data had been binarized into presence/absence of each mark at a resolution of 200 bp bins and analyzed with a standard HMM approach (ChromHMM) (Ernst & Kellis, 2012). To handle the binarized chromatin marks data defined by Ernst and Kellis (2010), we extended bdHMM and included binary (Bernoulli) emission distributions. We fixed the emission distributions during bdHMM learning, allowing a direct comparison of bdHMM states to HMM states. Moreover, this ensured that differences in the result are only due to differences in the modeling of state transitions. We developed a directionality score (Materials and Methods) to decide that in the bdHMM, 35 out of a total of 51 ChromHMM states are modeled as directed state pairs and 16 ChromHMM states are modeled as undirected states. Consistently, we identified directed chromatin states around transcribed, but not at repressed or repetitive regions (Supplementary Fig S5). Up to state directionality, 83% of state annotations agreed between the two methods (Materials and Methods). Comparison of the ChromHMM with the bdHMM transitions revealed that in ChromHMM, transition probabilities between two states are similar in both directions (Fig6C), whereas the bdHMM can resolve the true order of chromatin states (Fig6A and 6B, Supplementary Fig S6). For example, transitions from state 6 into states 2 and 3 are high for the forward direction, but low for the reverse drection. In contrast, transitions from states 2 and 3 into state 6 are high in reverse, but low in forward, direction (Fig6B). However, all of these transitions are high in the symmetric ChromHMM model (Fig6C), demonstrating that bdHMM adds previously unexploited and valuable information to HMM-based analyses by uncoupling the underlying state directionality of genomic processes. Analysis of promoter and transcribed state frequencies at the TSS showed that state annotations matched the reading (sense) direction of the transcribed loci with up to 85% (Supplementary Fig S7). Promoter states showed pronounced peaks in sense direction at the TSS, which are further downstream followed by high frequencies of (sense) 5' proximal transcribed states. We conclude that bdHMM significantly improves the annotation of the human epigenome, because it correctly recovers the flow of chromatin states as they occur during transcription.

## Discussion

We introduced bidirectional hidden Markov models (bdHMMs), a method for *de novo* and unbiased inference of directed genomic states from genomewide profiling data. In contrast to previously described HMM-based approaches, bdHMM explicitly models directed genomic processes. It allows for the integration of strand-specific experimental data such as RNA expression profiles together with non-strand-specific data, such as ChIP occupancy data, and outperforms standard HMM in genomic feature annotation. The open-source package STAN provides a fast, multiprocessing implementation that can process the human chromatin dataset in < 1 day.

Application of bdHMM analysis significantly improved insights into previously defined combinatorial chromatin marks (Ernst & Kellis, 2010), indicating the presence of directed chromatin state patterns around the transcribed, but not the repressed, portion of the human genome. Our analysis of gene transcription in the budding yeast enabled us to automatically recover the majority of known and even new Pol II transcription units at a higher accuracy than standard HMM. We could assign different directed genomic states that are characterized by the presence of different transcription factors and Pol II CTD modification marks.

The most significant advance of bdHMM analysis over previous methods is its potential to *de novo* identify characteristic sequences (patterns) of directed states on the genome. These patterns identify gene-specific variation in transcription—or other directed processes—that were previously hidden by metagene analysis of experimental data. Metagene analysis derives only average profiles for groups of genes defined beforehand and is thus biased toward annotated genes. In contrast, bdHMM allows investigating variations in the sequence of genomic states associated with transcription. This is done by first identifying distinct genomic states *de novo* and then clustering genes based on the succession of these genomic states. This analysis was consistent with a general transcription cycle and uniform transitions of a core Pol II transcription complex that occurs at all genes (Venters & Pugh, 2009; Mayer *et al*, 2010; Bataille *et al*, 2012). On the other hand, it also indicated gene-specific variations to the general transcription cycle, because the resulting clusters differed markedly in the sequence of their genomic states. First, a few dozen genes that apparently show Nrd1-mediated transcription attenuation are shown here to lack elongation factors Ctk1 and Paf1, suggesting that transcription attenuation occurs before Ctk1 and Paf1 are recruited. Second, we provide evidence for a distinct mechanism for highly expressed genes leading to the immediate recruitment of a full complement of Pol II-associated factors downstream of the transcription start site. Third, we found that nucleosome depletion is not a necessary feature of transcription termination.

Thus, we foresee bdHMMs to be instrumental for studying gene transcription and other directed genomic processes, such as DNA replication, recombination or DNA repair.

## Materials and Methods

### Experimental data and preprocessing

The experimental yeast dataset was compiled from public data (Lee *et al*, 2007; Xu *et al*, 2009; Mayer *et al*, 2010, 2012; Lidschreiber *et al*, 2013). All measurements were done using the high-density custom-made Affymetrix tiling array (PN 520055) which tiles each strand of genomic DNA in yeast at a resolution of 8 bp. ChIP experiments were normalized using the R/Bioconductor (Ihaka & Gentleman, 1996; Gentleman *et al*, 2004) package Starr (Zacher *et al*, 2010) as previously described (Zacher *et al*, 2011). Expression data were normalized using the tilingArray package (Huber *et al*, 2006).

The human chromatin modification dataset was downloaded from the supplemental website of Ernst and Kellis (2010), where they provided the preprocessed sequencing and binary data.

### The bidirectional hidden Markov model

Bidirectional hidden Markov models belong to the class of hidden Markov models (HMMs). It is therefore beneficial to introduce HMMs first, along with some notation.

*Definition*. A hidden Markov model (HMM) is a tuple 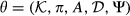 such that

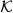
 is a finite set, the elements of which are called *states*.
2. The *initial state distribution* 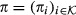 is a probability (row) vector, that is, 0 ≤ *π*_*i*_ ≤ 1, 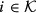, and 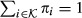.
3. The *transition matrix* 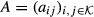 is a 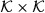 (row) stochastic matrix, that is, each row of *A* is a probability vector.
4. The *emission distributions* 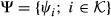 form a set of probability distributions on a space 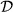, the *space of observations*.

An HMM defines a probability distribution on a sequence of observations 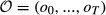 of length *T* + 1. It assumes that each observation *o*_*t*_ is *emitted* by a corresponding hidden (unobserved) state variable 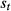 which can assume values in 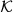. The value of 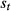 determines the probability of observing *o*_*t*_ by 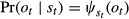. The hidden variables are assumed to form a homogenous Markov chain 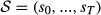, with (time independent) transition probabilities 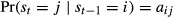, 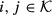, *t*=1,...,*T*, and with initial state distribution 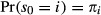, 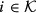. The (full) likelihood of an HMM is

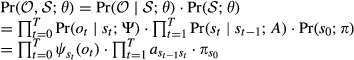
1

A bdHMM is an HMM which satisfies three additional conditions. The first two conditions deal with the structure of the underlying hidden Markov chain, and the last condition considers the nature of observations. As will be shown in the subsequent paragraph on the semantic of bdHMMs, these conditions are by no means *ad hoc*.

*Definition*. A bidirectional hidden Markov model (bdHMM) is a tuple 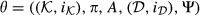 such that 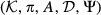 is an HMM, 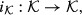 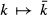 and 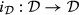, 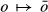 are involutions (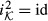, 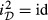), and the following symmetry conditions hold:

1. *Generalized detailed balance*: The transition matrix *A* and the initial state distribution *π* satisfy 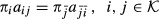
2
  *Initiation symmetry*: The initial state distribution *π* satisfies 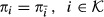
3
*Observation symmetry*: Ψ satisfies

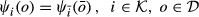
4

#### The semantic of bdHMMs

Why did we choose (2), (3) and (4) as the defining properties of a bdHMM? In order to motivate our choice, let 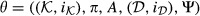 be a bdHMM. By initiation symmetry and generalized detailed balance,

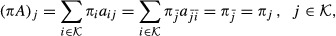
which proves *πA* = *π*. In other words, the initial state distribution *π* of a bdHMM is always a stationary state distribution of *A*. It might be surprising that the initital state distribution has to match the steady state probabilities. This is, however, an uncritical constraint in practical applications, for two reasons. First, low-complexity regions (unassembled regions, repeat regions, telomeres, centromeres, etc.) lead to frequent large stretches of missing values. Hence, the model is not run on complete chromosomes, but on the remaining regions with complete data. Therefore, taking the steady state probability as initial probability is a reasonable modeling assumption. Second, these regions are typically long enough so that the initial state distribution has minimal influence on genomic state annotation.

Moreover, generalized detailed balance and initiaion symmetry together imply that the relation

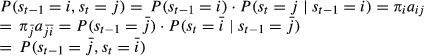
6holds for all states 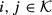 and all positions *t* = 1,…,*T*. This is a most natural condition as it says that at any position of the state sequence, the probability of consecutively observing states *i* and *j* equals that of observing the respective conjugate states in reversed order. Vice versa, (5) obviously implies generalized detailed balance. Under the mild assumption that 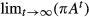) always exists (this is the case, e.g., if the matrix *A* is ergodic, see Seneta 2006), it can be shown that (5) also implies initiation symmetry: By induction, using

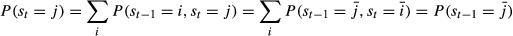
7it follows that 
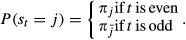
Therefore,

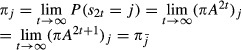
8which is exactly condition (3). Hence the natural condition (5) is essentially equivalent to (2) and (3). The reason for using the latter two conditions for the definition of a bdHMM is that they are simple relations in terms of the model parameters *π* and *A*.

Bidirectional HMMs model directional processes in a sequence of observations. It is reasonable to expect that an observation contains information about the directionality of the underlying process that generated it. The involution 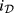 is meant to map an observation 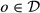 to its so-called conjugate observation 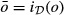, which denotes the corresponding observation that one would make if the observation sequence were viewed from the opposite direction. For example, in the case of genomic measurements, 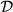 is modeled as 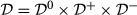, the Cartesian product of a space 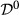 of non-strand-specific observations (e.g., ChIP measurements of protein binding), a space 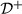 of forward strand-specific observations (like RNA transcription originating from the forward strand), and a corresponding set 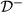 of reverse strand-specific observations. The forward and reverse strand-specific observations are paired in the sense that 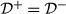. The involution 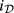 acts as the identity on 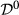, and it swaps the strand-specific observations, 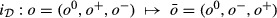. In hidden Markov models, observations will be emitted from hidden states that may indicate typical processes occurring in forward or in reverse direction, or undirectional processes. The involution 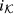 splits the states 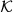 of the HMM into undirected states (denoted by 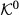
—the fixed points 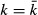 of *i*_*k*_—and directed states which occur in pairs 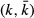, 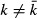 of ‘conjugate’ or ‘twin’ states. One member of such a pair is deemed to be involved in forward and the other in reverse directional processes (note that at this point we do not specify which of the two does what). The forward states are denoted by 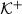 and the reverse states by 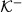. The observation symmetry condition (4) merely ensures that conjugate directed states encode essentially the same probability distribution, up to reversal of the observations.

Note that if 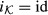 is the identity map, condition (3) is void, and condition (2) reduces to the common detailed balance relation for reversible HMMs. If additionally the involution 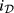 is the identity map, condition (5) is also void. Thus, a bdHMM 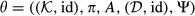 is nothing but a reversible HMM, that is, an HMM which additionally satisfies the (standard) detailed balance relation *π*_*i*_*a*_*ij*_ = *π*_*j*_*a*_*ji*_, 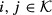. It follows that our algorithms for bdHMM learning will immediately apply to reversible HMMs.

Given an observation sequence 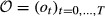, let 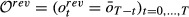 denote the ‘reversed’ observation sequence obtained by taking conjugates of all observations and reversing their order. Similarly, given a hidden state sequence 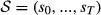, let 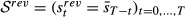 denote the ‘reversed’ hidden state sequence. Verify that

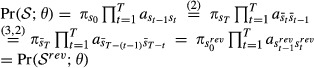
9

Moreover,

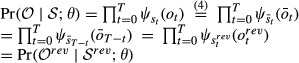
10

Equations and imply 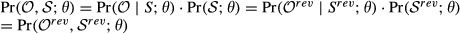
11and 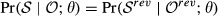
12

Finally, a bdHMM is reversible in the generalized sense, 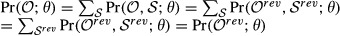
13

The second-last equality in (12) holds because if 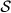 runs over all possible state sequences, then so does 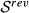. The need for a model satisfying the natural condition (10) motivated the development of bdHMMs, and indeed, condition (10) is almost their defining property: We mention without proof that under very mild assumptions on the probability distributions Ψ, any HMM satisfying (10) is a bdHMM.

#### Learning of the transition matrix and the initial state distribution

The learning problem for bdHMMs consists in maximizing the marginal likelihood of the model,

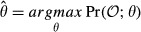


Parameter estimation in an HMM is commonly done using the Baum–Welch algorithm (Baum *et al*, 1970), an expectation-maximization (EM) algorithm (Dempster *et al*, 1977). The EM algorithm is an iterative procedure in which a target function *Q*(*θ*;*θ*^old^) is maximized with respect to the parameters *θ*, given a previous parameter guess *θ*^old^. This algorithm will converge to a local maximum of the marginal likelihood 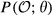. In this paragraph, we will derive an EM algorithm for the learning of the bdHMM parameters *A*,*π*.

Let 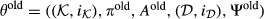 be a bdHMM. Let 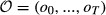 be a sequence of observations. For 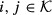, *t*=1,...,*T*, we define the posterior probabilities

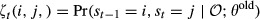
15

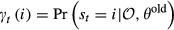
16

These posterior probabilities can be calculated efficiently using the forward probabilities 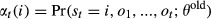 and the backward probabilities 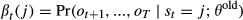, 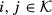. Forward and backward probabilities are calculated recursively.

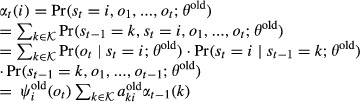
17for *t* = 1,...,*T*, and 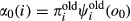. Similarly for the backward probabilities,

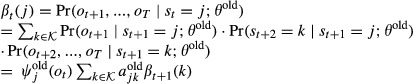
18for *t* = *T*−1,...,0, and 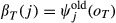. It follows that

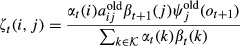
19and

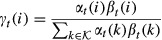
20

Note that the quantities *ζ*_*t*_(*i*,*j*) and *γ*_*t*_(*i*) are always non-negative. The target function *Q*(*θ*;*θ*^old^) is defined as the expectation of the log likelihood 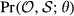, where expectation is taken with respect to the unknown hidden state sequence 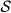 and its posterior distribution 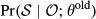,

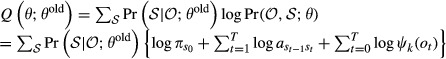
21

It can be shown that *Q*(*θ*;*θ*^old^) is a lower bound of the marginal likelihood function 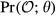 which touches the likelihood function at *θ* = *θ*^old^, that is, 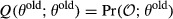 (Dempster *et al*, 1977). These properties guarantee that the iterative maximization of *Q* leads to a local maximum of 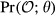. We want to maximize *Q* with respect to *A* and *π* under the constraints of a bdHMM. Using the posterior probabilities (13) and (14), and summarizing the *ψ*_*k*_ terms into one constant *c* which does not depend on *A* or *π*, the modified target function *Q* assumes a convenient form.The quantity *Q* is calculated in the E-step,

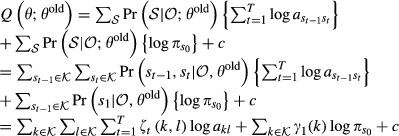
22

We calculate the Jacobian matrix and the Hessian matrix of *Q* and show that *Q* is a convex function.

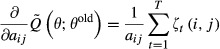
23

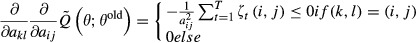
24

The Hessian matrix is a diagonal matrix with non-positive diagonal entries; hence, it is negative semidefinite. This means that *Q* is concave. The maximization of *Q* is performed under the constraints that *π* is a probability vector, *A* is a stochastic matrix and that initiation symmetry and generalized detailed balance holds. Unfortunately, these constraints define a non-convex optimization domain. Still, powerful numerical solvers for concave functions exist. In our case, we used the *ipopt* solver (Wächter & Biegler, 2006) and Rsolnp (version 1.14, http://cran.r-project.org/src/contrib/Archive/Rsolnp/Rsolnp_1.14.tar.gz). Transition probabilities might become very small or even 0, which may cause problems for the optimization since the lower boundary for the parameters is 0. Numerical optimizers tend to become very slow or even fail to converge at the boundary of the solution space. To ensure numerical stability and proper convergence, we set state transitions *a*_*ij*_=0 that drop below a certain cutoff 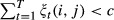. When the algorithm approximates a point of convergence, it becomes less and less likely for a transition to be removed. The EM algorithm will find an optimal point with the additional constraints that some transitions are 0. The numerical optimization approach becomes slow for very large datasets and for a high number of hidden states. In our second approach, we therefore introduce a modified lower bound function 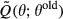 which can be maximized analytically and hence very efficiently. We iterate this maximization process in the same fashion as in the EM algorithm. Although we were not able to prove convergence of the parameter sequence, this was always the case in practice. Moreover, the results obtained by our heuristic were always identical to those obtained by the numerical solver. Our heuristic is substantially faster; for our yeast data with 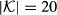 states, we achieved an acceleration by a factor of about 25.

Given a bdHMM parameter set 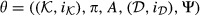, denoted by 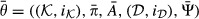, the bdHMM parameter set is defined by 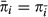, 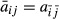, 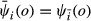, 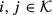, 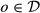. The modified target function is defined as

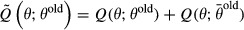
25where *Q* is defined as in (19). Since both *Q* terms in the sum in (23) are, up to some additive constant, lower bounds of the marginal likelihood function 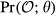, so is 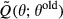.

For 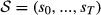, let 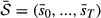. It is elementary to verify that

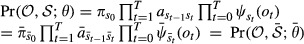
26

From (24), we deduce that

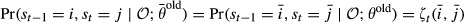
27and

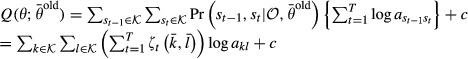
28

Equations 22 and 28 imply

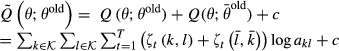


To maximize 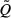 under the constraint(s) that *A* is a stochastic matrix, we introduce Lagrange multipliers 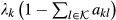, 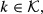 and rewrite 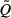 as

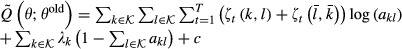
30

For 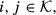, we set the partial derivatives of 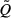 with respect to *a*_*ij*_ to zero,

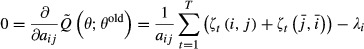
31

Multiplication by *a*_*ij*_ and summation over all equations 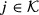 leads to

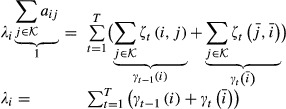
32

After substitution of (29) into (28), we solve for *a*_*ij*_.

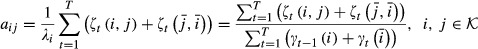
33

Let

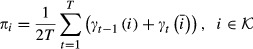
Then *π* is a probability vector which together with *A* satisfies detailed balance,

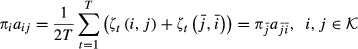


Further, *π* almost satisfies initiation symmetry:

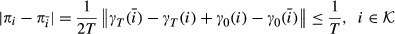


Although the vector *π* does not exactly satisfy initiation symmetry, the amount by which this symmetry is violated is generally substantially smaller than 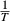. This difference is negligible for large *T*, that is, for long observation sequences.

We have developed two strategies: The first, computer-intensive strategy is to do numerical optimization using standard solvers; the second strategy is a fast heuristic. Both methods in practice lead to the same results, and they are implemented in our R/Bioconductor software package STAN.

#### Estimation of the emission probabilities

The emission distributions Ψ are also updated by maximizing the original target function *Q* in equation 21. Summarizing irrelevant terms in a constant *c*, we have

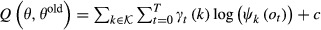


We assume multivariate Gaussian emission probabilities, 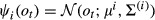, 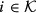, with mean 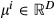 and covariance matrix 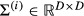. We have implemented bdHMM with multivariate Gaussian emission probabilities, since they are appropriate distributions for microarray data on a log or quasi-log scale (Huber *et al*, 2002). Moreover, the covariance matrix of multivariate Gaussians allows modeling correlations between factors in each state. This is important because factor occupancies tend to scale with the gene expression level. Such dependencies are captured by the covariance matrix. Application to sequencing-based datasets can be done by transforming the data such that it approximately follows a normal distribution (Day *et al*, 2007; Hoffman *et al*, 2012).

Setting the partial derivatives 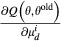, 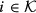, *d* ∈ *D*, to zero and solving this equation system for *μ*^*i*^ leads to (see Supplementary Information):

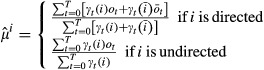


Analogously, setting the partial derivatives 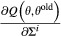, 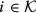, *c*,*d* ∈ *D*, to zero and solving this equation system for Σ^*i*^ leads to (see Supplementary Information):

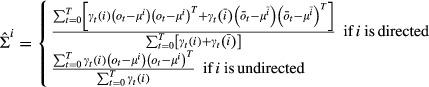


#### bdHMM learning without strand-specific observations

A bdHMM can even be learned from entirely strand-unspecific data (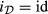). However, forward and reverse states are unidentifiable under these conditions, because 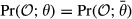. It is necessary to *a priori* annotate some positions with proper directions. We introduce the flag sequence 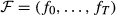, 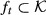, which lists the states *f*_*t*_ that are allowed at a position *t*. We then set

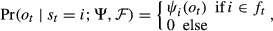
ignoring that this does not define a probability function for *i* ∉ *f*_*t*_.

In the context of transcription data, non-overlapping genes can be used to set flags allowing only forward (respectively, reverse) and undirected states.

#### *De novo* inference of state direction

Let *k* be a directed state in a bdHMM. We introduce *dir*_*k*_, a measure for the directionality of state *k* which is based on the posterior probabilities for observing *k* and respectively its conjugate 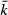 at positions *t* = 0,...,*T*. 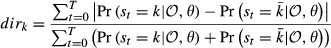
41

The score will be low if the differences in the probability for observing the forward twin state and the probability for observing the respective reverse twin state are low. It will be high if these differences are large, and thus, the direction of twin states is well distinguishable. In order to account for the overall probability of state *k*, the sum of absolute differences in the nominator in (31) is normalized by the sum over all positions *t* of the posterior probabilities for observing *k* or 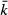. The directionality score is used to infer whether a directed state pair 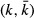 of a bdHMM truely contains directional information or whether it should be collapsed into one undirected state of a new bdHMM. Our rule of thumb is to collapse a directed state pair if *dir*_*k*_ < 0.5 (see also Results and Supplementary Fig S5).

#### Initialization of bdHMMs

If strand-specific data are available, the number of directed and undirected states can be set in an intuitive manner in advance. For the yeast data, the strand-specific expression data were first split into regions expressed on either the + or − strand and unexpressed regions. Directed state means were initialized as a k-means clustering from the expressed regions, while undirected states were initialized using k-means on the unexpressed regions. We found that initialization by k-means works very well and generally converges to a higher likelihood than multiple random starts, in agreement with Rabiner (1989). To not introduce further biases toward the k-means initialization and allow the EM to explore solutions which are further from it, covariance matrices were initially set to the covariance of the whole data and transition and initial state probabilities were initialized uniform.

In the absence of strand-specific data and without directionality annotation, we suggest to apply the directionality score that can be used as a posterior criterion to merge twin states into one undirected state, as we demonstrate for the CD4 T-cell chromatin modification data.

#### Simulations

The performance of bdHMM regarding parameter inference and state annotation on data not used for training was assessed using simulated datasets. For this purpose, we construct a transition matrix 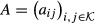 and an initial state distribution 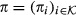 which satisfy generalized detailed balance and initiation symmetry. Choose an arbitrary transition matrix 
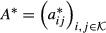
 and a stationary distribution 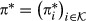, π^*^A^*^=π^*^

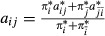


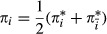


Verify that *π* is a probability vector that satisfies initiation symmetry:

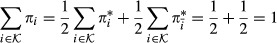


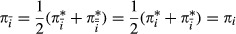


Furter, *A* is a stochastic matrix,

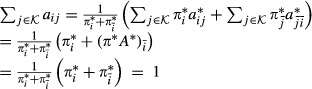
and *A* together with *π* satisfies generalized detailed balance,

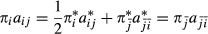
We mention that *A* is ergodic if A^*^ is ergodic.

To make our simulations realistic, we sample A^*^ as follows: Introduce an arbitrary linear order ‘≤’ on 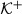 (this order is meant to describe the preferential order of events for the directed states). Then,

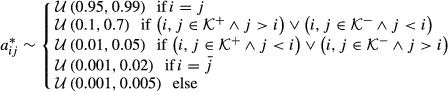
where 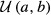 is the uniform distribution with lower bound *a* and upper bound *b*. Rows of A^*^ are then normalized to sum up to 1. An example of a simulated transition matrix is shown in Supplementary Fig S8. To get realistic simulations, emission distributions were simulated from fitted emissions of the yeast dataset, using five non-strand-specific (ChIP) and two strand-specific (expression) observation tracks.

We did 100 simulation runs. The state numbers were randomly chosen from 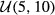 in each single run, and sequences with 15,000 observations were generated. Model parameters were initialized as follows

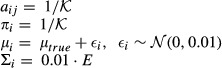
where *E* is the identity matrix. In each simulation run, models were learned on simulated observation sequences of length 1,000 (respectively, 10,000). The fitted values 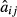 showed a good agreement with the true parameter values *a*_*ij*_, even when the model was only trained on 1,000 observations (Supplementary Fig S8). Median recovery of true hidden states not used for training was 97% when trained on 1,000 observations and 99.5% when trained on 10,000 observations (Supplementary Fig S8).

#### Clustering of state sequences

A set of valid coding genes was selected from initially 6,603 ORFs from SGD. 5,088 of them had an annotation of transcript boundaries provided by Xu *et al* (2009). Next, we selected transcripts where the TSS was located upstream and the pA site downstream of the coding region, yielding 4,687 genes. Then, state paths were extracted from the bdHMM annotation with a ±250 bp flanking region. We further selected transcripts where more than 80% of positions were annotated to the proper strand. This resulted in 4,263 genes, which were rescaled to a common length. Pairwise Hamming distances were computed, and the sequences were hierarchically clustered. The dendrogram was cut off to yield 55 clusters. Gene set enrichment analysis was carried out using mgsa (Bauer *et al*, 2010, 2011). A GO group was considered active if the posterior probability was >0.5.

#### Targeted identification of genomic features

We defined regular expressions ((*S*_+_|*T*2) + |(*S*_−_|*T*2)+) and ((*S*_−_)+(*P*/*T*1|*P*2|*P*1) + (*S*_+_)+) to search for transcripts and bidirectional promoters throughout the yeast genome, where *S*_+_ = {*PE*1_+_,*PE*2_+_,*eE*1_+_, *eE*2_+_, *eE*3_+_, *mE*1_+_, *mE*2_+_, *mE*3_+_, *mE*4_+_, *mE*5_+_, *lE*1_+_, *lE*2_+_, *lE*3_+_,*T*1_+_} defines a set containing all forward states, excluding state *P*/*T*2_+_. *S*_−_ is defined likewise. Transcripts were constrained to have a minimal length of 80 bp. We uniquely assigned the 6,068 predictions to previously annotated transcripts (Xu *et al*, 2009), using the best reciprocal hit with respect to transcript boundary distance. This yielded 4,186 uniquely assigned transcript predictions. Estimated cumulative distribution functions were computed to assess the accuracy of the predictions. The predictions of bidirectional promoters were not subsequently filtered. The newly identified transcription units were assigned a class (coding, SUT or CUT) using the SGD ORF annotation and expression data from Xu *et al* (2009).

#### *De novo* motif discovery

DNA sequences were extracted for each genomic state. To increase sensitivity of the motif search, we excluded very long and very short sequences (min. length: 150 bp, max. length: 90% quantile of sequence lengths for a state). Motif search was carried out using XXmotif (Hartmann *et al*, 2013), which uses a negative sequence set to calculate *P*-values for motif enrichment. The choice of this negative set can be crucial, since it corrects for general sequence features. We chose as negative sets upstream sequences starting at −50 bp relative to the current genomic state. A sequence motif was considered to be enriched if it had an *e*-value <10^−6^ and occurred in at least 5% of all sequences. The TOMTOM software (Gupta *et al*, 2007) was used to search databases for similar known motifs. Functional descriptions of transcription factors were obtained from SGD (Cherry *et al*, 1998).

#### Fitting a standard HMM and a bdHMM to human chromatin modifications

We fitted a bdHMM to binary chromatin modification data from Ernst and Kellis (2010), which previously had been analyzed by the ChromHMM algorithm. The Bernoulli emission probabilities learned by ChromHMM were fixed, and only transitions were updated during the learning of the bdHMM. This was done to ensure that the improvements over ChromHMM are only due to the altered modeling of the transitions. First, an HMM transition matrix was fitted using ChromHMM transitions (51 states) as initialization, whereby 10^−3^ was added to each transition probability. The bdHMM transition matrix was generated by inflating the transition matrix learned by the standard HMM to a 102 × 102 matrix. Thus, our model initially did not contain any undirected states. A flag sequence was generated from annotated GENCODE (Harrow *et al*, 2012) transcribed units (version 3c) to set directionality constraints at actively transcribed regions. The 39,447 GENCODE annotations were filtered for non-overlapping transcripts with a minimal length of 1,000 bp and minimal distance of 5,000 bp to neighboring transcripts on both strands (6,385). This set was filtered for expressed transcripts showing a median Pol II signal greater than the 25% quantile. This yielded 1,637 actively transcribed regions, which were used to generate a flag sequence, covering approximately 6% of genomic positions. After EM learning of the bdHMM transitions, the most likely state path was calculated using Viterbi decoding. Running time for bdHMM learning was 22 h using the multiprocessing version of STAN with 30 cores.

#### Comparison of bdHMM and ChromHMM

The bdHMM annotation (i.e., the Viterbi path) was compared to the ChromHMM annotation. The comparison was carried out by identifying bdHMM states with their ChromHMM counterpart having identical emission distribution. This means that conjugate forward and reverse bdHMM states are mapped to the same ChromHMM state. 83% of state annotations matched between bdHMM and ChromHMM. To account for differences in the implementation and model fitting (ChromHMM for instance uses a non-deterministic version of the online EM, while our implementation uses the standard EM algorithm) of ChromHMM and bdHMM, we also re-fitted the transitions of a standard HMM using the STAN package, which was initialized with the parameters reported by Ernst & Kellis, (2010), keeping the emission distributions fixed. The agreement between the bdHMM and re-fitted HMM annotation was 97%, showing that bdHMMs essentially add directionality to chromatin states.

## Supporting Information

Supplementary Information

Supplementary Software package

Review Process File
