# Supplementary material for: Annotation of genomics data using bidirectional hidden Markov models unveils variations in Pol II transcription cycle
Source: Mol Syst Biol. 2014 Dec 20;10(12):768. doi: 10.15252/msb.20145654 (PMC4300491; doi:10.15252/msb.20145654)
Supplement: Supplementary file 1 [file msb0010-0768-sd1.pdf]

Supplementary Information to: Annotation of genomics data by the bidirectional hidden Markov model unveils variations in Pol II transcription cycle

Benedikt Zacher, Michael Lidschreiber, Patrick Cramer, Julien Gagneur, Achim Tresch

October 23, 2014

Contents

1 Derivation of parameter updates of Gaussian emissions for twin states **2**

2 Promoter and termination states are enriched in known and new DNA motifs **3**

3 Supplementary Tables **4**

4 Supplementary Figures **5**

List of Supplementary Tables

1 Gene set enrichment (GO-groups) of clusters 4

List of Supplementary Figures

1 No obvious matching for directed states of a standard HMM 5
2A Gene-averaged signal tracks of clusters 1, 4, 5 and 6 6
2B Gene-averaged signal tracks of clusters 8, 12, 14 and 17 7
2C Gene-averaged signal tracks of clusters 19, 22, 27, 31 and 32 8
2D Gene-averaged signal tracks of clusters 33, 38, 39, 47 and 54 9
3 Upregulation of cluster 32 genes after Nrd1 depletion from the nucleus 10
4 Promoter and termination states are enriched in DNA motifs. 11
5 Directionality score for human chromatin states. 12
6 bdHMM transition on human CD4 T-cell chromatin data. 13
7 Accuracy of bdHMM state annotation. 14
8 Simulations show good performance of bdHMM. 15
9 Simulations show stable recovery of yeast bdHMM parameters. 16

1 Derivation of parameter updates of Gaussian emissions for twin states

We choose to model the emission probabilities $\psi_i(o_t) = P(o_t \mid s_t = i)$, $i \in [1; \mathcal{K}]$, as multivariate Gaussians, specified by the parameters μ^i, Σ^i with mean $\mu_i \in \mathbb{R}^D$ and covariance matrix $\Sigma_i \in \mathbb{R}^{D\times D}$,

$$\psi_i(o_t) = \mathcal{N}(o_t|\mu^i, \Sigma^i) = \frac{1}{(2\pi)^{\frac{D}{2}} \cdot |\Sigma^i|^{\frac{1}{2}}} \cdot \exp\left(-\frac{1}{2}\left(o_t - \mu^i\right)^T \cdot \left(\Sigma^i\right)^{-1} \cdot \left(o_t - \mu^i\right)\right)$$

Partial derivatives $\frac{\partial}{\partial \mu^i} \log\left(\psi_i\left(o_t\right)\right)$ and $\frac{\partial}{\partial (\Sigma^i)^{-1}} \log\left(\psi_i\left(o_t\right)\right)$ are:

$$\begin{aligned}
\frac{\partial}{\partial \mu^i} \log\left(\psi_i\left(o_t\right)\right) &= \left(o_t - \mu^i\right)^T \left(\Sigma^i\right)^{-1} \\
\frac{\partial}{\partial \left(\Sigma^i\right)^{-1}} \log\left(\psi_i\left(o_t\right)\right) &= \frac{\Sigma^i}{2} - \frac{\left(o_t - \mu^i\right)\left(o_t - \mu^i\right)^T}{2}
\end{aligned}$$

Making use of emission symmetry $\psi_i(o) = \psi_{\bar{i}}(\bar{o})$, we calculate partial derivatives $\frac{\partial}{\partial \mu^i} Q\left(\theta, \theta^{old}\right)$:

$$\begin{aligned}
\frac{\partial}{\partial \mu^i} Q\left(\theta, \theta^{old}\right) &= \frac{\partial}{\partial \mu^i}\left[\sum_{k\in\mathcal{K}}\sum_{t=1}^{T} \gamma_t\left(k\right) \log\left(\psi_k\left(o_t\right)\right)\right] \\
&= \sum_{t=1}^{T} \gamma_t\left(i\right) \frac{\partial}{\partial \mu^i}\left[\log\left(\psi_i\left(o_t\right)\right)\right] + \\
&\quad \sum_{t=1}^{T} \gamma_t\left(\bar{i}\right) \frac{\partial}{\partial \mu^i}\left[\log\left(\psi_i\left(\bar{o}_t\right)\right)\right] \\
&= \sum_{t=1}^{T} \gamma_t\left(i\right)\left(o_t - \mu^i\right)^T \left(\Sigma^i\right)^{-1} + \\
&\quad \sum_{t=1}^{T} \gamma_t\left(\bar{i}\right)\left(\bar{o}_t - \mu^i\right)^T \left(\Sigma^i\right)^{-1}
\end{aligned}$$

We set this to 0 and solve for μ^i:

$$\begin{aligned}
0 &= \sum_{t=1}^{T} \gamma_t\left(i\right)\left(o_t - \mu^i\right)^T \left(\Sigma^i\right)^{-1} + \\
&\quad \sum_{t=1}^{T} \gamma_t\left(\bar{i}\right)\left(\bar{o}_t - \mu^i\right)^T \left(\Sigma^i\right)^{-1} \\
0 &= \sum_{t=1}^{T}\left[\gamma_t\left(i\right)\left(o_t - \mu^i\right) + \gamma_t\left(\bar{i}\right)\left(\bar{o}_t - \mu^i\right)\right] \\
0 &= \sum_{t=1}^{T}\left[\gamma_t\left(i\right) o_t + \gamma_t\left(\bar{i}\right) \bar{o}_t\right] - \\
&\quad \mu^i\left(\sum_{t=1}^{T}\left[\gamma_t\left(i\right) + \gamma_t\left(\bar{i}\right)\right]\right) \\
\hat{\mu}^i &= \frac{\sum_{t=1}^{T}\left[\gamma_t\left(i\right) o_t + \gamma_t\left(\bar{i}\right) \bar{o}_t\right]}{\sum_{t=1}^{T}\left[\gamma_t\left(i\right) + \gamma_t\left(\bar{i}\right)\right]}
\end{aligned}$$

Next, we calculate partial derivatives $\frac{\partial}{\partial (\Sigma^i)^{-1}} Q\left(\theta, \theta^{old}\right)$:

$$\frac{\partial}{\partial \left(\Sigma^i\right)^{-1}} Q\left(\theta, \theta^{old}\right) = \frac{\partial}{\partial \left(\Sigma^i\right)^{-1}}\left[\sum_{k\in\mathcal{K}}\sum_{t=1}^{T} \gamma_t\left(k\right) \log\left(\psi_k\left(o_t\right)\right)\right]$$

$$= \sum_{t=1}^{T} \gamma_t(i) \left(\frac{\Sigma^i}{2} - \frac{\left(o_t - \mu^i\right)\left(o_t - \mu^i\right)^T}{2} \right) + \sum_{t=1}^{T} \gamma_t(\bar{i}) \left(\frac{\Sigma^i}{2} - \frac{\left(\bar{o}_t - \mu^i\right)\left(\bar{o}_t - \mu^i\right)^T}{2} \right)$$

Setting this to 0 and solving for Σ^i yields:

$$\begin{aligned}
0 &= \sum_{t=1}^{T} \gamma_t(i) \left(\frac{\Sigma^i}{2} - \frac{\left(o_t - \mu^i\right)\left(o_t - \mu^i\right)^T}{2} \right) \\
&\quad \sum_{t=1}^{T} \gamma_t(\bar{i}) \left(\frac{\Sigma^i}{2} - \frac{\left(\bar{o}_t - \mu^i\right)\left(\bar{o}_t - \mu^i\right)^T}{2} \right) \\
0 &= \Sigma^i \sum_{t=1}^{T} \left[\gamma_t(i) + \gamma_t(\bar{i})\right] - \\
&\quad \sum_{t=1}^{T} \left[\gamma_t(i) \left(o_t - \mu^i\right)\left(o_t - \mu^i\right)^T + \gamma_t(\bar{i}) \left(\bar{o}_t - \mu^i\right)\left(\bar{o}_t - \mu^i\right)^T \right] \\
\hat{\Sigma}^i &= \frac{\sum_{t=1}^{T} \left[\gamma_t(i) \left(o_t - \mu^i\right)\left(o_t - \mu^i\right)^T + \gamma_t(\bar{i}) \left(\bar{o}_t - \mu^i\right)\left(\bar{o}_t - \mu^i\right)^T \right]}{\sum_{t=1}^{T} \left[\gamma_t(i) + \gamma_t(\bar{i})\right]}
\end{aligned}$$

2 Promoter and termination states are enriched in known and new DNA motifs

To detect putative functional DNA sequence elements associated with certain genomic states, we performed de novo motif discovery on the nucleotide sequences underlying the bdHMM state annotation using XXmotif [2]. In order to correct for local sequence properties like codon bias we chose as negative sequence sets (not containing any motifs) the sequences with a length of 150 bp and a distance of 50 bp upstream of each state. The use of negative control sets strongly improved the sensitivity during motif search (Materials and Methods).

Promoter and termination states were enriched with sequence motifs (Supplementary Figure 4). The state P/T1, which is specific for cluster 38 genes, shows enrichment of TF-binding motifs with specific functions such as ribosome biogenesis and general regulatory function as well as previously unknown sequence motifs, in particular the highly abundant TTTTTTTTG motif present in 76% of all P/T1 sequences (Supplementary Figure 4). Termination state T1 contains motifs that are known to be involved in the 3'-end formation and pA positioning [1] and one novel motif (TTTTTTTTA). These motifs are located within the 3' UTR of genes, in accordance with the state frequency peak that we observe for state T1 (Figure 4B). The mixed state P/T2 also contains a motif associated with 3' end formation and previously unknown ones (Supplementary Figure 4). For instance, it is enriched in the motif TTTTTTTTC, which is similar to the one we found in P/T1 (Supplementary Figure 4). Finally, alternative states of the same phase of the transcription cycle were enriched for distinct motifs. For instance, the Abf1 binding motif and the Mbp1-Swi6 binding motif were specifically found in the promoter state P2 and not in the other promoter states. Together, this shows that bdHMM analysis enhances the identification of novel functional DNA elements.

3 Supplementary Tables

GO-term	size of group	#genes in cluster	probability	std. error	cluster
vesicle docking involved in exocytosis	15	8	.56	.03	**8**
regulation of cell growth	9	5	.69	.009	
mitotic sister chromatid cohesion	20	8	.85	.01	
filamentous growth	28	10	.54	.01	**14**
purine nucleotide biosynthetic process	16	9	.58	.03	
positive regulation of transcription, DNA-dependent	37	13	.61	.03	
protein import into mitochondrial matrix	19	8	.72	.03	
nitrogen compound metabolic process	80	36	.80	.01	
tRNA transcription from RNA polymerase III promoter	13	7	.83	.04	
ribosome biogenesis	137	49	.89	.01	
organic acid transport	25	11	.94	.07	
proteolysis	122	42	.94	.01	
regulation of translational initiation	10	9	.95	.03	
tRNA aminoacylation for protein translation	28	15	.97	.01	
cellular metabolic compound salvage	46	23	.98	.03	
cellular protein metabolic process	13	10	.99	.02	
peptidyl-amino acid modification	71	30	.99	.01	
nuclear pore organization	29	18	.56	.03	**17**
sodium ion transport	7	5	.60	.02	
endonucleolytic cleavage in 5'-ETS of tricistronic rRNA transcript (SSU-rRNA, 5.8S rRNA, LSU-rRNA)	23	10	.66	.04	
DNA strand elongation involved in DNA replication	7	6	.78	.02	
protein autophosphorylation	9	8	.80	.01	
G1/S transition of mitotic cell cycle	32	12	.85	.007	
ATP-dependent chromatin remodeling	18	10	.85	.02	
protein import into nucleus	30	14	.87	.02	
reproduction	77	31	.99	.003	
GPI anchor biosynthetic process	25	11	.59	.04	**19**
mitochondrial translation	72	23	.75	.03	
mitochondrial respiratory chain complex assembly	2	2	.70	.01	**22**
mitochondrial respiratory chain complex IV assembly	9	3	.84	.02	**27**
response to zinc ion	2	2	.57	.02	**32**
UMP biosynthetic process	2	2	.62	.05	**38**
regulation of pH	5	2	.64	.02	
'de novo' cotranslational protein folding	3	2	.83	.02	
termination of RNA polymerase II transcription	11	3	.86	.007	
nucleosome assembly	14	6	.97	.01	
translation	192	61	.99	.002	
autophagy	93	18	.86	.02	**39**
response to stress	106	18	.98	.008	
propionate catabolic process, 2-methylcitrate cycle	2	2	.64	.02	**54**

Supplementary Table 1: Gene set enrichment (GO-groups) of clusters using mgsa. A gene set was considered enriched if the mgsa posterior probability was > 0.5.

4 Supplementary Figures

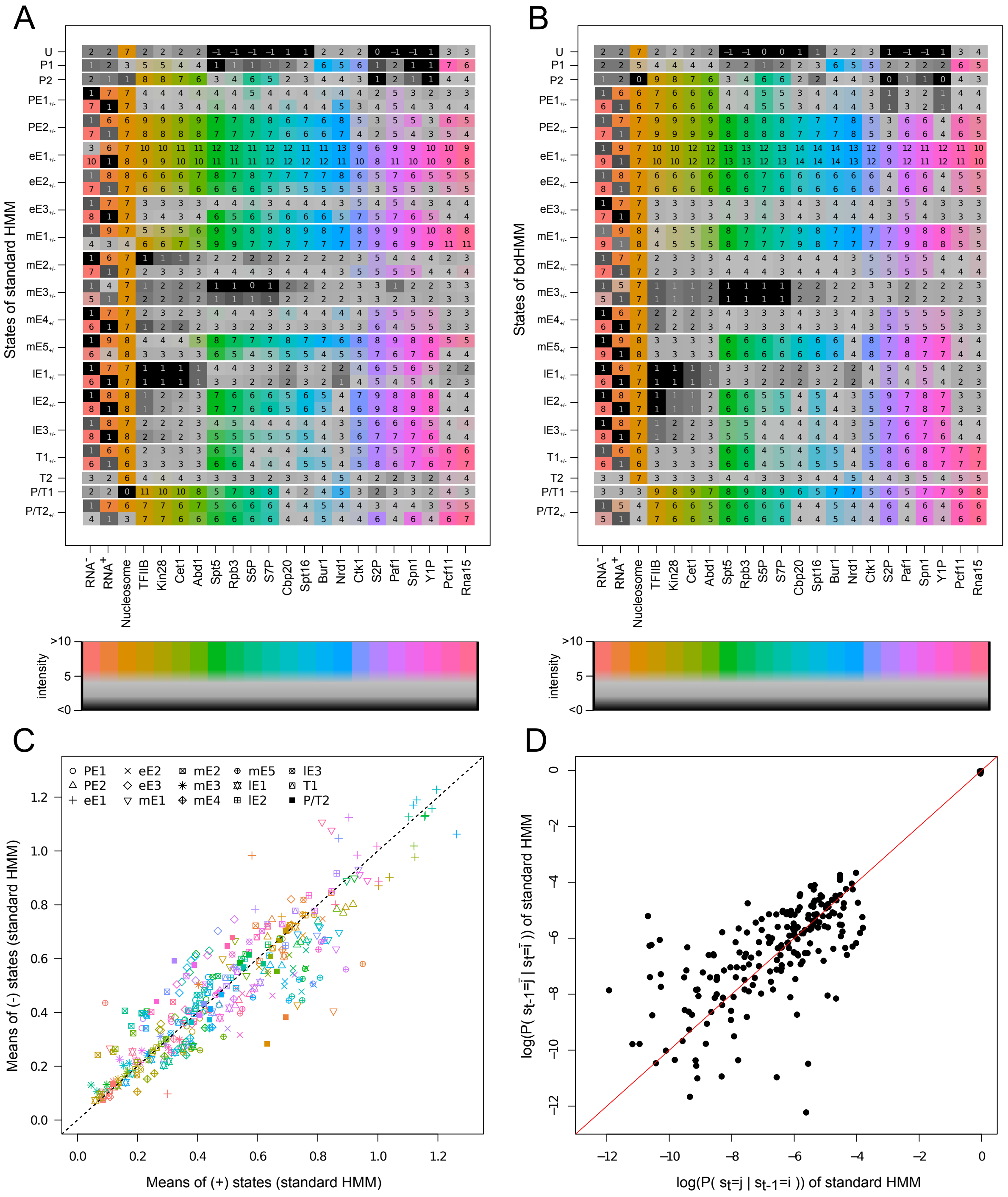

Supplementary Figure 1: No obvious matching of directed states of a standard HMM learned without symmetry conditions. While the fitted mean occupancies of the HMM differ between twin states (A), bdHMM parameters are symmetric between directions (B). (C) For each twin state pair of the standard HMM (i.e. forward and reverse direction), the mean occupancies are plotted against each other, revealing extensive asymmetry between directions. (D) The transitions of the standard HMM violate symmetry conditions between directions.

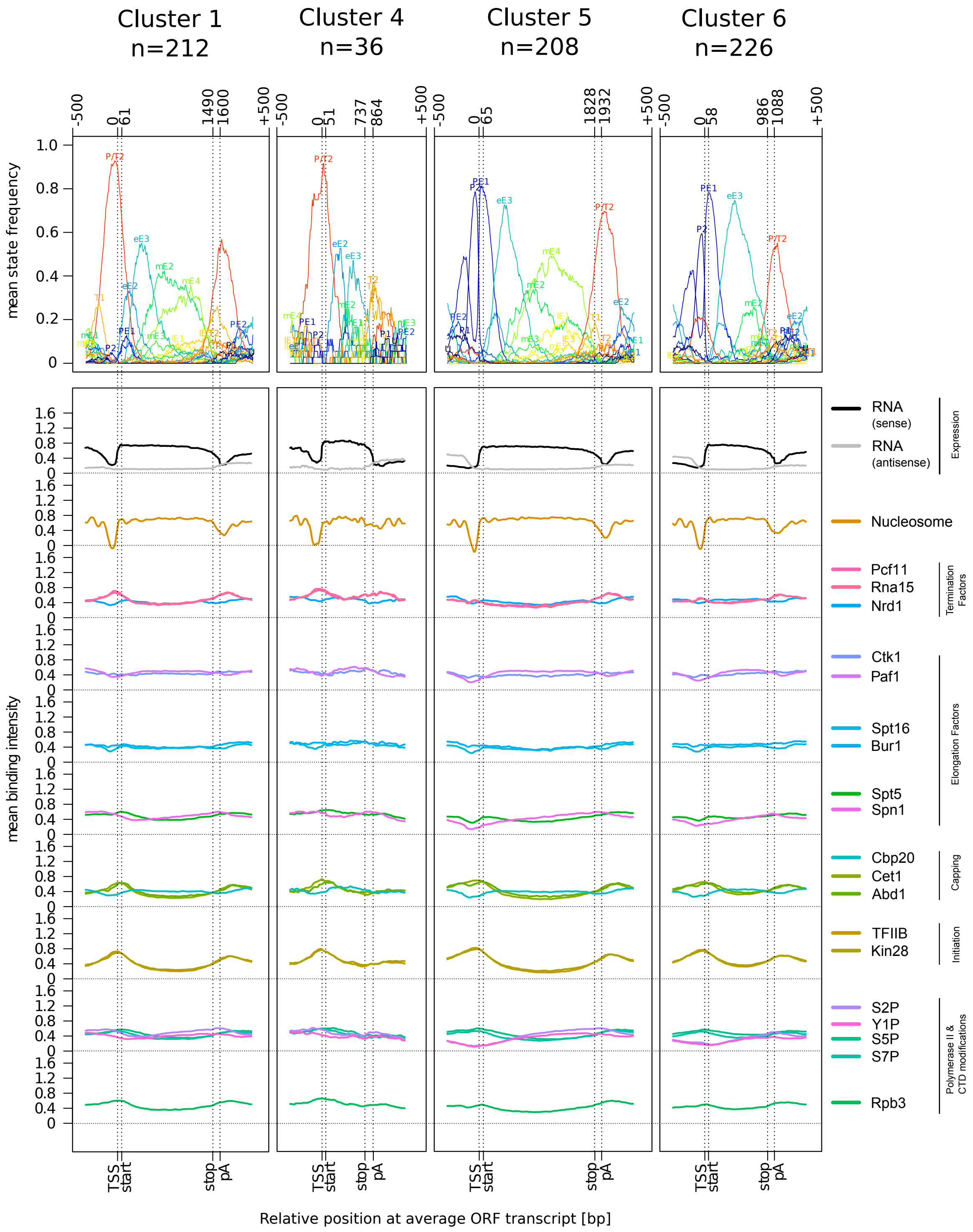

Supplementary Figure 2A: Gene-averaged signal tracks of clusters 1, 4, 5 and 6. All signal tracks are shown in sense direction of the transcript.

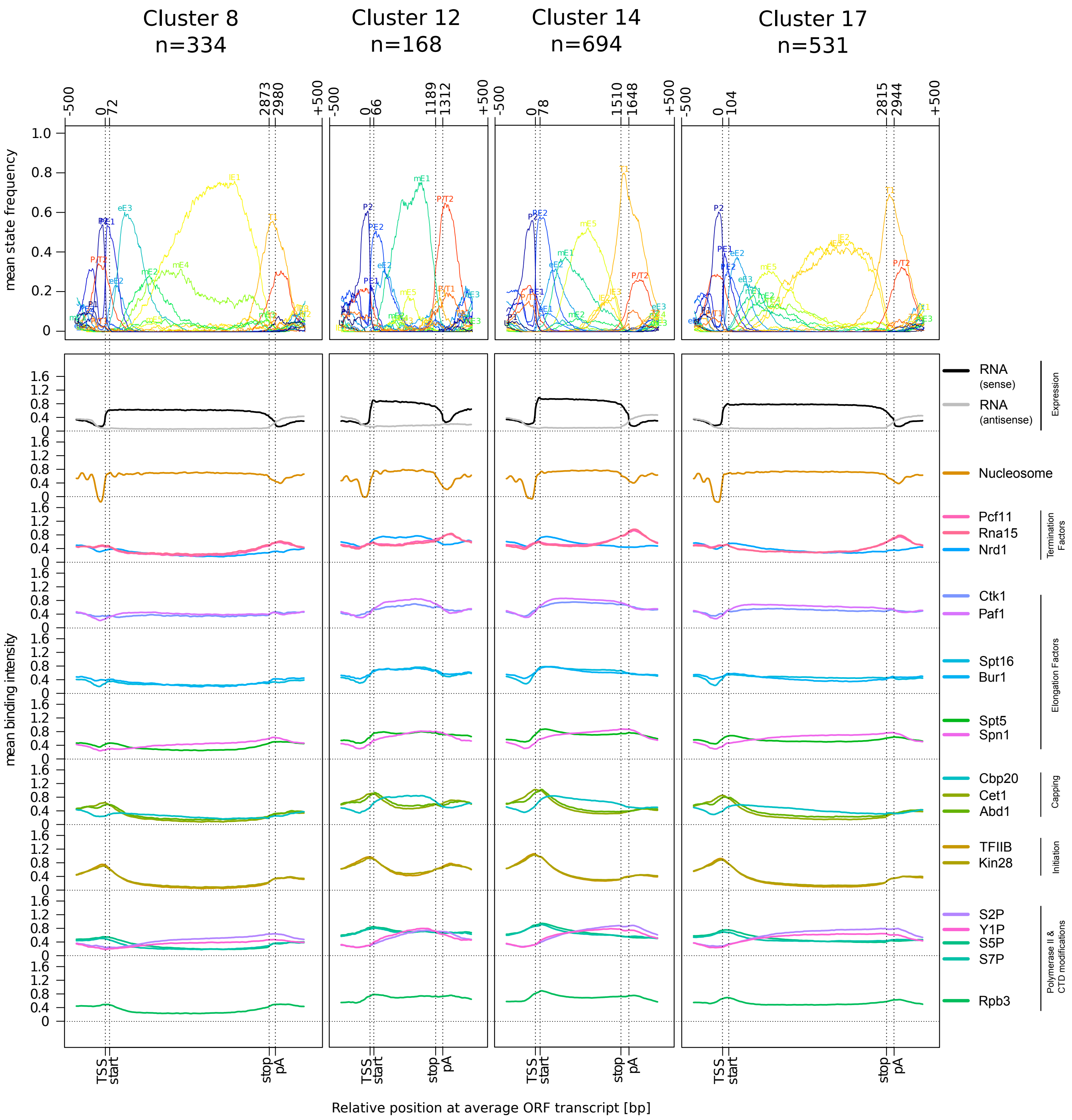

Supplementary Figure 2B: Gene-averaged signal tracks of clusters 8, 12, 14 and 17. All signal tracks are shown in sense direction of the transcript.

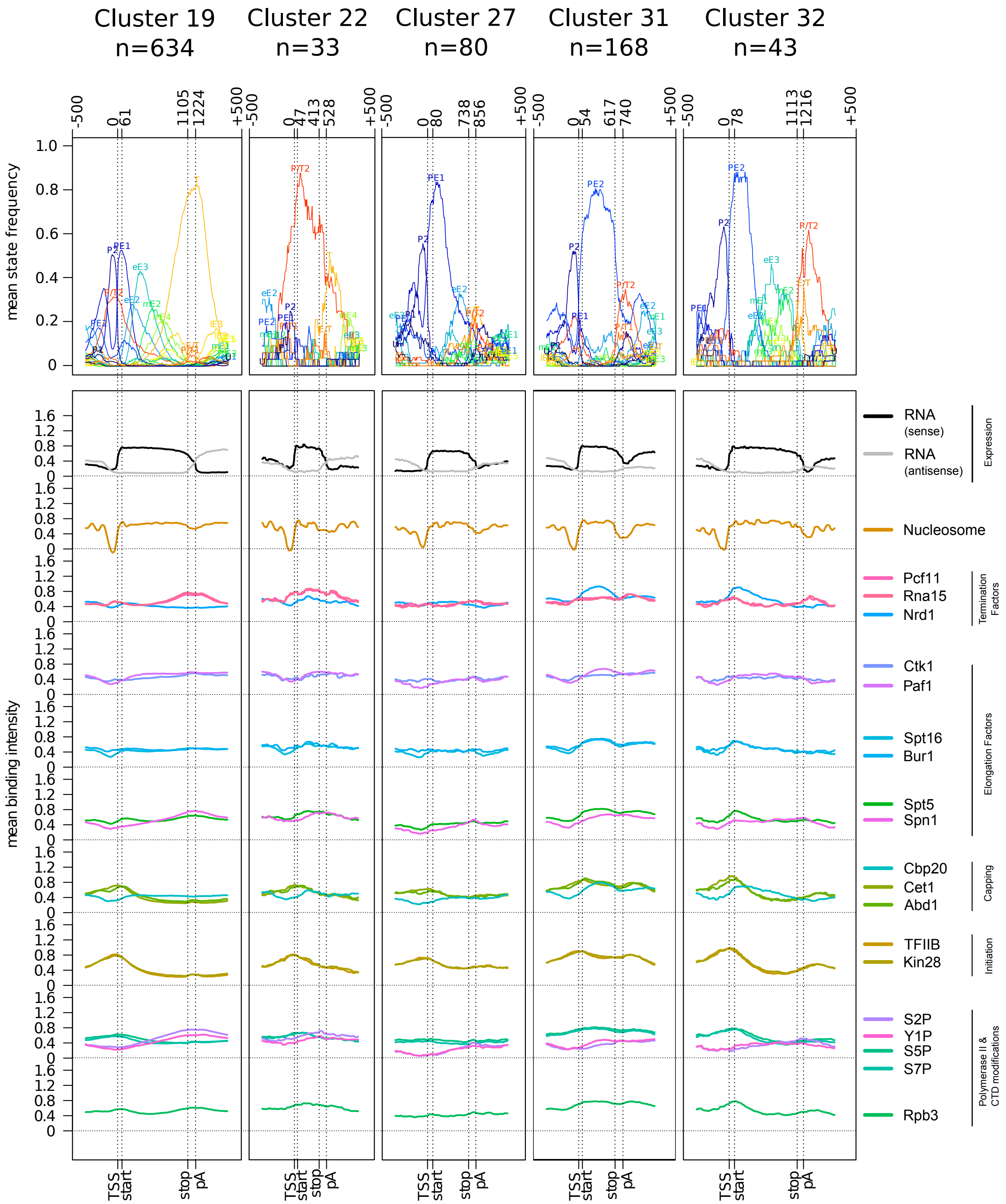

Supplementary Figure 2C: Gene-averaged signal tracks of clusters 19, 22, 27, 31 and 32. All signal tracks are shown in sense direction of the transcript.

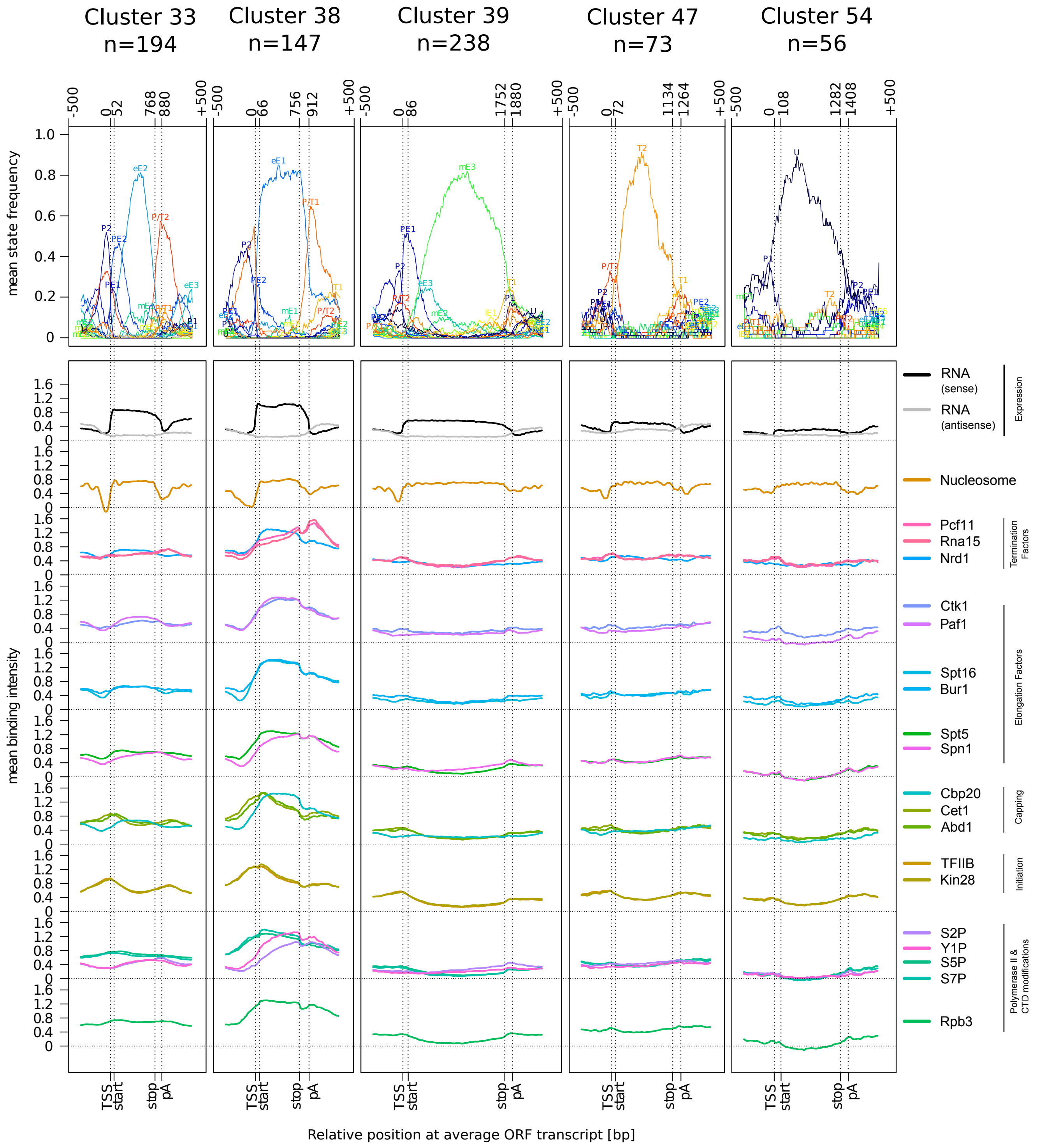

Supplementary Figure 2D: Gene-averaged signal tracks of clusters 33, 38, 39, 47 and 54. All signal tracks are shown in sense direction of the transcript.

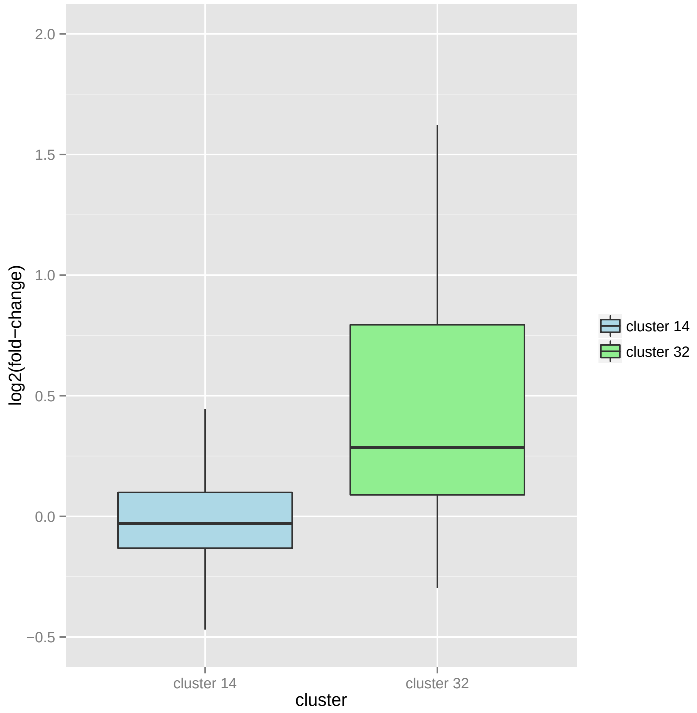

Supplementary Figure 3: Upregulation of cluster 32 genes after Nrd1 depletion from the nucleus [3]. Log2 fold-change of synthesis rate of cluster 32 and cluster 14 differ significantly (Wilcoxon-rank-sum test: $p - value = 1.5 \times 10^{-11}$). 35% (15 out of 43) of genes in cluster 32 and 6% (44 out of 694) of genes in cluster 14 have significantly different synthesis rates compared to the wild-type.

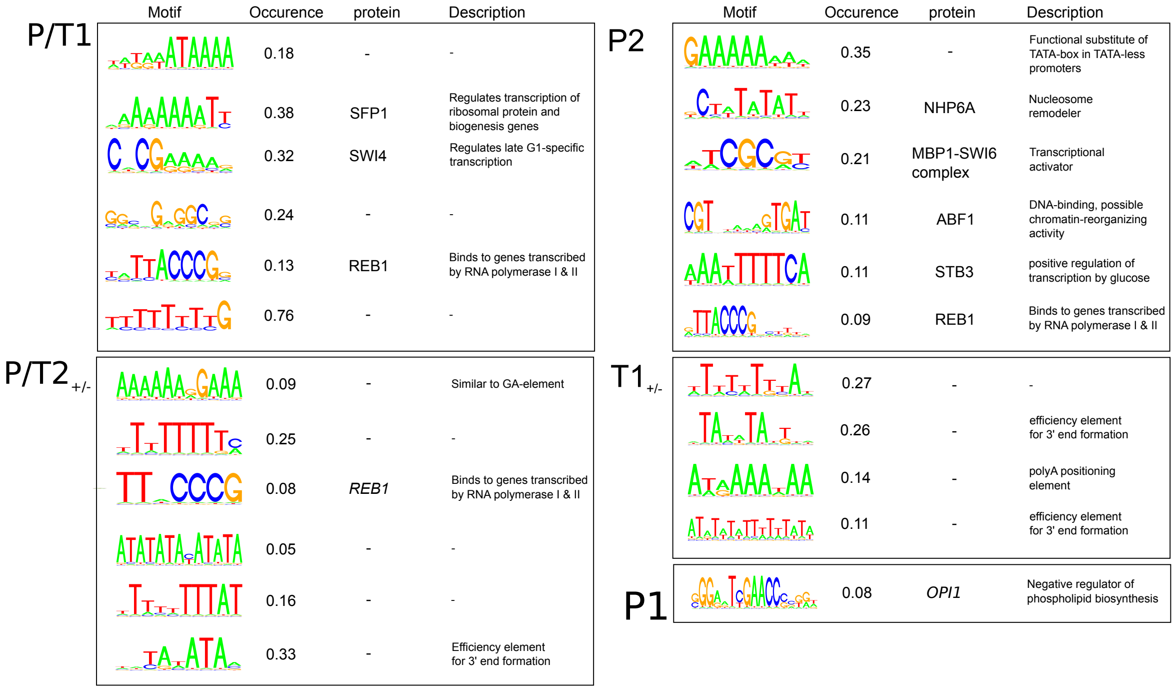

Supplementary Figure 4: Promoter and termination states are enriched in DNA motifs. De novo motif search in the DNA sequences underlying the genomic state annotation discovered motifs in promoter states and termination states. A short functional description, known binders and the frequency in the sequence set, which was used in the analysis are shown. P2 is enriched in motifs of general transcriptional regulators and chromatin remodelers. T1 contains motifs which are known to be involved in the 3' end formation and polyA positioning. P/T1 is enriched with ribosomal, cell-cycle specific and general transcription factors. P/T2 contains motifs involved in transcription initiation and termination. P1 is enriched with a single motif, that highly resembles OPI1.

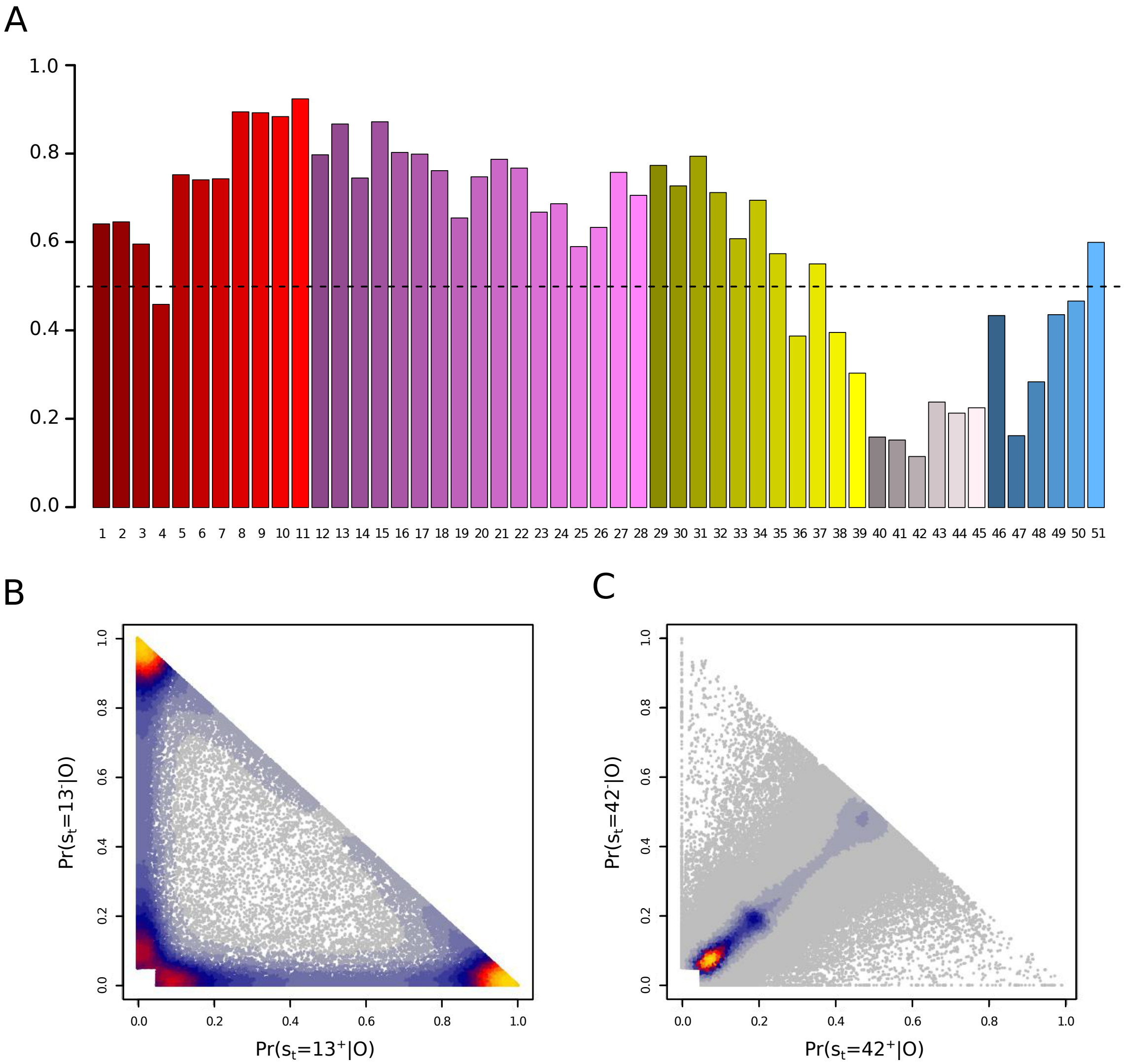

Supplementary Figure 5: Directionality score for human chromatin states is shown in (A). States with a score > 0.5 (dashed line) are classified as directed. (B) and (C) show the posterior probabilities (> 0.05) of twin states plotted against each other. Density of the points is indicated by a (heat) colorscale. Differences of posterior probabilities of directed states (e.g. state 13) are large and points are mostly located on the axes of the plot (B). In the case of undirected states (e.g. state 42), differences are small and points are located mostly on the diagonal (C).

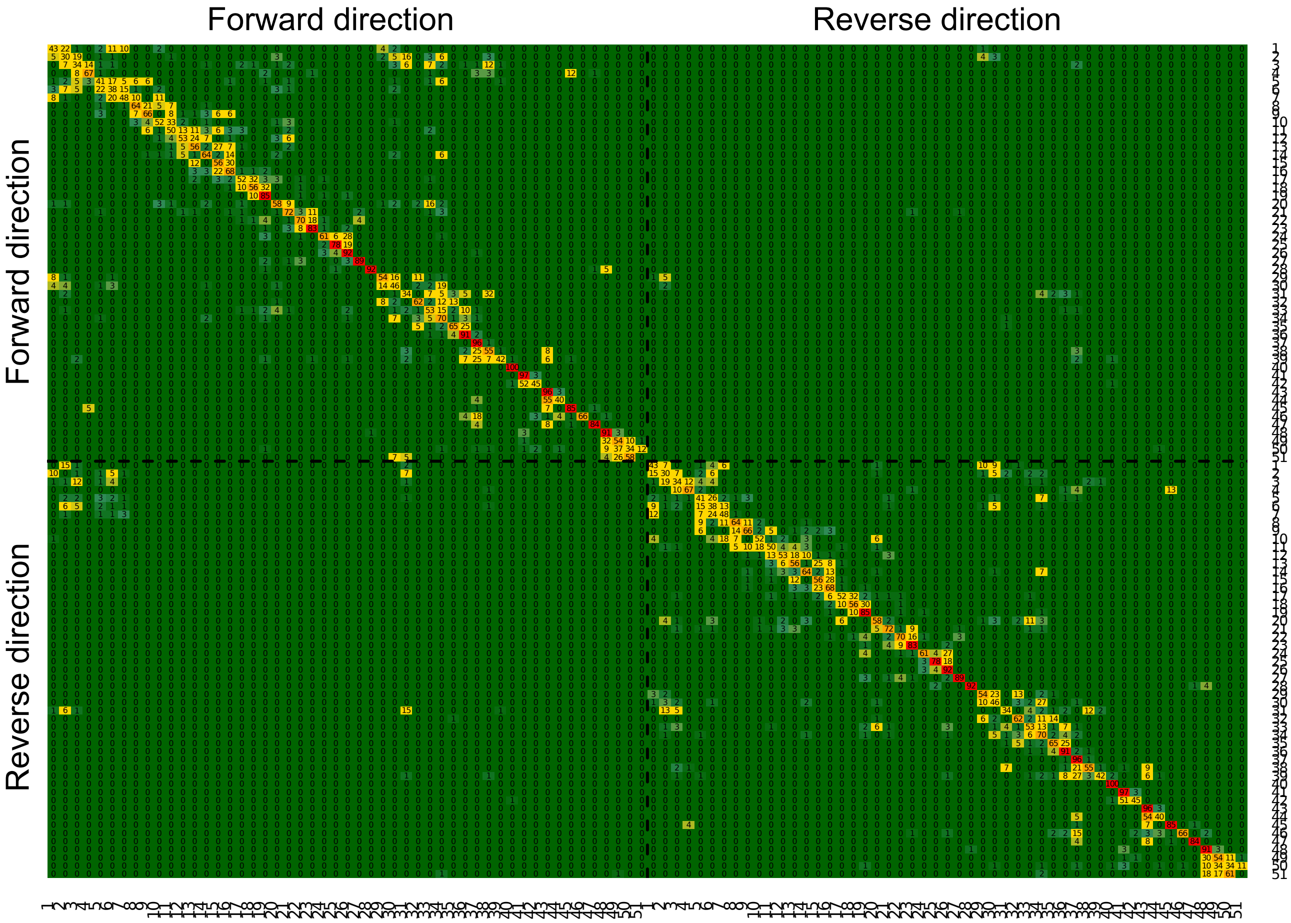

Supplementary Figure 6: bdHMM transition matrix learned on human CD4 T-cell chromatin data.

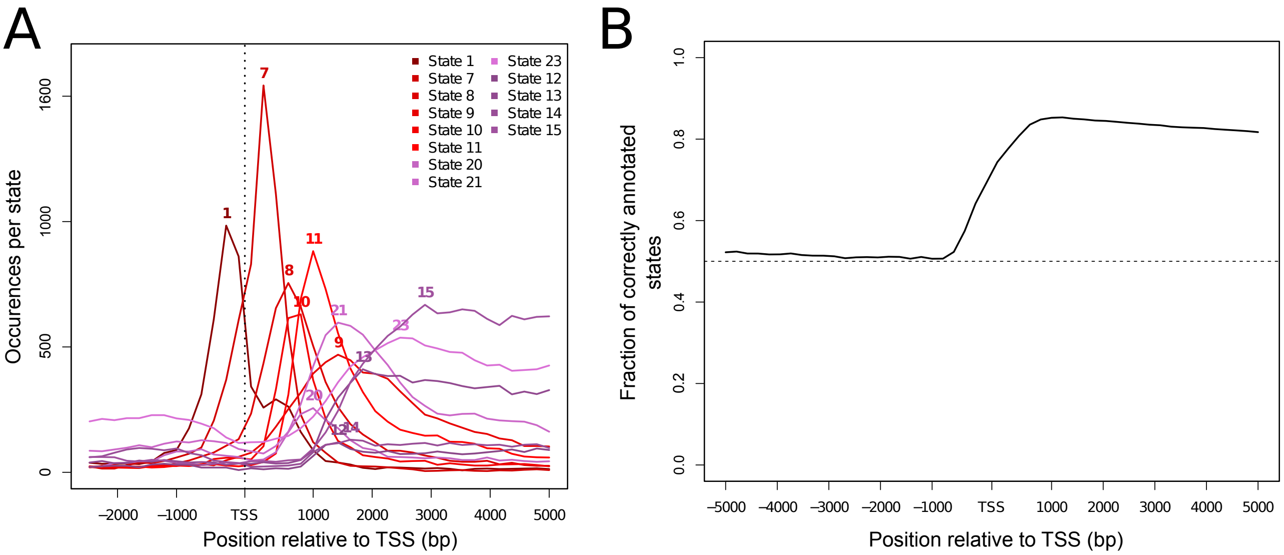

Supplementary Figure 7: (A) State frequencies of promoter and 5' proximal transcription states at RefSeq TSSs. States are shown in sense (match of state and transript directionality) direction of the respective TSS. Sense state frequencies are annotated downstream of TSSs, indicating proper state directionality. (B) Shown is the fraction of correctly annotated state directions of directed, actively transcribed chromatin states (states 1-3 and 5-28) around the TSS. Correct means that the state direction at a given position matched the transcript direction. A value of 0.5 indicates random direction assignment. While the part upstream of the TSS does not seem to contain any direction information, up to 85% of all state directions downstream of the TSS are annotated correctly.

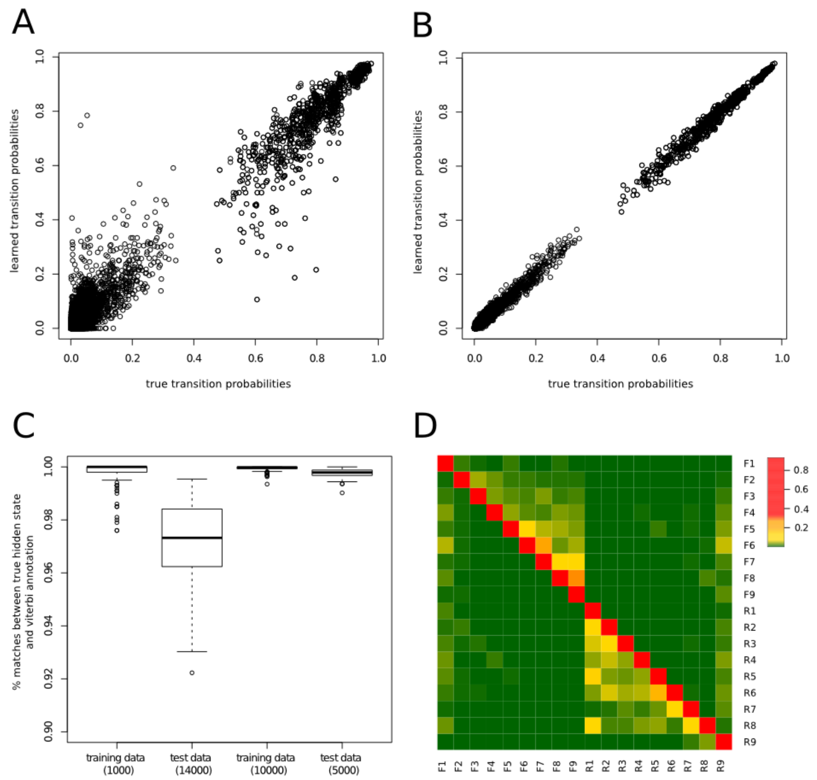

Supplementary Figure 8: Simulations show good performance of bdHMM. 100 simulations (15000 observations in each round) were carried out to assess performance of recovery of bdHMM transitions when the model was learned on 1000 (A) or 10000 (B) observations. (C) shows the respective recovery of state annotation on the training data and data not used for learning (test data). (D) shows an example of simulated bdHMM transitions.

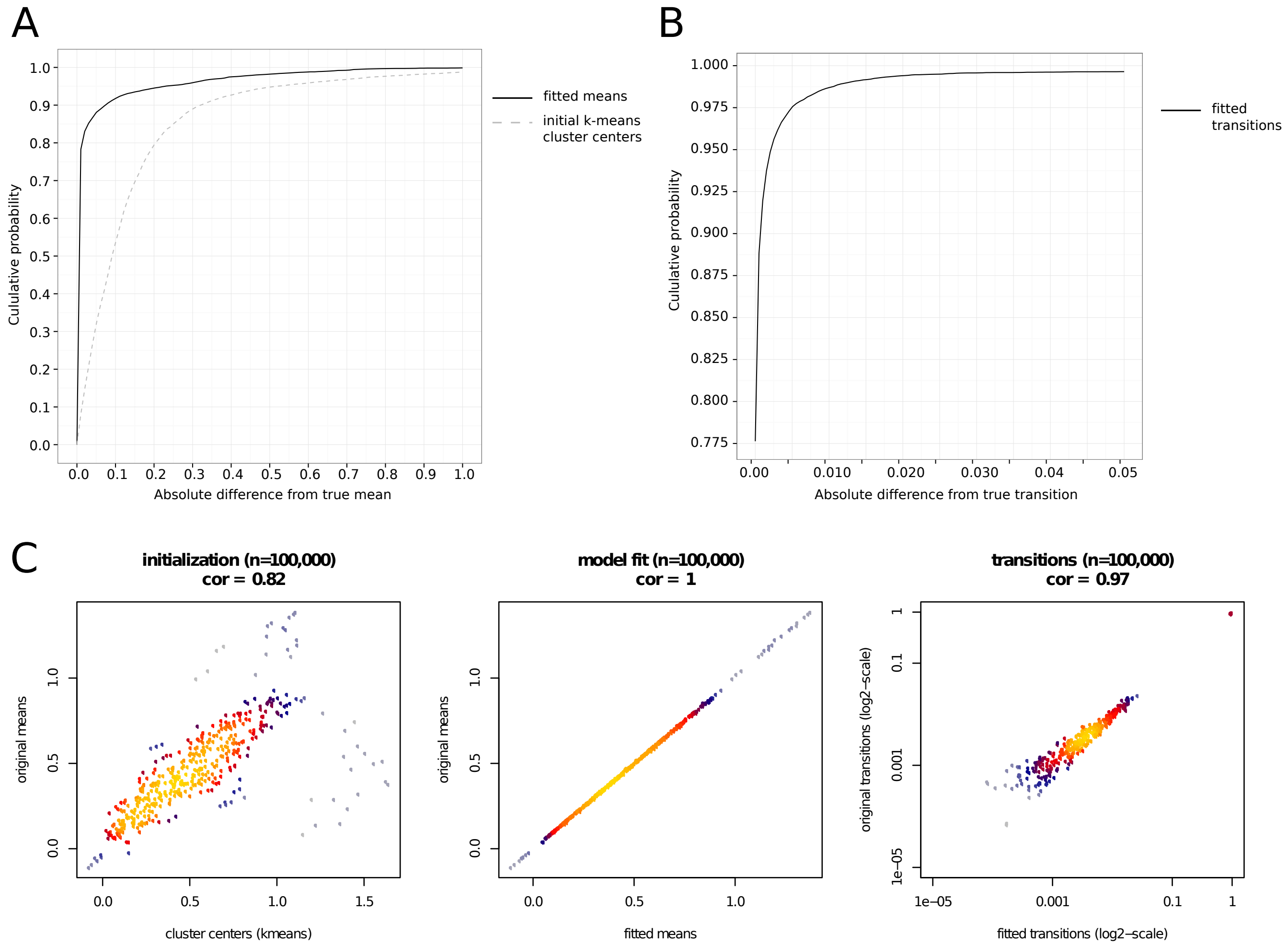

Supplementary Figure 9: Simulations from the bdHMM learned on yeast data show high accuracy of recovery of model parameters. 20 simulations (100,000 observations in each round) were carried out to assess performance of recovery of bdHMM parameters (see Materials and Methods for details on initialization). (A) Estimated cumulative distribution of absolute differences between true means, initial values derived by k-means clustering (dashed grey line) and inferred means (black line) across all 20 simulations. (B) Estimated cumulative distribution of absolute differences between real and fitted transitions. (C) shows an example simulation, with inital means derived from k-means clustering (left). Fitted means (middle) and fitted transitions (right) are plotted against the true parameters.

References

[1] Z. Guo and F. Sherman. 3'-end-forming signals of yeast mRNA. *Trends Biochem. Sci.*, 21(12):477–481, Dec 1996.

[2] H. Hartmann, E. W. Guthohrlein, M. Siebert, S. Luehr, and J. Soding. P-value-based regulatory motif discovery using positional weight matrices. *Genome Res.*, 23(1):181–194, Jan 2013.

[3] D. Schulz, B. Schwalb, A. Kiesel, C. Baejen, P. Torkler, J. Gagneur, J. Soeding, and P. Cramer. Transcriptome Surveillance by Selective Termination of Noncoding RNA Synthesis. *Cell*, Nov 2013.
