# Supplementary material for: Annotation of genomics data using bidirectional hidden Markov models unveils variations in Pol II transcription cycle
Source: Mol Syst Biol. 2014 Dec 20;10(12):768. doi: 10.15252/msb.20145654 (PMC4300491; doi:10.15252/msb.20145654)
Supplement: Supplementary file 2 [file msb0010-0768-sd2.zip › Stan Software package/STAN/inst/doc/STAN.pdf]

STrand-specific ANnotation of genomic data

Bendikt Zacher[1,2,*], Julien Gagneur[1], Achim Tresch[1,2,3]

[1] Gene Center and Department of Biochemistry, LMU, Munich, Germany
[2] Institute for Genetics, University of Cologne, Cologne, Germany
[3] Max Planck Institute for Plant Breeding Research, Cologne, Germany

*zacher (at) genzentrum.lmu.de

October 13, 2014

Contents

1 Quick start 2

2 Introduction 2

3 Integrating non-strand-specific with strand-specific data 2

4 Inferring directed genomic states from non-strand-specific data only 6

5 Initialization of (bd)HMMs 10

6 Concluding Remarks 12

1 Quick start

Learning a (bd)HMM simply requires an initial model estimate of class *HMM* or *bdHMM*. The function `fitHMM()` optimizes the model parameters and `getViterbi()` infers the most likely state annotation as the Viterbi path.

```
library(STAN)
data(example)
# Fitting a HMM
hmm_fitted = fitHMM(observations, hmm_ex)
# Fitting a bdHMM
bdhmm_fitted = fitHMM(observations, bdhmm_ex, dirFlags=flags)
# Calculating the viterbi paths
viterbi_bdhmm = getViterbi(bdhmm_fitted$hmm, observations)
viterbi_hmm = getViterbi(hmm_fitted$hmm, observations)
```

2 Introduction

STAN (**ST**rand-specic **AN**notation of genomic data) implements bidirectional Hidden Markov Models (bdHMM), which are designed for studying directed genomic processes, such as gene transcription, DNA replication, recombination or DNA repair by integrating genomic data. bdHMMs model a sequence of successive observations (e.g. ChIP or RNA measurements along the genome) by a discrete number of 'directed genomic states', which e.g. reflect distinct genome-associated complexes. Unlike standard HMM approaches, bdHMMs allow the integration of strand-specific (e.g. RNA) and non-strand-specific data (e.g. ChIP). Both cases are illustrated in this vignette. Application of *STAN* is demonstrated on real ChIP-on-chip microarray data of yeast transcription factors [1] and on ChIP-Sequencing data of human chromatin marks [2]. For a more detailed description of the concept and theory of bdHMMs, we refer ther reader to [1]. To load the package, type:

```
library(STAN)
```

3 Integrating non-strand-specific with strand-specific data

We demonstrate the application of *STAN* by inferring 'directed genomic states' from a set of non-strand-specific ChIP measurements of nine transcription factors, nucleosomes and strand-specific mRNA expression in yeast. The data (or observations) provided to *STAN* may consist of one or more observation sequences (e.g. chromosomes), which are contained in a *list* of (position x experiment) matrices. `yeastTF_databychrom_ex` is a list containing one observation sequence, comprising 1,375 successive observations from chromosome 4 measured with a high resolution tiling array in yeast [1]:

```
data(yeastTF_databychrom_ex)
str(yeastTF_databychrom_ex)
## List of 1
##  $ chr04: num [1:1299, 1:12] 0.691 0.691 0.66 0.659 0.658 ...
##   ..- attr(*, "dimnames")=List of 2
##   .. ..$ : NULL
##   .. ..$ : chr [1:12] "Nucleosome" "Rpb3" "S5P" "S2P" ...
data(yeastTF_probeAnno_ex)
str(yeastTF_probeAnno_ex)
## List of 1
```

```
##  $ chr04: num [1:1299] 1214609 1214617 1214625 1214633 1214641 ...
```

A bdHMM models a directed process using the concept of twin states, where each genomic state is split up into a pair of twin states, one for each direction (e.g. sense and antisense in context of transcription). Those twin state pairs are identical in terms of their emissions (i.e. they model the same genomic state) [1].
In order to fit a (bd)HMM to the yeast example data, the number of states needs to be predefined, which we set to $K = 12$ (5 directed twin state pairs and 2 undirected states, see also section "Initialization of (bd)HMMs"). In *STAN*, these states need to be labeled according to the state directionality. Directed states 1 to 5 are split up into twin states F1 to F5 (forward direction) and R1 to R5 (reverse direction). Undirected states are labeled as U1 and U2:

```
stateLabel = c("F1", "F2", "F3", "F4", "F5", "R1", "R2", "R3", "R4", "R5", "U1", "U2")
```

In order to infer model parameters, initial estimates are required for

- the emssion distributions. In this case we choose to model the continuous data with multivariate gaussian distributions. Thus initial estimates for the means μ_i and covariance matrices Σ_i are needed. $(i \in [1; K])$
- the state transition probabilities: $a_{ij} = P(s_t = j|s_{t-1} = i)$. $(i, j \in [1; K])$
- the initial state probabilities: $P(s_1 = i)$. $(i \in [1; K])$

Initial estimates for the multivariate gaussians were computed in advance to ensure reproducibility of this vignette. Σ_i was set to the covariance of the whole dataset and μ_i was initialized using kmeans clustering (see section "Initialization of (bd)HMMsusing" for more details). The function `HMMEmission()` creates an instance of the *HMMEmission* class:

```
nStates = 12
data(yeastTF_initMeans)
data(yeastTF_initCovs)
gaussEmission = HMMEmission(type="Gaussian",
                            parameters=list(mean=yeastTF_initMeans, cov=yeastTF_initCovs),
                            nStates=nStates)
gaussEmission
## An object of class "HMMEmission"
##  type: Gaussian
##  dim: 12
##  nStates: 12
```

Transitions and initial state probabilities are initialized uniform:

```
transMat = matrix(1/nStates, nrow=nStates, ncol=nStates)
initProb = rep(1/nStates, nStates)
```

Lastly, the directionality (or strand-specificity) of observations needs to be defined. Columns 1 to 10 of the observation matrix contain undirected ChIP signal, which is indicated by 0, while the pair of strand-specific (expression) observations in column 11 and 12 is indicated by 1:

```
dirobs = as.integer(c(rep(0,10), 1, 1))
```

Note that strand-specific observation tracks must occur as integer pairs in `dirobs`. These pairs are makred by increasing, matching integers and thus an additional strand-specific observation pair would be indicated by a pair of 2.
Now we initialize a bdHMM object with the `bdHMM()` function, which creates a *bdHMM* object and call `fitHMM()` to optimize the initial parameter set using the EM algorithm.

```
bdhmm = bdHMM(initProb=initProb, transMat=transMat,
        emission=gaussEmission, nStates=nStates,
```

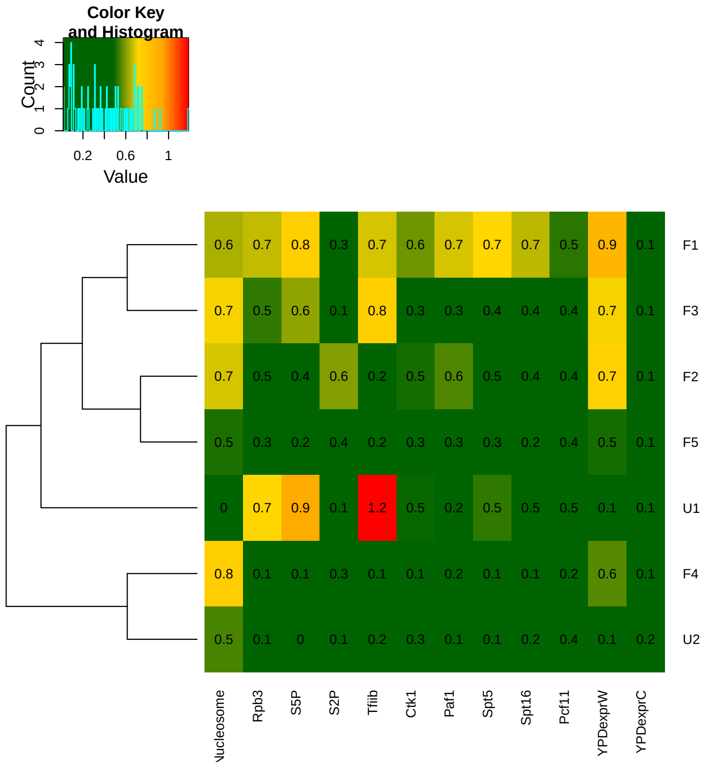

Figure 1: Mean signal of forward and undirected genomic states. U2 is an intergenic state with low signal for all data tracks except the Nucleosomes. Promoter state U1 shows a characteristic depletion of Nucleosomes and enrichment of TFIIB and (serine 5 phosphorylated) PolII (Rpb3). Directed genomic states F1, F2 and F3 model transcription of highly expressed genes, while F4 and F5 transcription of lowly expressed genes.

```
        status="initial", stateLabel=stateLabel,
        transitionsOptim="analytical", directedObs=dirobs)
bdhmm_fitted = fitHMM(yeastTF_databychrom_ex, bdhmm, maxIters=100, verbose=FALSE)
```

The result is a list containing the trace of the log-likelihood during model fitting and the resulting *bdHMM* object. The mean values for all forward and undirected states can be extracted from the result and plotted as follows (see Figure 1):

```
means_fitted = do.call("rbind",bdhmm_fitted$hmm@emission@parameters$mean, )[c(1:5, 11:12),]
library(gplots)
heat = c("dark green", "dark green", "dark green", "gold", "orange", "red")
colfct = colorRampPalette(heat)
colpal = colfct(200)
colnames(means_fitted) = colnames(yeastTF_databychrom_ex[[1]])
rownames(means_fitted) = c(paste("F", 1:5, sep=""), paste("U", 1:2, sep=""))
heatmap.2(means_fitted, col=colpal, trace="none", cexCol=0.9, cexRow=0.9,
          cellnote=round(means_fitted,1), notecol="black", dendrogram="row",
          Rowv=TRUE, Colv=FALSE, notecex=0.9)
```

Based on the distribution of state means, one can already derive a thorough description of state properties (see Figure 1). The optimized model can now be used to infer a directed genomic state annotation. This can be done in two ways using *STAN*. `getPosterior()` calculates the posterior probability of each state at each genomic position and therefore provides soft assignments for the most likely state path (note that a hard assignment can easily be derived by choosing at each position the state with maximal posterior probability $s_t = argmax_i[Pr(s_t = i|\mathcal{O}, \theta)]$ for all t, where $\mathcal{S} = (s_1, s_2, ..., s_T)$,

is the state path, $\mathcal{O} = (o_1, o_2, ..., o_T)$ the observation sequence and θ the model parameters). Another way is to find an $\mathcal{S}$, which maximizes $Pr(\mathcal{S}, \mathcal{O}|\theta)$, which is also known as the Viterbi path. This is made available by the *STAN* function `getViteri()`:

```
yeastTF_viterbi = getViterbi(bdhmm_fitted$hmm, yeastTF_databychrom_ex)
str(yeastTF_viterbi)
```

For visualization of the Viterbi path, together with the data, we can use the *Gviz* package, an R-based Genome Browser (for more detailson plotting genomic data, please check the *Gviz* manual or vignette). First, we retrieve the genomic annotation at the region of interest.

```
library(Gviz)
chr = "chrIV"
gen = "sacCer3"
gtrack <- GenomeAxisTrack()
yeastTF_SGDGenes <- UcscTrack(genome = gen, chromosome = chr,
        track = "SGD Gene", from=1217060, to=1225000, trackType = "GeneRegionTrack",
        rstarts = "exonStarts", rends = "exonEnds", gene = "name",
        symbol = "name", transcript = "name", strand = "strand",
        fill = "blue", name = "SGD Genes", col="black")
```

Next, we need to convert our data tracks to *AnnotationTrack* objects, which store the data together with parameters for plotting:

```
faccols = hcl(h = seq(15, 375 - 360/dim(yeastTF_databychrom_ex$chr04)[2],
              length = dim(yeastTF_databychrom_ex$chr04)[2])%%360, c = 100, l = 65)
names(faccols) = colnames(yeastTF_databychrom_ex$chr04)

dlist=list()
for(n in colnames(yeastTF_databychrom_ex$chr04)) {
    dlist[[n]] = DataTrack(data = yeastTF_databychrom_ex$chr04[,n],
                 start = yeastTF_probeAnno_ex$chr04,
                 end = yeastTF_probeAnno_ex$chr04+8,
                 chromosome = "chrIV", genome=gen,
                 name = n, type="h", col=faccols[n])
}
```

The genomic state annotation is then converted to an *AnnotationTrack* object by the use of the `Rle()` and `GRanges()` function:

```
library(GenomicRanges)
library(IRanges)
myViterbiDirs = list(F=c("F1", "F2", "F3", "F4", "F5"), U=c("U1", "U2"),
                     R=c("R1", "R2", "R3", "R4", "R5"))
myViterbiPanels = list()
cols = rainbow(7)
cols = cols[c(1:5,1:5,6:7)]
names(cols) = stateLabel

for(dir in c("F", "U", "R")) {
    myPos = yeastTF_probeAnno_ex$chr04 >= 1217060 & yeastTF_probeAnno_ex$chr04 <= 1225000
    myRle = Rle(yeastTF_viterbi$chr04[myPos])
    currItems = which(myRle@values %in% myViterbiDirs[[dir]])
```

```
    start = yeastTF_probeAnno_ex$chr04[myPos][start(myRle)][currItems]
    width = myRle@lengths[currItems]
    ids = as.character(myRle@values[currItems])
    values = as.character(myRle@values[currItems])

    myViterbiPanels[[dir]] = AnnotationTrack(range=GRanges(seqnames=rep("chrIV",
        length(currItems)), ranges=IRanges(start=start, width=width*8, names=values)),
        genome=gen, chromosome=chr, name=paste("Viterbi\n", "(", dir, ")", sep=""),
        id=ids[order(start)], shape="box",fill=cols[values[order(start)]], col="black",
        stacking="dense")
}
```

Finally, relative track sizes are assigned and the region of interest is plotted using `plotTracks()` (see Figure 2):

```
sizes = rep(1,17)
sizes[14] = 2
sizes[15:17] = 0.7
sizes[1] = 1.3
plotTracks(c(list(gtrack), dlist, list(yeastTF_SGDGenes), myViterbiPanels),
           from=1217060, to=1225000, sizes=sizes, showFeatureId=TRUE, featureAnnotation="id",
           fontcolor.feature="black", cex.feature=0.7, background.title="darkgrey", showId=TRUE)
```

4 Inferring directed genomic states from non-strand-specific data only

This section demonstrates inference of directed states using *STAN* on ChIP-Sequencing data, where no strand-specific data is available. Moreover, in this scenario we do not decide a priori, which states are modeled as directed and which as undirected. Instead all states are initially directed and a score based on the posterior state distributions is then applied after the model fitting to decide if a state is directed or not.
As an example we use human chromatin modification data from [2]. The data consists of 13 signal tracks, which we model using 4 directed twin state pairs (i.e. overall 8 states, see also Figure 4):

```
data(humanCD4T_signal_ex)
data(humanCD4T_probeAnno_ex)
nStates = 8
stateLabel = c("F1", "F2", "F3", "F4", "R1", "R2", "R3", "R4")
```

When no strand-specific data is available, the directionality needs to be indicated by the flags sequence. This sequence marks positions which are known to be either a specific direction or undirected (e.g. at known genes). `flagsCD4T_ex` stores these sequences as character vectors (one for each observation sequence), where "F" stands for forward direction or undirected, "R" for reverse direction or undirected and "U" for any of the three:

```
data(humanCD4T_flags_ex)
humanCD4T_flags_ex$chr7[600:650]
##  [1] "U" "U" "U" "U" "U" "U" "U" "U" "U" "U" "U" "U" "U" "U" "R" "R" "R" "R" "R" "R" "R"
## [22] "R" "R" "R" "R" "R" "R" "R" "R" "R" "R" "R" "R" "R" "R" "R" "R" "R" "R" "R" "R" "R"
## [43] "R" "R" "R" "R" "R" "R" "R" "R" "R"
```

In this example the flag sequence initializes directionality at a region covered by one annotated gene (in reverse direction,

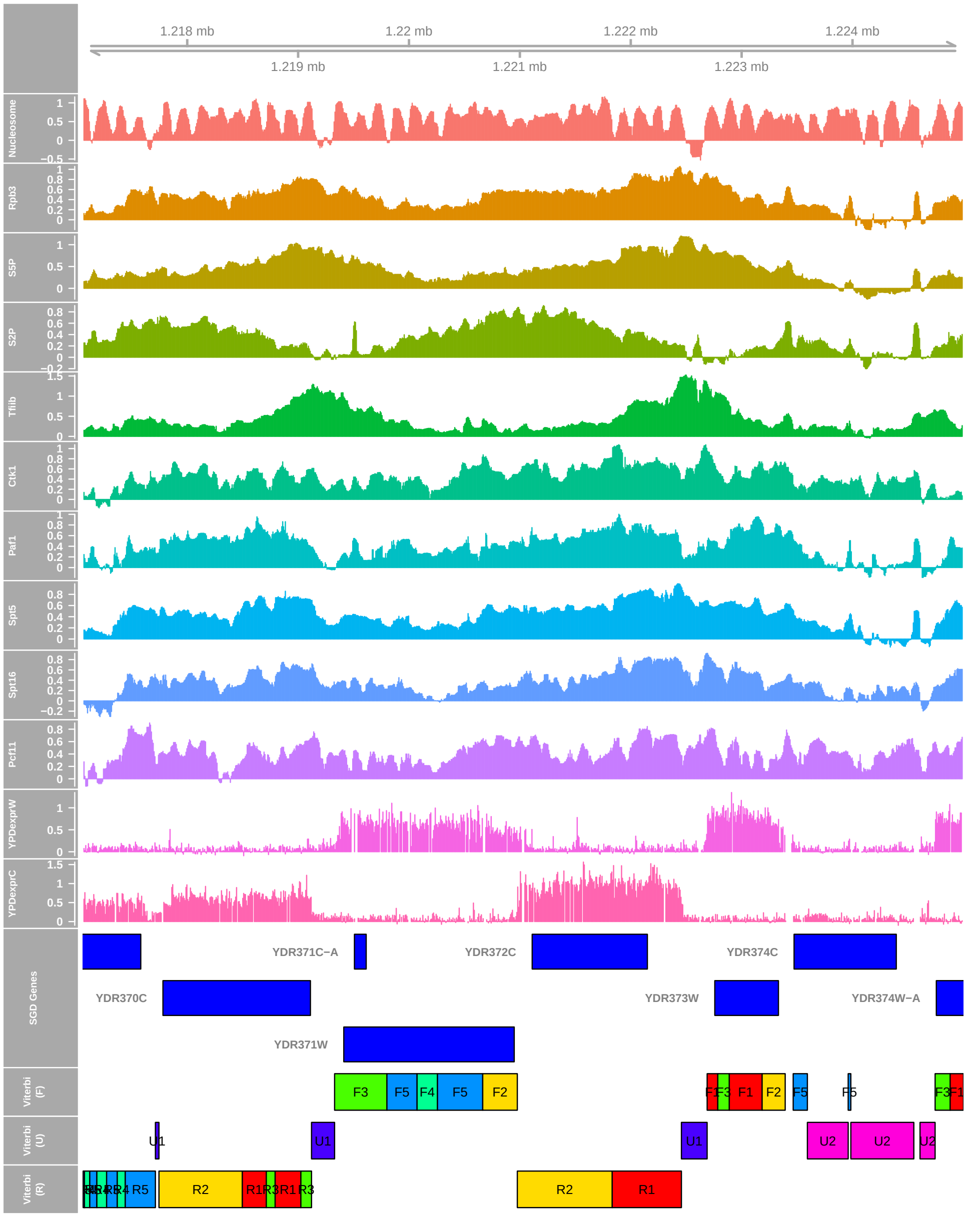

Figure 2: The example data is shown with directed genomic state annotation and SGD genes. The bdHMM state annotation properly recovers transcript structure and directionality. States occur at characteristic positions relative to transcription start and termination sites, i.e. refeltcing the different phases of the transcription cycle.

see also Figure 4). Gaussian means are again initialized using k-means clustering, covariances are set to the covariance of the whole data set and initial and transition probabilities are initialized uniform. We build a *bdHMM* object and optimize parameters:

```
data(humanCD4T_initMeans)
data(humanCD4T_initCovs)
gaussEmission = HMMEmission(type="Gaussian",
parameters=list(mean=humanCD4T_initMeans, cov=humanCD4T_initCovs),
nStates=nStates)
transMat = matrix(1/nStates, nrow=nStates, ncol=nStates)
```

```
initProb = rep(1/nStates, nStates)
dirobs = as.integer(rep(0,13))
bdhmm = bdHMM(initProb=initProb, transMat=transMat,
emission=gaussEmission, nStates=nStates,
status="initial", stateLabel=stateLabel,
transitionsOptim="analytical", directedObs=dirobs)
mybdHMM_CD4T = fitHMM(humanCD4T_signal_ex, bdhmm, maxIters=1000, dirFlags=humanCD4T_flags_ex)
```

Having fitted the bdHMM, we want to decide which twin states are directed and which are undirected. This can be done using a score based on the posterior probailities of each state pair [1]. The score will be low if the differences in the probability for observing the forward twin state and the probability for observing the respective reverse twin state is low. It will be high if this differences is large and thus the directionality of twin states is well distinguishable.
We calculate the posterior state distributions using `getPosterior()` and derive a directionality score for each twin state pair, which is plotted in Figure 3:

```
posterior_bdhmm = getPosterior(mybdHMM_CD4T$hmm, obs=humanCD4T_signal_ex)

myTwins = 5:8
score = rep(0,4)
for(i in 1:(nStates/2)) {
    numer = sum(abs(posterior_bdhmm$chr7[,i]-posterior_bdhmm$chr7[,myTwins[i]]))
    denom = sum((posterior_bdhmm$chr7[,i]+posterior_bdhmm$chr7[,myTwins[i]]))
    score[i] = numer/denom
}

names(score) = c("F1/R1", "F2/R2", "F3/R3", "F4/R4")
barplot(score, col=rainbow(4), cex.names=0.8, ylim=c(0,1))
abline(h=0.5, lty=3)
```

As rule of thumb we use 0.5 as cutoff for state directionality. In our example, states F1/R1 and F2/R2 are directed, while states F3/R3 and F4/R4 are undirected.

Next, the state annotation is inferred using `getViterbi()`. One could also calculate a reduced model using two directed twin state pairs and two undirected states, but for the sake of simplicity of this vignette, we simply collapse states F3/R3 and F4/R4 into U1 and U2 in the state annotation:

```
humanCD4T_viterbi = getViterbi(mybdHMM_CD4T$hmm, humanCD4T_signal_ex)
stateLabel2 = c("F1", "F2", "U1", "U2", "R1", "R2", "U1", "U2")
humanCD4T_viterbi_collapsed = lapply(humanCD4T_viterbi,
                              function(chrom) factor(stateLabel2[chrom]))
```

Plotting the results (see previous section for more details) revelas that directed state F1/R1 associates with the 5' end of genes, while state F2/R2 associates with the 3' end of genes (Figure 4). State U1, which has some directionality, but still below the cutoff is located upstream of genes, while state U2 (with a directionality score of 0) models intergenic, unbound regions.

```
library(Gviz)
data(humanCD4T_ucscGenes)
chr = "chr7"
gen = "hg18"
gtrack <- GenomeAxisTrack()
humanCD4T_ideogramChr7 <- IdeogramTrack(genome = gen, chromosome = chr)
```

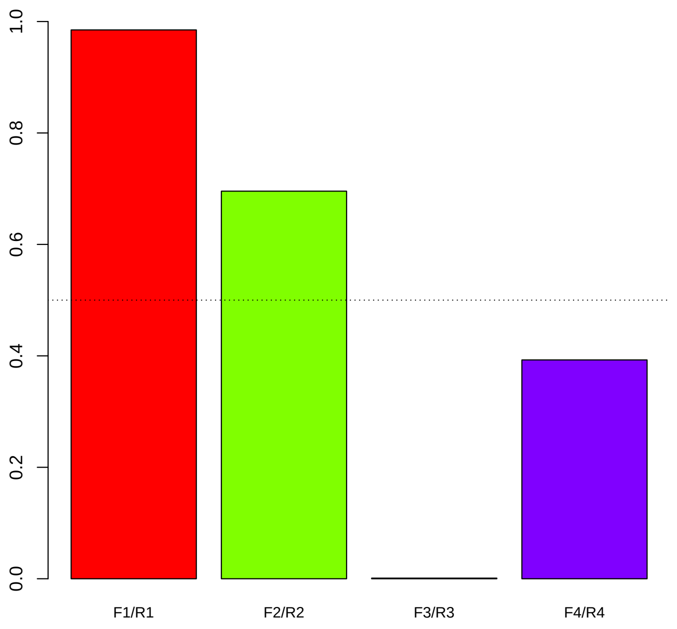

Figure 3: Directionality score of four bdHMM states fitted to human CD4 T-cell chromatin data. According to a cutoff of 0.5, state F1 and F2 are directed. State F3 and F4 are undirected.

```
faccols = c("#FB9EB1", "#FB9EB1", "#FB9EB1", "#FB9EB1", "#FB9EB1", "#DAB36A", "#DAB36A",
            "#DAB36A", "#7FBFF5", "#7FBFF5", "#7FBFF5", "#7FBFF5", "#7FBFF5")
names(faccols) = colnames(humanCD4T_signal_ex$chr7)
dlist=list()
for(n in colnames(humanCD4T_signal_ex$chr7)) {
    dlist[[n]] = DataTrack(data=humanCD4T_signal_ex$chr7[,n], start=humanCD4T_probeAnno_ex$chr7,
                 end = humanCD4T_probeAnno_ex$chr7+200, chromosome="chr7", genome=gen, name=n,
                 type="h", col=faccols[n])
}

myViterbiDirs = list(F=c("F1", "F2"), U=c("U1", "U2"), R=c("R1", "R2"))
myViterbiPanels = list()
cols = rainbow(4)
cols = cols[c(1:2,1:2,3:4)]

for(dir in c("F", "U", "R")) {
    myRle = Rle(humanCD4T_viterbi_collapsed$chr7)
    currItems = which(myRle@values %in% myViterbiDirs[[dir]])

    start = humanCD4T_probeAnno_ex$chr7[start(myRle)][currItems]
    width = myRle@lengths[currItems]
    ids = myRle@values[currItems]
    values = myRle@values[currItems]
    currGRange = GRanges(seqnames=rep("chr7", length(currItems)),ranges=IRanges(start=start,
                         width=width*200, names=values[currItems]))
```

```
    myViterbiPanels[[dir]] = AnnotationTrack(range=currGRange, genome="hg18", chromosome="chr7",
                   name=paste("Viterbi\n", "(", dir, ")", sep=""), id=ids[order(start)],
                   shape="box",fill=cols[values[order(start)]], col="black", stacking="dense")
}

flagpos = humanCD4T_probeAnno_ex$chr7[range(which(humanCD4T_flags_ex$chr7 == "R"))]
flagpanel = list(AnnotationTrack(start=flagpos[1], end=flagpos[2], feature="flags",
                id="flags-1", strand="-", chromosome="chr7", genome="hg18",
                stacking="squish", name="flags", fill="coral", col="black", shape="arrow"))


sizes = rep(1,20)
sizes[16] = 2
sizes[17:20] = 0.7
sizes[1] = 0.5
plotTracks(c(list(humanCD4T_ideogramChr7), list(gtrack), dlist, list(humanCD4T_ucscGenes),
                  flagpanel, myViterbiPanels), from=134600000, to=135350000, sizes=sizes,
                  showFeatureId=TRUE, featureAnnotation="id", fontcolor.feature="black",
                  cex.feature=0.7, background.title="darkgrey")
```

5 Initialization of (bd)HMMs

The EM algorithm needs an initial estimate of the model parameters. This of crucial importance, since the EM algorithm does not find the global maximum of the likelihood, but may converge to a local optimum. In the following we want to give a short example for initialization of (bdHMMs) applied to the yeast data set of this vignette. In case of continuous data, k-means clustering is an easy and practical way to define initial estimates for the means of multivariate gaussian distributions. This partitions the observations into k pre-defined clusters in which each observation belongs to the cluster with the nearest mean by minimizing a cost function which is in our case based on euclidian distance.
K-means clustering is available in R through the `kmeans()` function. We summarize our data into a matrix and create two non-strand-specific expression tracks, where one track contains the maximum value of expression and the other the minumum value of expresssion at each position. This enables clustering the data without partinioning it by the strand-specificity of expression:

```
nStates = 7
myMat = do.call("rbind", yeastTF_databychrom_ex)
myMat = myMat[apply(myMat, 1, function(x) all(! is.na(x))),]
myMat[, c("YPDexprW", "YPDexprC")] = t(apply(myMat[, c("YPDexprW", "YPDexprC")], 1,
                                              sort, decreasing=TRUE))
km = kmeans(myMat, centers=nStates, iter.max=1000, nstart=100)$centers
km[order(km[,"YPDexprW"], decreasing=TRUE),]
##    Nucleosome       Rpb3         S5P        S2P      Tfiib        Ctk1        Paf1
## 5  0.62309988 0.76690243  0.87373061 0.21394292 0.87833458  0.64475359 0.637739168
## 7  0.66355190 0.47853306  0.42201250 0.56886040 0.27404783  0.50723016 0.560427168
## 4  0.77231109 0.47692031  0.58341374 0.12223833 0.66698573  0.29651272 0.332267265
## 3  0.76694982 0.03402459  0.06062472 0.31577506 0.05472344 -0.01107984 0.124515290
## 6  0.57492692 0.24951630  0.22254917 0.38041650 0.20482187  0.31100330 0.313890122
## 2 -0.03857955 0.73854789  0.92266468 0.08515468 1.20545865  0.48888822 0.186747247
## 1  0.52827685 0.01195744 -0.03689882 0.04163995 0.22229223  0.30232825 0.003276345
##          Spt5      Spt16     Pcf11  YPDexprW   YPDexprC
## 5 0.75312450 0.70932772 0.5711418 0.9000490 0.07644764
## 7 0.51054073 0.39419845 0.4514209 0.7433224 0.10337291
```

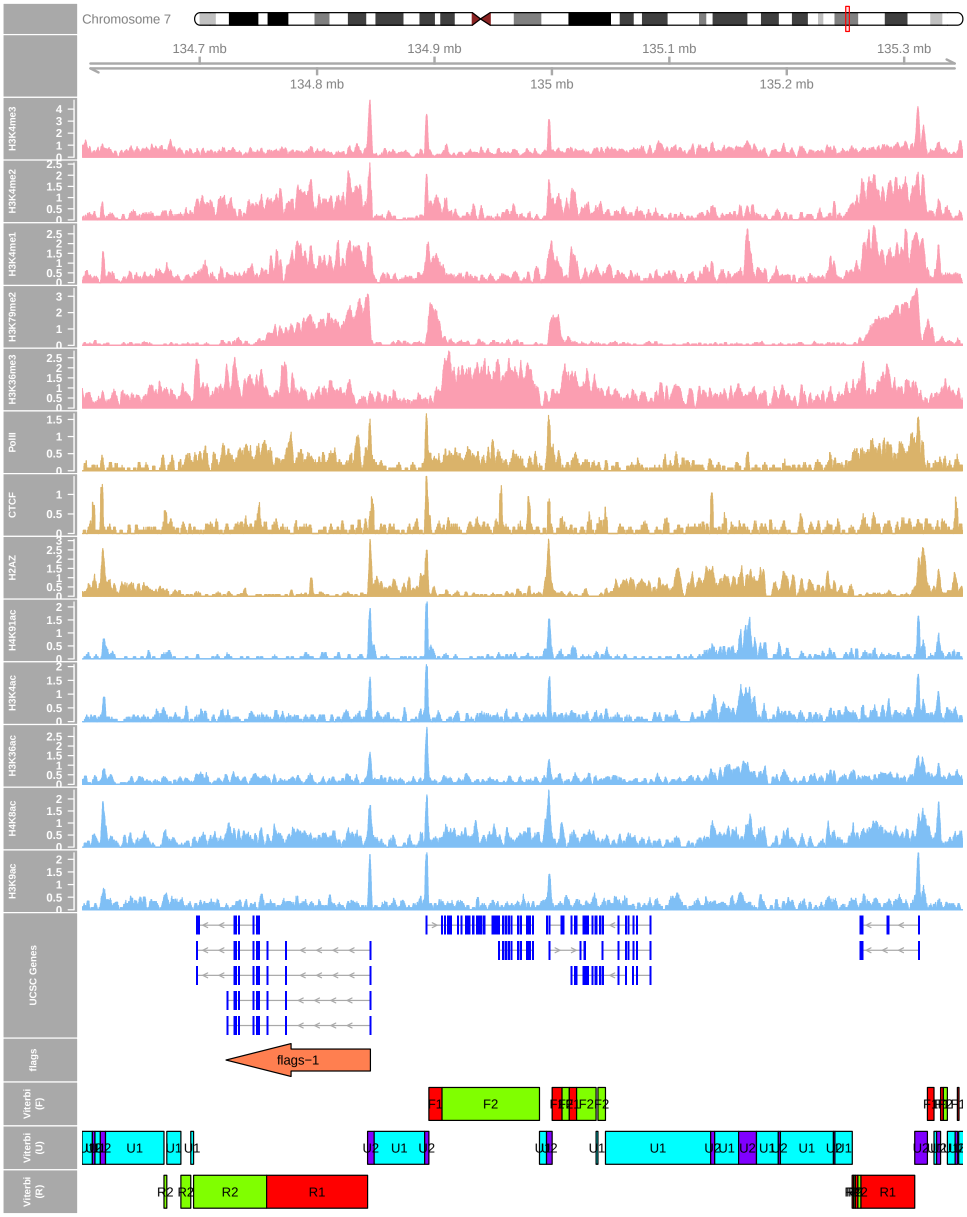

Figure 4: Chromatin modification data, viterbi path and RefSeq gene annotation are shown. Covarage tracks are shown shown for Histone acetylations (blue), methylations (red), PolII, CTCF and H2AZ (brown). The viterbi path was calculated with the 8 state bdHMM, where state F1/R1 and F4/R4 were collapsed into undirected states U1 and U2. Directionality of transcripts is recovered at all four transcribed loci, although only one gene is used for initialization of directionality.

```
## 4 0.43335188 0.35894742 0.3359256 0.7144904 0.08067237
## 3 0.06831644 0.03224004 0.1226538 0.5415321 0.07389047
## 6 0.25643980 0.20402070 0.3516923 0.5024497 0.07803365
## 2 0.52887886 0.44008536 0.4919228 0.1611639 0.06681443
## 1 0.02203180 0.10843890 0.3786942 0.1227376 0.04416329
```

Based on the expression value, we can decide a priori which states are directed or undirected, i.e. clusters with high (max) expression would be directed and clusters with low (max) expression would be undirected.

The cluster means are then used as initial estimates for the means of the multivariate gaussian emissions. The initial covariances can be set to the covariance of the whole data and transitions and initial state probabilities may be initialized uniform. This should give the EM algorithm a good estimate of the state means and provide enough flexibitlity (through the broad covariance and uniform probabilites) to find good parameter estimates.
Please note that this is just an example and that there are many more ways to properly initialize an HMM. Another way is for instance to use multiple runs of random initialization and use the model with the highest likelihood. Moreover, the initialization procedure of course also depends on the type of data, i.e. a continuous model should be initialized differently than a discrete model.

6 Concluding Remarks

This vignette was generated using the following package versions:

- R version 3.1.1 Patched (2014-09-25 r66681), `x86_64-unknown-linux-gnu`
- Locale: `LC_CTYPE=en_US.UTF-8`, `LC_NUMERIC=C`, `LC_TIME=en_US.UTF-8`, `LC_COLLATE=C`, `LC_MONETARY=en_US.UTF-8`, `LC_MESSAGES=en_US.UTF-8`, `LC_PAPER=en_US.UTF-8`, `LC_NAME=C`, `LC_ADDRESS=C`, `LC_TELEPHONE=C`, `LC_MEASUREMENT=en_US.UTF-8`, `LC_IDENTIFICATION=C`
- Base packages: base, datasets, grDevices, graphics, grid, methods, parallel, stats, stats4, utils
- Other packages: BiocGenerics 0.12.0, GenomeInfoDb 1.2.0, GenomicRanges 1.18.0, Gviz 1.10.0, IRanges 2.0.0, Rsolnp 1.14, S4Vectors 0.4.0, STAN 1.0.0, XVector 0.6.0, gplots 2.14.2, knitr 1.7, truncnorm 1.0-7
- Loaded via a namespace (and not attached): AnnotationDbi 1.28.0, BBmisc 1.7, BSgenome 1.34.0, BatchJobs 1.4, Biobase 2.26.0, BiocParallel 1.0.0, BiocStyle 1.4.0, Biostrings 2.34.0, DBI 0.3.1, Formula 1.1-2, GenomicAlignments 1.2.0, GenomicFeatures 1.18.0, Hmisc 3.14-5, KernSmooth 2.23-13, R.methodsS3 1.6.1, RColorBrewer 1.0-5, RCurl 1.95-4.3, RSQLite 0.11.4, Rcpp 0.11.3, Rsamtools 1.18.0, VariantAnnotation 1.12.0, XML 3.98-1.1, acepack 1.3-3.3, base64enc 0.1-2, biomaRt 2.22.0, biovizBase 1.14.0, bitops 1.0-6, brew 1.0-6, caTools 1.17.1, checkmate 1.4, cluster 1.15.3, codetools 0.2-9, colorspace 1.2-4, dichromat 2.0-0, digest 0.6.4, evaluate 0.5.5, fail 1.2, foreach 1.4.2, foreign 0.8-61, formatR 1.0, gdata 2.13.3, gtools 3.4.1, iterators 1.0.7, lattice 0.20-29, latticeExtra 0.6-26, matrixStats 0.10.0, munsell 0.4.2, nnet 7.3-8, plyr 1.8.1, rpart 4.1-8, rtracklayer 1.26.0, scales 0.2.4, sendmailR 1.2-1, splines 3.1.1, stringr 0.6.2, survival 2.37-7, tools 3.1.1, zlibbioc 1.12.0

References

[1] B. Zacher, M. Lidschreiber, P. Cramer, J. Gagneur, and A. Tresch. Annotation of directed genomic states unveils variations in the Pol II transcription cycle. *submitted*, 2014.

[2] J. Ernst and M. Kellis. Discovery and characterization of chromatin states for systematic annotation of the human genome. *Nat. Biotechnol.*, 28(8):817–825, Aug 2010.
