# Supplementary material for: Annotation of genomics data using bidirectional hidden Markov models unveils variations in Pol II transcription cycle
Source: Mol Syst Biol. 2014 Dec 20;10(12):768. doi: 10.15252/msb.20145654 (PMC4300491; doi:10.15252/msb.20145654)
Supplement: Supplementary file 2 [file msb0010-0768-sd2.zip › Stan Software package/STAN-manual.pdf]

Package ‘STAN’

November 18, 2014

Version 1.0.0

Date 2014-07-18

Title STrand-specific ANnotation of genomic data

Author Benedikt Zacher, Julien Gagneur, Achim Tresch

Maintainer Benedikt Zacher <zacher@lmb.uni-muenchen.de>

Depends Rsolnp, methods

VignetteBuilder knitr

Suggests BiocStyle, Gviz, GenomicRanges, IRanges, gplots, knitr

Description STAN (STrand-specic ANnotation of genomic data) implements bidirectional Hidden Markov Models (bdHMM), which are designed for studying directed genomic processes, such as gene transcription, DNA replication, recombination or DNA repair by integrating genomic data. bdHMMs model a sequence of successive observations (e.g. ChIP or RNA measurements along the genome) by a discrete number of 'directed genomic states', which e.g. reflect distinct genome-associated complexes. Unlike standard HMM approaches, bdHMMs allow the integration of strand-specific (e.g. RNA) and non strand-specific data (e.g. ChIP).

License GPL (>= 2)

biocViews HiddenMarkovModel, GenomeAnnotation, Microarray, Sequencing

LazyLoad yes

R topics documented:

STAN-package 2
bdHMM 2
bdHMM-class 3
bdhmm_ex 4
example 5
fitHMM 5
flags 6
getPosterior 6

getViterbi . . . 7
HMM . . . 8
HMM-class . . . 8
HMMEmission . . . 9
HMMEmission-class . . . 9
hmm_ex . . . 10
humanCD4T_flags_ex . . . 10
humanCD4T_ideogramChr7 . . . 10
humanCD4T_initCovs . . . 11
humanCD4T_initMeans . . . 11
humanCD4T_probeAnno_ex . . . 11
humanCD4T_signal_ex . . . 11
humanCD4T_ucscGenes . . . 12
observations . . . 12
yeastTF_databychrom_ex . . . 12
yeastTF_initCovs . . . 12
yeastTF_initMeans . . . 13
yeastTF_probeAnno_ex . . . 13
yeastTF_SGDGenes . . . 13

Index **14**

`STAN-package` *STrand-specific ANnotation of genomic data*

Description

STrand-specific ANnotation of genomic data

Author(s)

Benedikt Zacher, Julien Gagneur, Achim Tresch

References

B. Zacher, M. Lidschreiber, P. Cramer, J. Gagneur, and A. Tresch. Annotation of directed genomic states unveils variations in the Pol II transcription cycle. submitted, 2014.

`bdHMM` *Create a bdHMM object*

Description

This function creates a bdHMM function.

Usage

```
bdHMM(initProb = numeric(), transMat = matrix(numeric(), ncol = 1, nrow =
  1), emission, nStates = numeric(), status = character(),
  stateLabel = character(), transitionsOptim = character(),
  directedObs = integer())
```

Arguments

- `initProb` Initial state probabilities.
- `transMat` Transition probabilities
- `emission` Emission parameters as an HMMEmission object.
- `nStates` Number of states.
- `stateLabel` Indicates directinality of states. States can be forward (F1, F2, ..., Fn), reverse (R1, R2, ..., Rn) or undirectional (U1, U2, ..., Um). Number of F and R states must be equal and twin states are indicated by integers in id (e.g. F1 and R1 and twins).
- `transitionsOptim` There are three methods to choose from for fitting the transitions. Bidirectional transition matrices (invariant under reversal of time and direction) can be fitted using c('rsolnp', 'ipopt'). 'None' uses standard update formulas and the resulting matrix is not constrained to be bidirectional.
- `directedObs` An integer indicating which dimensions are directed. Undirected dimensions are 0. Directed observations must be marked as unique integer pairs. For instance c(0,0,0,0,0,1,1,2,2,3,3) contains 5 undirected observations, and thre pairs (one for each direction) of directed observations.
- `status` Status of the bdHMM. 'Initial' means that the model was not fitted yet. 'EM' means that the model was optimized using Expectation maximization.

See Also

HMMEmission

Examples

```
nStates = 5
stateLabel = c(F1, F2, R1, R2, U1)
means = list(4,11,4,11,-1)
Sigma = lapply(list(4,4,4,4,4), as.matrix)
transMat = matrix(1/nStates, nrow=nStates, ncol=nStates)
initProb = rep(1/nStates, nStates)

bdHMM(initProb=initProb, transMat=transMat, emission=HMMEmission(type=Gaussian, parameters=list(mean=means
```

`bdHMM-class` *This class is a generic container for bidirectional Hidden Markov Models.*

Description

This class is a generic container for bidirectional Hidden Markov Models.

Slots

`initProb` Initial state probabilities.

`transMat` Transition probabilities

`emission` Emission parameters as an HMMEmission object.

`nStates` Number of states.

`stateLabel` Indicates directinality of states. States can be forward (F1, F2, ..., Fn), reverse (R1, R2, ..., Rn) or undirectional (U1, U2, ..., Um). Number of F and R states must be equal and twin states are indicated by integers in id (e.g. F1 and R1 and twins).

`transitionsOptim` There are three methods to choose from for fitting the transitions. Bidirectional transition matrices (invariant under reversal of time and direction) can be fitted using c('rsolnp', 'ipopt'). 'None' uses standard update formulas and the resulting matrix is not constrained to be bidirectional.

`directedObs` An integer indicating which dimensions are directed. Undirected dimensions are 0. Directed observations must be marked as unique integer pairs. For instance c(0,0,0,0,0,1,1,2,2,3,3) contains 5 undirected observations, and thre pairs (one for each direction) of directed observations.

`status` Status of the bdHMM. 'Initial' means that the model was not fitted yet. 'EM' means that the model was optimized using Expectation maximization.

See Also

HMMEmission

Examples

```
nStates = 5
stateLabel = c(F1, F2, R1, R2, U1)
means = list(4,11,4,11,-1)
Sigma = lapply(list(4,4,4,4,4), as.matrix)
transMat = matrix(1/nStates, nrow=nStates, ncol=nStates)
initProb = rep(1/nStates, nStates)

bdHMM(initProb=initProb, transMat=transMat, emission=HMMEmission(type=Gaussian, parameters=list(mean=means
```

`bdhmm_ex` *Initial bdHMM for the Quick-Start example in the vignette*

Description

Initial bdHMM for the Quick-Start example in the vignette

Author(s)

Benedikt Zacher, Julien Gagneur, Achim Tresch

example — *The data for the Quick-Start example in the vignette*

Description

The data for the Quick-Start example in the vignette

Author(s)

Benedikt Zacher, Julien Gagneur, Achim Tresch

fitHMM — *Fit a Hidden Markov Model*

Description

The function is used to fit (bidirectional) Hidden Markov Models, given one or more observation sequence.

Usage

```
fitHMM(obs=list(), hmm, convergence=1e-06, maxIters=1000, dirFlags=list(), emissionProbs=list(),
```

Arguments

`obs`	The observations. A list of one or more entries containing the observation matrix (`numeric`) for the samples (e.g. chromosomes).
`hmm`	The initial Hidden Markov Model. This is a `HMM`.
`convergence`	Convergence cutoff for EM-algorithm (default: 1e-6).
`maxIters`	Maximum number of iterations.
`dirFlags`	The flag sequence is needed when a bdHMM is fitted on undirected data (e.g.) ChIP only. It is a `list` of `character` vectors indication for each position its knwon directionality. U allows all states. F allows undirected states and states in forward direction. R allows undirected states and states in reverse direction.
`emissionProbs`	List of precalculated emission probabilities of emission function is of type 'null'.
`effectiveZero`	Transitions below this cutoff are analytically set to 0 to speed up comptuations.
`verbose`	`logical` for printing algorithm status or not.
`nCores`	Number of cores to use for computations.
`incrementalEM`	When TRUE, the incremental EM is used to fit the model, where parameters are updated after each iteration over a single observation sequence.
`observationEmissionType`	Only needed when HMMEmission is 'JointlyIndependent'. Defines for each dimension (columns in obs) of the data the type of emission to be used.

Value

A list containing the trace of the log-likelihood during EM learning and the fitted HMM model.

See Also

HMM

Examples

```
data(example)
hmm_fitted = fitHMM(observations, hmm_ex)
```

---

flags *Pre-computed flag sequence for the Quick-Start example in the vignette*

Description

Pre-computed flag sequence for the Quick-Start example in the vignette

Author(s)

Benedikt Zacher, Julien Gagneur, Achim Tresch

---

getPosterior *Calculate posterior state distribution.*

Description

The function calculates posterior state probabilities for one or more observation sequence.

Usage

```
getPosterior(hmm, obs=list(), emissionProbs=list(), dirFlags=list(), verbose=FALSE, nCores=1)
```

Arguments

`hmm`	The initial Hidden Markov Model. This is a `HMM`.
`obs`	The observations. A list of one or more entries containing the observation matrix (`numeric`) for the samples (e.g. chromosomes).
`emissionProbs`	List of precalculated emission probabilities of emission function is of type 'null'.
`dirFlags`	The flag sequence is needed when a bdHMM is fitted on undirected data (e.g.) ChIP only. It is a `list` of `character` vectors indication for each position its knwon directionality. U allows all states. F allows undirected states and states in forward direction. R allows undirected states and states in reverse direction.
`verbose`	`logical` for printing algorithm status or not.
`nCores`	Number of cores to use for computations.

Value

A list containing for the observation sequences the posterior state (col) distribution at each position (row).

See Also

HMM

Examples

```
data(example)
hmm_fitted = fitHMM(observations, hmm_ex)
posterior_hmm = getPosterior(hmm_fitted$hmm, observations)
```

---

getViterbi *Calculate the most likely state path*

Description

Given a Hidden Markov Model, the function calculates the most likely state path (viterbi) for one or more observation sequence.

Usage

```
getViterbi(HMM, obs=list(), NAtol=5, emissionProbs=list(), verbose=FALSE)
```

Arguments

HMM	The initial Hidden Markov Model. This is a HMM.
obs	The observations. A list of one or more entries containing the observation matrix (numeric) for the samples (e.g. chromosomes).
NAtol	Successive positions having NAs longer than this threshold are masked in the viterbi path.
emissionProbs	List of precalculated emission probabilities of emission function is of type 'null'.
verbose	logical for printing algorithm status or not.

Value

A list containint the vterbi paths.

See Also

HMM

Examples

```
data(example)
hmm_fitted = fitHMM(observations, hmm_ex)
viterbi_hmm = getViterbi(hmm_fitted$hmm, observations)
```

HMM *Create a HMM object*

Description

This function creates a HMM object.

Usage

```
HMM(initProb = numeric(), transMat = matrix(numeric(), ncol = 1, nrow = 1),
  emission, nStates = numeric(), status = character())
```

Arguments

initProb	Initial state probabilities.
transMat	Transition probabilities
emission	Emission parameters as an HMMEmission object.
nStates	Number of states.
status	of the HMM. On of c('initial', 'EM').

See Also

HMMEmission

Examples

```
nStates = 5
means = list(4,11,4,11,-1)
Sigma = lapply(list(4,4,4,4,4), as.matrix)
transMat = matrix(1/nStates, nrow=nStates, ncol=nStates)
initProb = rep(1/nStates, nStates)
HMM(initProb=initProb, transMat=transMat, emission=HMMEmission(type=Gaussian, parameters=list(mean=means, c
```

HMM-class *This class is a generic container for Hidden Markov Models.*

Description

This class is a generic container for Hidden Markov Models.

Slots

- initProb Initial state probabilities.
- transMat Transition probabilities
- emission Emission parameters as an HMMEmission object.
- nStates Number of states.
- status of the HMM. On of c('initial', 'EM').

See Also

HMMEmission

Examples

```
nStates = 5
means = list(4,11,4,11,-1)
Sigma = lapply(list(4,4,4,4,4), as.matrix)
transMat = matrix(1/nStates, nrow=nStates, ncol=nStates)
initProb = rep(1/nStates, nStates)
HMM(initProb=initProb, transMat=transMat, emission=HMMEmission(type=Gaussian, parameters=list(mean=means, c
```

HMMEmission *Create a HMMEmission object*

Description

This function creates a HMMEmission object.

Usage

```
HMMEmission(type = character(), parameters = list(), nStates = integer())
```

Arguments

type	The type of emission function c('Gaussian').
parameters	A list containing the the parameters for each state.
nStates	The number of states.

Examples

```
nStates = 5
means = list(4,11,4,11,-1)
Sigma = lapply(list(4,4,4,4,4), as.matrix)
transMat = matrix(1/nStates, nrow=nStates, ncol=nStates)
initProb = rep(1/nStates, nStates)
HMMEmission(type=Gaussian, parameters=list(mean=means, cov=Sigma), nStates=length(means))
```

HMMEmission-class *This class is a generic container for different emission functions of Hidden Markov Models.*

Description

This class is a generic container for different emission functions of Hidden Markov Models.

Slots

`type` The type of emission function c('Gaussian').

`parameters` A list containing the the parameters for each state.

`dim` Number of dimensions.

`nStates` The number of states.

Examples

```
nStates = 5
means = list(4,11,4,11,-1)
Sigma = lapply(list(4,4,4,4,4), as.matrix)
transMat = matrix(1/nStates, nrow=nStates, ncol=nStates)
initProb = rep(1/nStates, nStates)
HMMEmission(type=Gaussian, parameters=list(mean=means, cov=Sigma), nStates=length(means))
```

`hmm_ex` *Initial HMM for the Quick-Start example in the vignette*

Description

Initial HMM for the Quick-Start example in the vignette

Author(s)

Benedikt Zacher, Julien Gagneur, Achim Tresch

`humanCD4T_flags_ex` *Pre-defined flag sequence for the human CD4T-cell example*

Description

Pre-defined flag sequence for the human CD4T-cell example

Author(s)

Benedikt Zacher, Julien Gagneur, Achim Tresch

`humanCD4T_ideogramChr7` *Ideogram track of human chromosome 7 for the human CD4T-cell example*

Description

Ideogram track of human chromosome 7 for the human CD4T-cell example

Author(s)

Benedikt Zacher, Julien Gagneur, Achim Tresch

humanCD4T_initCovs — *Pre-computed initial estimates of the covairances for the human CD4T-cell example*

Description

Pre-computed initial estimates of the covairances for the human CD4T-cell example

Author(s)

Benedikt Zacher, Julien Gagneur, Achim Tresch

humanCD4T_initMeans — *Pre-computed initial estimates of the means for the human CD4T-cell example*

Description

Pre-computed initial estimates of the means for the human CD4T-cell example

Author(s)

Benedikt Zacher, Julien Gagneur, Achim Tresch

humanCD4T_probeAnno_ex — *Genomic positions of processed signal for the human CD4T-cell example*

Description

Genomic positions of processed signal for the human CD4T-cell example

Author(s)

Benedikt Zacher, Julien Gagneur, Achim Tresch

humanCD4T_signal_ex — *Processed ChIP-Seq signal for the human CD4T-cell example*

Description

Processed ChIP-Seq signal for the human CD4T-cell example

Author(s)

Benedikt Zacher, Julien Gagneur, Achim Tresch

humanCD4T_ucscGenes — *UCSC gene annotation for the human CD4T-cell example*

Description

UCSC gene annotation for the human CD4T-cell example

Author(s)

Benedikt Zacher, Julien Gagneur, Achim Tresch

observations — *Observation sequence of the Quick-Start example in the vignette*

Description

Observation sequence of the Quick-Start example in the vignette

Author(s)

Benedikt Zacher, Julien Gagneur, Achim Tresch

yeastTF_databychrom_ex — *Processed ChIP-on-chip data for yeast TF example*

Description

Processed ChIP-on-chip data for yeast TF example

Author(s)

Benedikt Zacher, Julien Gagneur, Achim Tresch

yeastTF_initCovs — *Pre-computed initial estimates of the covairances for yeast TF example*

Description

Pre-computed initial estimates of the covairances for yeast TF example

Author(s)

Benedikt Zacher, Julien Gagneur, Achim Tresch

yeastTF_initMeans *Pre-computed initial estimates of the means for yeast TF example*

Description

Pre-computed initial estimates of the means for yeast TF example

Author(s)

Benedikt Zacher, Julien Gagneur, Achim Tresch

yeastTF_probeAnno_ex *Genomic positions of the ChIP-on-chip data for yeast TF example*

Description

Genomic positions of the ChIP-on-chip data for yeast TF example

Author(s)

Benedikt Zacher, Julien Gagneur, Achim Tresch

yeastTF_SGDGenes *SGD annotation for the yeast TF example*

Description

SGD annotation for the yeast TF example

Author(s)

Benedikt Zacher, Julien Gagneur, Achim Tresch

Index

*Topic **data**
bdhmm_ex, 4
example, 5
flags, 6
hmm_ex, 10
humanCD4T_flags_ex, 10
humanCD4T_ideogramChr7, 10
humanCD4T_initCovs, 11
humanCD4T_initMeans, 11
humanCD4T_probeAnno_ex, 11
humanCD4T_signal_ex, 11
humanCD4T_ucscGenes, 12
observations, 12
yeastTF_databychrom_ex, 12
yeastTF_initCovs, 12
yeastTF_initMeans, 13
yeastTF_probeAnno_ex, 13
yeastTF_SGDGenes, 13
*Topic **package**
STAN-package, 2
.HMM *(*HMM-class*)*, 8
.HMMEmission *(*HMMEmission-class*)*, 9
.bdHMM *(*bdHMM-class*)*, 3

bdHMM, 2
bdHMM-class, 3
bdhmm_ex, 4

example, 5

fitHMM, 5
flags, 6

getPosterior, 6
getViterbi, 7

HMM, *5–7*, 8
HMM-class, 8
hmm_ex, 10
HMMEmission, *3*, *4*, *8*, *9*, 9
HMMEmission-class, 9
humanCD4T_flags_ex, 10
humanCD4T_ideogramChr7, 10
humanCD4T_initCovs, 11
humanCD4T_initMeans, 11
humanCD4T_probeAnno_ex, 11
humanCD4T_signal_ex, 11
humanCD4T_ucscGenes, 12

observations, 12

STAN-package, 2

yeastTF_databychrom_ex, 12
yeastTF_initCovs, 12
yeastTF_initMeans, 13
yeastTF_probeAnno_ex, 13
yeastTF_SGDGenes, 13
