# Supplementary material for: Annotation of genomics data using bidirectional hidden Markov models unveils variations in Pol II transcription cycle
Source: Mol Syst Biol. 2014 Dec 20;10(12):768. doi: 10.15252/msb.20145654 (PMC4300491; doi:10.15252/msb.20145654)
Supplement: Supplementary file 3 [file msb0010-0768-sd3.pdf]

Annotation of genomics data using bidirectional hidden Markov models unveils variations in Pol II transcription cycle

Benedikt Zacher, Michael Lidschreiber, Patrick Cramer, Julien Gagneur, and Achim Tresch

Corresponding author: Achim Tresch, Max Planck Institute for Plant Breeding Research

Review timeline:

Submission date:	04 August 2014
Editorial Decision:	26 September 2014
Revision received:	24 October 2014
Editorial Decision:	17 November 2014
Revision received:	19 November 2014
Accepted:	21 November 2014

Editor: Maria Polychronidou

Transaction Report:

(Note: With the exception of the correction of typographical or spelling errors that could be a source of ambiguity, letters and reports are not edited. The original formatting of letters and referee reports may not be reflected in this compilation.)

1st Editorial Decision 26 September 2014

Thank you again for submitting your work to Molecular Systems Biology. We have now heard back from two of the three referees who agreed to evaluate your manuscript. Unfortunately, after several reminders we have still not received a report from reviewer #2. Since the recommendations of reviewers #1 and #3 are similar, I prefer to make a decision now, rather than further delaying the process. As you will see from the reports below, the referees acknowledge that the presented approach is potentially interesting. However, they raise a series of concerns and make suggestions for modifications which we would ask you to carefully address in a revision of the manuscript. The referees' recommendations are clear in this regard.

On a more editorial level, we would like to mention that while we generally encourage the submission of individual Supplementary Figure/Table files, in exceptional cases (and depending on the nature of information provided) we allow the use of a single PDF file including a Table of Contents. We think that in this case, it would be better to present the Supplementary Information in the single PDF file format.

Thank you for submitting this paper to Molecular Systems Biology.

Reviewer #1:

In this manuscript, Zacher et al. introduce the concept of bidirectional hidden Markov models

(bdHMMs) for inferring the sequence of states that a transcriptional complex (for example) passes through as it goes from the promoter to the transcription termination site. A bdHMM is a special type of HMM that allows identification of pairs of equivalent states that differ only in their directionality. This is an excellent idea, because the molecular processes occurring on the forward and reverse strands of chromosomes are identical, and differ only in direction. It makes much more sense to model the genome using bdHMMs. Conventional HMMs ignore directionality and therefore necessarily have unrealistic state transition probabilities.

The authors go to impressive lengths to construct the mathematical framework of bdHMMs, which is not at all trivial. I would consider this a major conceptual advance. They also provide their method as a downloadable software package with acceptable running time. They apply the method to the yeast genome and also the human genome, with a focus on annotating the molecular transitions observed at transcribed genes. In doing so, they uncover a very interesting and fundamental diversity of gene types. Specifically, they find that promoter escape and transcriptional elongation are regulated very differently at genes from different classes. They also find that nucleosome depletion, which was previously thought to be a feature of transcription termination sites in yeast, may actually only mark termination sites that coincide with the promoter of another gene. Overall, the manuscript describes a timely new method that can be used on a number of different genomic profiling datasets, and also shed new light on basic molecular mechanisms of transcription.

Major comments

If nucleosome occupancy has been profiled in CD4+ T cells (and I think it has), it would be very useful confirm that the conclusions from yeast transcription termination site analysis are also valid in human.

The comparison to conventional HMM modeling is good because it shows how transcriptional directionality can be inferred by the bdHMM model. However, it was limited to state transition probabilities because emission probabilities were kept fixed. To convince the research community to use this method, it would be good to allow emission probabilities to differ - that is how the programs will actually be run by users. Also, it would be important to see a comparison of annotation accuracy: does the bdHMM approach produce more accurate genome annotations than conventional HMMs?

Minor comments

I completely agree that there is no point in trying to use information-theoretic criteria (BIC, AIC, etc.) to estimate the "true" number of HMM states. Information-theoretic approaches typically overestimate the number of states because of over-fitting to technical artifacts. Trial-and-error is the only reasonable option.

Page 2, second paragraph: "Metagene analysis, in spite of ... this hypothesis." It's not clear which hypothesis this sentence refers to.

After reading the abstract and the first two paragraphs of the Background, I did not get a clear idea of which exact problem this paper solves. This became clearer only after I was well into the manuscript. Perhaps the introductory portions could be reworded for clarity.

Page 3: " The hidden variables form a Markov chain, which means that the probability for observing st
depends only on st 1, the transition probability Pr(st | st 1)." This is true only for first-order Markov chains.

Same paragraph as above: it may be better to use posterior probabilities of states for genome annotation, rather than using the Viterbi path. Perhaps the authors could comment on this point? Are they using the Viterbi path because annotating genomic bins by their posterior probabilities could cause the inferred state to flip back and forth in an unstable manner?

End of Page 9: it's not exactly clear what is meant by binary data. Is this a reference to discretization

of the ChIP-seq profiles into present/absent within each bin?

End of Page 10: what is the rationale behind the first two symmetry conditions? These look more appropriate for describing equilibrium conditions than the initial state. My understanding is that the initial state distribution reflects the probabilities of the various states just before "entering" the beginning of a chromosomal arm. It would be good to see some explanation of why these initial state probabilities should obey detailed balance or the initiation symmetry condition.

Figure 5c: is the novel CUT simply an extension of CUT007? Is CUT007 already known?

Figure 5d: Does the SUT not have a well-defined termination site? Is it polyadenylated? Do the authors have a guess for why it was not discovered earlier?

Reviewer #3:

Review of Zacher et al "Annotation of genomics data by the bidirectional hidden Markov model variations in Pol II transcription cycle"

Summary

The authors introduce a variant of HMMs suitable for genomic annotation, where some states are directional, but can appear in either "+" or "-" strand. They show how to apply their method to annotate high-dimensional genomic data (RNA, ChIP, etc) to find the footprint of processes taking place on the genome.

Discussion

I like the approach. There is a nice straightforward thinking about the issues involved in genome annotations for directed processes (e.g., transcription). The authors present a useful and lucid analysis of the yeast transcription cycle that highlights locations that deviate from the "canonical" sequence of events described in the biochemical literature, as well identify events that were missed by direct observations.

I have some technical issues with the presentation of the methods and a comment that I believe will simplify that section (see below). I also would appreciate more insights into the biology uncovered by the analysis. This will substantially strengthen the confidence in this approach and its usefulness. See detailed comments below.

With these caveats in mind, and believing that the authors can address them rather easily, I recommend acceptance of this paper to MSB.

Comments

- I would appreciate some more insight on the results of the analysis. What are the genes that fall into different clusters? do we have a reason to believe they are co-regulated (e.g., I bet that ribosomal protein genes appear as one cluster)? and if so, does the specific sequence state (and their associated observations) match the known biology on these regulatory strategies (e.g., SAGA-dependent promoters vs TFIID-dependent promoters)? I understand that some answers appear in the supplement (I did not read it). I believe that they should be made more pronounced in the main text.

- As far as I can tell, the authors do not discuss how they initialized the model (number of directional/undirectional states) or how many random initialization points where used. They discuss this in the context of the simulation, but it is unclear whether the same procedure was used on the genomic data. It seems that this requires non-trivial manual tuning to get good results (which is worrisome).

A useful initialization can be based on standard HMM learning. Learn a model, and then introduce directional states (i,j, k) to represent state j appearing after i and before k. This defines an obvious conjunction function -(i,j,k) = (k,i,j) and provides a nice initialization for the model. Of course, this

initialization comes at the price of cubic number of states, but these can be easily trimmed down (removing rare triplets) or simplified by state merging.

- Regarding the initialization, in that simulation section, the authors initialize from a slightly perturbed uniform distribution. While this initialization escapes the saddle-point defined by the uniform distribution, it might not be enough to explore solutions that are further from it.

- I would appreciate simulations also from the model learned from the genome. That is, take the model learned (from yeast for example), generate data from it, and then try to learn from it. This simulation will give some intuition how hard it is learn a model such as the one you learned, and will give you confidence estimate in the parameters (the procedure is essentially parametric jackknife).

Technical comment:

The authors go into length in presentation of the EM convergence analysis and discussion of "approximate" version. I believe the following will simplify the presentation and prove the correctness of this approximation.

First, note that while EM with constraints is generally hard, equality constraint are easy (\theta_i = \theta_j) since we can write the likelihood (or Q) in terms of reduced parameter set that do not contain redundancies.

Second, we can reparameterize a bdHMM with the following parameters:

\theta_{i,j} = a_{i,j} / \pi_i

and the vector \pi.

To see this, not that whenever a_{i,j} appears in the likelihood, simply replace with a_{i,j} * \pi_i.

Given the constraints (2) & (3), we can formulate the constrains on \theta_{i,j}

(*) \theta_{i,j} = \theta_{-j,-i}

(**) \pi_i = \sum_j \theta_{i,j}

It is also easy to verify that if \theta and \pi satisfy (*) & (**), then a and \pi satisfy (2) & (3).

Moreover a is a stochastic if and only if

(***) \sum_{i} \pi_i = 1

Thus we are left with three equality constraints (*), (3), and (4) and two sum constraints (**) and (***).

From this point it is easy to check that \Q in terms of the new parameters is equal to ~Q of (eq 23) and that the maximum of this function is as given in eq (27).

Thus, the authors are performing exact EM, where the internal optimization step has a unique solution (i.e., is convex).

1st Revision - authors' response 24 October 2014

We thank the referees for their thorough and generally positive review. We are confident that we could address their concerns, and we changed the manuscript accordingly. Please find below our point-to-point responses to the reviewers. We are looking forward to your decision.

Sincerely,

Achim Tresch

Editorial comments:

• *We think that in this case, it would be better to present the Supplementary Information in the single PDF file format.*

The Supplementary Information has been converted into one single PDF file.

• *We would also like to ask you to provide: A .doc file for the main manuscript text.*

Since our manuscript contains many mathematical formulas, we found it more appropriate to use the MSB TEX template for the revised manuscript. We provide the TEX file and a pdf file generated from it.

• *A "standfirst text" summarizing the study in one or two sentences (approximately 250 characters), three to four "bullet points" highlighting the main findings and a "thumbnail image" (211x157 pixels, jpeg format) to highlight the paper on our homepage. More information is available at: http://msb.embopress.org/authorguide#a4.4>.*

The 250 character summary and three bullet points have been included in the header of the revised manuscript. The thumbnail image has been uploaded as a part of the resubmission. We further went through the MSB checklist and included it in the completed submission form.

Reviewer #1:

• *In this manuscript, Zacher et al. introduce the concept of bidirectional hidden Markov models (bdHMMs) for inferring the sequence of states that a transcriptional complex (for example) passes through as it goes from the promoter to the transcription termination site. A bdHMM is a special type of HMM that allows identification of pairs of equivalent states that differ only in their directionality. This is an excellent idea, because the molecular processes occurring on the forward and reverse strands of chromosomes are identical, and differ only in direction. It makes much more sense to model the genome using bdHMMs. Conventional HMMs ignore directionality and therefore necessarily have unrealistic state transition probabilities.*

The authors go to impressive lengths to construct the mathematical framework of bdHMMs, which is not at all trivial. I would consider this a major conceptual advance. They also provide their method as a downloadable software package with acceptable running time. They apply the method to the yeast genome and also the human genome, with a focus on annotating the molecular transitions observed at transcribed genes. In doing so, they uncover a very interesting and fundamental diversity of gene types. Specifically, they find that promoter escape and transcriptional elongation are regulated very

differently at genes from different classes. They also find that nucleosome depletion, which was previously thought to be a feature of transcription termination sites in yeast, may actually only mark termination sites that coincide with the promoter of another gene. Overall, the manuscript describes a timely new method that can be used on a number of different genomic profiling datasets, and also shed new light on basic molecular mechanisms of transcription.

We thank the reviewer for his/her appreciation of the mathematical idea behind bdHMMs and its utility for genomics.

Major comments:

- *If nucleosome occupancy has been profiled in CD4+ T cells (and I think it has), it would be very useful confirm that the conclusions from yeast transcription termination site analysis are also valid in human.*

In our study, the analysis of the human dataset is performed to compare bdHMM against HMM. To this end, we have used the exact same data and data representation (binary discretization) as in the original study by Ernst and Kellis. A thorough analysis of termination in human is certainly interesting. However, this would require the analysis of an additional data set, including nucleosome data and promoter-associated transcription factors in human (similar to the yeast data we used here). We also anticipate that modeling assumptions, including the binarization, should be reconsidered. Altogether, we think that the inclusion of another data set and its analysis goes beyond the scope of the manuscript.

- *The comparison to conventional HMM modeling is good because it shows how transcriptional directionality can be inferred by the bdHMM model. However, it was limited to state transition probabilities because emission probabilities were kept fixed. To convince the research community to use this method, it would be good to allow emission probabilities to differ - that is how the programs will actually be run by users.*

The purpose of fixing all but the transition parameters was to illustrate the conceptual difference between HMMs and bdHMMs that arises from the bdHMM transition constraints only. To compare of bdHMM and HMM without any parameter fixing, we have now included a quantitative evaluation of its performance for gene boundary prediction on the yeast data set showing that bdHMM outperforms HMM in terms of accuracy of genome annotation (see below for details).

- *Also, it would be important to see a comparison of annotation accuracy: does the bdHMM approach produce more accurate genome annotations than conventional HMMs?*

We thank the reviewer for this suggestion. We now compare the accuracy of transcription start site and polyadenylation site annotation of HMMs and bdHMMs on the yeast data set. bdHMMs substantially improve TSS annotation, whereas pA site annotation is virtually unchanged. This analysis clearly confirms the superiority of bdHMM over HMM for genomics data. We added a new Figure 3D and discuss these results in section “bdHMM state annotation recovers annotated genomic features with high accuracy” of the main text.

Minor comments

- *Page 2, second paragraph: "Metagene analysis, in spite of ... this hypothesis." It's not clear which hypothesis this sentence refers to.*

We restructured the introduction and removed this sentence.

• *After reading the abstract and the first two paragraphs of the Background, I did not get a clear idea of which exact problem this paper solves. This became clearer only after I was well into the manuscript. Perhaps the introductory portions could be reworded for clarity.*

The Introduction/Background section was re-written in order to present it more clearly.

• *Page 3: " The hidden variables form a Markov chain, which means that the probability for observing* s_t *depends only on* s_{t-1}*, the transition probability* $Pr(s_t \mid s_{t-1})$*." This is true only for first-order Markov chains.*

True, although many authors tacitly refer to first order Markov chains when they use the term 'Markov chain'. We made this sentence more precise by saying that these variables form a first order Markov chain.

• *Same paragraph as above: it may be better to use posterior probabilities of states for genome annotation, rather than using the Viterbi path. Perhaps the authors could comment on this point? Are they using the Viterbi path because annotating genomic bins by their posterior probabilities could cause the inferred state to flip back and forth in an unstable manner?*

Thanks, we added a comment in section "Genomic state annotation results in a global, strand-specific transcription map". It is true that Viterbi path is less subject to state flipping in theory. However, we did not see any relevant difference in the state annotation of our data (97% of genomic positions are annotated with the same state when comparing Viterbi and posterior decoded state paths). Posteriors are useful in other applications, an example being our directionality score. The STAN package provides both Viterbi and posterior decoding.

• *End of Page 9: it's not exactly clear what is meant by binary data. Is this a reference to discretization of the ChIP-seq profiles into present/absent within each bin?*

Correct, we use the binarization provided in the ChromHMM paper. We made this clearer in the text and put an additional reference to the original ChromHMM paper by Ernst and Kellis, Nat. Methods (2012).

• *End of Page 10: what is the rationale behind the first two symmetry conditions? These look more appropriate for describing equilibrium conditions than the initial state. My understanding is that the initial state distribution reflects the probabilities of the various states just before "entering" the beginning of a chromosomal arm. It would be good to see some explanation of why these initial state probabilities should obey detailed balance or the initiation symmetry condition.*

Thanks for pointing this out. The two first symmetry conditions are a consequence that at any position of the Markov chain, observing state i followed by state j has the same probability as observing the respective conjugate states, \bar{i} and \bar{j}, in reversed order It might be surprising that the initial probabilities are constrained to match the steady-state probabilities. This is however fine on practical applications for two reasons. First, complex regions (unassembled regions, repeat regions, telomeres, centromeres, etc.) leads to frequent large stretches of missing values. Hence, the model is not run on complete chromosomes, but on the remaining stretches with enough data. For these stretches, the biology of the first base differ. Hence, taking the steady-state probability as initial probability is a reasonable modeling assumption. Second, the stretches are typically long enough so that the influence

of the initial probability is minor on the genomic state annotation. The “Semantic of bdHMMs” paragraph has been extended to explain this point.

- *Figure 5c: is the novel CUT simply an extension of CUT007? Is CUT007 already known?*

We have now realized that a large part of this CUT is actually the protein coding gene FUS3, which is expressed in haploid cells. The transcription data on which the transcriptome annotation is based (YPD, Xu et al. 2009) stems from diploid cells, whereas, all remaining data are from haploid cells.. No, there is a short region between transcripts showing no expression. CUT007 is already known. We changed the Figure to avoid confusion.

- *Figure 5d: Does the SUT not have a well-defined termination site? Is it polyadenylated? Do the authors have a guess for why it was not discovered earlier?*

The SUT is polyadenylated and shows detectable expression, but at a too low level for Xu et al. (2009) criteria. The termination site of this SUT is not very well defined.

Reviewer #3:

- *The authors introduce a variant of HMMs suitable for genomic annotation, where some states are directional, but can appear in either "+" or "-" strand. They show how to apply their method to annotate high-dimensional genomic data (RNA, ChIP, etc) to find the footprint of processes taking place on the genome.*
I like the approach. There is a nice straightforward thinking about the issues involved in genome annotations for directed processes (e.g., transcription). The authors present a useful and lucid analysis of the yeast transcription cycle that highlights locations that deviate from the "canonical" sequence of events described in the biochemical literature, as well identify events that were missed by direct observations. I have some technical issues with the presentation of the methods and a comment that I believe will simplify that section (see below). I also would appreciate more insights into the biology uncovered by the analysis. This will substantially strengthen the confidence in this approach and its usefulness. See detailed comments below.

We thank the Reviewer 3 for carefully studying the mathematical details of the bdHMM and the thoughtful suggestions on their learning, which substantially improved the presentation of the model.

- *I would appreciate some more insight on the results of the analysis. What are the genes that fall into different clusters? Do we have a reason to believe they are co-regulated (e.g., I bet that ribosomal protein genes appear as one cluster)? And if so, does the specific sequence state (and their associated observations) match the known biology on these regulatory strategies (e.g., SAGA-dependent promoters vs TFIID-dependent promoters)? I understand that some answers appear in the supplement (I did not read it). I believe that they should be made more pronounced in the main text.*

We added a short paragraph in section “The transcription cycle shows gene-specific variation” with additional references to the Supplementary Information to underline the biological significance of our findings. Indeed, cluster 14 and 38 are enriched in genes associated with ribosome biogenesis, translation and other house-keeping functions. More strikingly, we found the DNA binding motif of SFP1 - a regulator of ribosomal protein and

ribosome biogenesis genes - to be enriched in promoter state P/T1, which is a frequent promoter state of cluster 14 and 38 genes.

• *As far as I can tell, the authors do not discuss how they initialized the model (number of directional/undirectional states) or how many random initialization points where used. They discuss this in the context of the simulation, but it is unclear whether the same procedure was used on the genomic data. It seems that this requires non-trivial manual tuning to get good results (which is worrisome).*

As Referee #1 confirms, there is no reliable automatic way to set the number of states. For the yeast data, the number of directed and undirected states was set manually, after experimenting with different state numbers. Be aware that bdHMM is used as an exploratory tool in this context. As with all unsupervised discretization methods, the appropriate number of states depends on the amount of detail one wants to see.
Indeed, parameter initialization is important. We found that initialization by k-means works very well and generally converges to a higher likelihood than multiple random starts, in agreement with [Rabiner (1989), reference in the manuscript]. We therefore advise to use k-means for parameter initialization, which does not require manual tuning. To not introduce further biases towards the k-means initialization and allow the EM to explore solutions which are further from it, covariance matrices were initially set to the covariance of the whole data and transition and initial state probabilities were initialized uniform.
For the yeast data, the strand-specific expression data was first split into regions expressed on either the + or - strand and unexpressed regions. Directed states were initialized as a k-means clustering from the expressed regions while undirected states were initialized using k-means on the unexpressed regions.
In the absence of strand-specific data and without directionality annotation, it is non-trivial to determine whether a state is directed or not. In order to minimize the amount of manual intervention, we introduced the directionality score that can be used as a posterior criterion to merge twin states into one undirected state (see the bdHMM application to the human data). The merged model can then be used as an initialization to another round of bdHMM learning. We comment on this in section “Genomic state annotation results in a global, strand-specific transcription map” in the main text and added a section “Initialization of bdHMMs” in “Materials and Methods”. All pre- and post-processing steps are well documented in the STAN package vignette.

• *A useful initialization can be based on standard HMM learning. Learn a model, and then introduce directional states (i,j, k) to represent state j appearing after i and before k. This defines an obvious conjunction function -(i,j,k) = (k,i,j) and provides a nice initialization for the model. Of course, this initialization comes at the price of cubic number of states, but these can be easily trimmed down (removing rare triplets) or simplified by state merging.*

This is an interesting idea for a definition of directed states. However it will probably not be practical due to the large number of states that is introduced, even after merging. Additionally, the most likely triplets will be (i,i,i), (i, i, k), (k, i, i) or (i,k,i) do not give rise to meaningful directed states. Even worse, triplets of the kind (i,j,k) have an increased chance of being erroneous, since the Viterbi path annotation of the HMM should be smooth. Finally, it is unclear how to initialize the bdHMM emission distributions of a triple (i,j,k). The most obvious choice, the HMM emission distribution of the state j, would produce many states with initially identical emissions. We therefore like to stick to our “elimination” procedure based on

k-means clustering and the merging of directed states based on the directionality score, which works well in practice.

- *Regarding the initialization, in that simulation section, the authors initialize from a slightly perturbed uniform distribution. While this initialization escapes the saddle-point defined by the uniform distribution, it might not be enough to explore solutions that are further from it.*

In fact, we do not even perturb the uniform distribution (transitions and initial state probabilities are initialized exactly uniform). The initialization by a uniform distribution for the transition matrix and the initial state is uncritical (in particular, it does not define a saddle point of the likelihood function), for the following reason: In case of a uniform transition matrix, the forward and backward probabilities in the E-step of the EM algorithm entirely depend on the emission distributions. The transition probabilities are then updated in the M-step based on these forward and backward probabilities. This is equivalent to initializing the transition matrix with empirical transition frequencies obtained from a clustering. Nonetheless this is a fair concern. We provide now more information about the initialization procedure (section “Initialization of bdHMMs” in Materials and Methods). Moreover, the new simulations (see below) demonstrate the overall stability of the inference procedure.

- *I would appreciate simulations also from the model learned from the genome. That is, take the model learned (from yeast for example), generate data from it, and then try to learn from it. This simulation will give some intuition how hard it is to learn a model such as the one you learned, and will give you confidence estimate in the parameters (the procedure is essentially parametric jackknife).*

We have now carried out simulations from the model learned on the yeast data set. Parameters were recovered with high accuracy, confirming the validity and stability of our model and EM algorithm. We added a Figure (Supplementary Figure 9) showing the results of the simulations and refer to it in section “Genomic state annotation results in a global, strand-specific transcription map”. We are thankful for this suggestion, which improved the quality of the manuscript.

Technical comment:

- *The authors go into length in presentation of the EM convergence analysis and discussion of "approximate" version. I believe the following will simplify the presentation and prove the correctness of this approximation. First, note that while EM with constraints is generally hard, equality constraint are easy (\theta_i = \theta_j) since we can write the likelihood (or Q) in terms of reduced parameter set that do not contain redundancies. Second, we can reparameterize a bdHMM with the following parameters:\theta_{i,j} = a_{i,j} / \pi_i and the vector \pi. To see this, not that whenever a_{i,j} appears in the likelihood, simply replace with a_{i,j} * \pi_i. Given the constraints (2) & (3), we can formulate the constrains on \theta_{i,j}, (*) \theta_{i,j} = \theta_{-j,-i}, (**) \pi_i = \sum_j \theta_{i,j} It is also easy to verify that if \theta and \pi satisfy (*) & (**), then a and \pi satisfy (2) & (3).Moreover a is a stochastic if and only if (***) \sum_{i} \pi_i = 1. Thus we are left with three equality constraints (*), (3), and (4) and two sum constraints (**) and (***).*
From this point it is easy to check that \Q in terms of the new parameters is equal to ~Q of (eq 23) and that the maximum of this function is as given in eq (27).Thus, the authors are performing exact EM, where the internal optimization step has a unique solution (i.e., is convex).

The reparametrization of the optimization problem by replacing a_ij through \theta_ij is a brilliant idea, because it removes the generalized reversibility constraints. We assume that the reparametrization by \theta_ij = a_ij/pi_i is a typo, because it does not satisfy equation (*). We use \theta_ij = a_ij/pi_j instead (division by \pi_j instead of \pi_i). Unfortunately, the

solution procedure proposed by the reviewer does not lead to our approximate update formula, because a) the approximate update provably does not always return values that exactly satisfy the bdHMM constraints (just check numerically), and b) using \theta_ij = a_ij/pi_j , equation (**) does not hold (due to the swap of \pi_i and \pi_j). We still think that the suggested reparametrization could lead to an exact solution, however we were unable to find such in the short time available. We therefore kept our original solution.

2nd Editorial Decision 17 November 2014

Thank you again for submitting your work to Molecular Systems Biology. We have now heard back from the two referees who agreed to evaluate your manuscript. As you will see from the reports below, the referees are overall satisfied with the modifications made and they think that the study is now suitable for publication. Reviewer #3 has included a file (attached below) related to the technical issue raised in his/her review of the initial version of the manuscript. As such, we would ask you to include a comment on this point in a revision of the manuscript.

Thank you for submitting this paper to Molecular Systems Biology.

Yours sincerely,

Reviewer #1:

The authors have satisfactorily addressed my comments.

Reviewer #3:

The attached note, which I believe resolves their technical issue, can be forwarded to the authors. I apologize for the typo in my review that lead them to a dead end.

2nd Revision - authors' response 19 November 2014

(see next page)

Dear Mrs. Polychronidou,

We are delighted by the acceptance of the paper. We thank Reviewer 3 again for his efforts in solving the EM algorithm for bdHMMs analytically. Following his suggestions in the previous review, we thoroughly investigated a change of variables in the objective function. It is encouraging to see that our ansatz is identical to the one proposed by reviewer 3 in his current comment. As detailed below, it turns out that the Lagrange multiplier approach does not lead to an exact analytical solution. Yet, it leads to another optimization strategy, whose computational complexity is identical to our algorithm. However, the strategy chosen in the manuscript has an intuitive motivation as a lower bound maximization, which the other approximation is lacking. Therefore, we decided to keep our solution. Since we would highly appreciate Reveiwer 3's further input on bdHMM learning, we kindly ask you to encourage Reviewer 3 to reveal his identity to us.

Additionally, the revised submission includes a .zip file containing the R package STAN, together with its vignette and user manual. We changed the manuscript title to "Annotation of genomics data using bidirectional hidden Markov models unveils variations in Pol II transcription cycle". We went through the abstract and synopsis, accepting essentially all suggested changes.

Thank you for your kind assistance in the review process,

Sincerely,

Achim Tresch

An alternative optimization strategy for the M-step in the Baum-Welch algorithm

The original target function Q is

$$Q\left(\theta;\,\theta^{old}\right) \quad = \quad \sum_{k,l\in\mathcal{K}}\sum_{t=1}^{T}\zeta_t\left(k,l\right)\log a_{kl} + \sum_{k}\gamma_0(k)\log\pi_k + const \qquad (1)$$

As suggested by reviewer 3, let $\rho_{ij} = \pi_i a_{ij}$. Then, ρ and π satisfy the conditions

$$\pi_i \geq 0\ , \quad i\in\mathcal{K} \qquad (2)$$

$$\sum_{i\in\mathcal{K}}\pi_i = 1 \qquad (3)$$

$$\rho_{ij} \geq 0\ , \quad i,j\in\mathcal{K} \qquad (4)$$

$$\sum_j \rho_{ij} = \sum_j \pi_i a_{ij} = \pi_i \tag{5}$$

$$\rho_{ij} = \rho_{\bar{j}\bar{i}} \tag{6}$$

It follows that A and π satisfy all conditions of a bdHMM if and only if ρ and π satisfy the five conditions above. We therefore We substitute a_{ij} by ρ_{ij}/π_i and introduce Lagrange multipliers μ_k, $k \in \mathcal{K}$, and λ, to account for constraints (5) and (3). This leads to the modified target function $\tilde{Q}$,

$$\begin{aligned}
\tilde{Q}(\rho, \pi; \theta^{old}) &= \sum_{k,l} \sum_{t=1}^{T} \zeta_t\,(k,l) \log \frac{\rho_{kl}}{\pi_k} + \sum_k \gamma_0(k) \log \pi_k \\
&\quad + \sum_k \mu_k \left(\pi_k - \sum_l \rho_{kl} \right) + \lambda \left(1 - \sum_k \pi_k \right) \\
&= \sum_{k,l} \sum_{t=1}^{T} \zeta_t\,(k,l) \log \rho_{kl} - \sum_k \sum_{t=1}^{T} \gamma_{t-1}(k) \log \pi_k \\
&\quad + \sum_k \gamma_0(k) \log \pi_k + \sum_k \mu_k \left(\pi_k - \sum_l \rho_{kl} \right) + \lambda \left(1 - \sum_k \pi_k \right) \\
&= \sum_{k,l} \sum_{t=1}^{T} \zeta_t\,(k,l) \log \rho_{kl} - \sum_k \sum_{t=1}^{T-1} \gamma_t(k) \log \pi_k \\
&\quad + \sum_k \mu_k \left(\pi_k - \sum_l \rho_{kl} \right) + \lambda \left(1 - \sum_k \pi_k \right) \\
&= \sum_{k,l} Z_{kl} \log \rho_{kl} - \sum_k G_k \log \pi_k \\
&\quad + \sum_k \mu_k \left(\pi_k - \sum_l \rho_{kl} \right) + \lambda \left(1 - \sum_k \pi_k \right)
\end{aligned} \tag{7}$$

with the constant terms $Z_{kl} = \sum_{t=1}^{T} \zeta_t\,(k,l)$ and $G_k = \sum_{t=1}^{T-1} \gamma_t(k)$. Taking into account that $\rho_{\bar{j}\bar{i}} = \rho_{ij}$ and $\pi_{\bar{i}} = \pi_i$, the partial derivatives of $\tilde{Q}$ with respect to π_i and ρ_{ij} become

$$\frac{\partial \tilde{Q}}{\partial \pi_i}(\theta; \theta^{old}) = \begin{cases} -\frac{G_i + G_{\bar{i}}}{\pi_i} + \mu_i + \mu_{\bar{i}} - 2\lambda & \text{if } i \neq \bar{i} \\ -\frac{G_i}{\pi_i} + \mu_i - \lambda & \text{if } i = \bar{i} \end{cases} \tag{8}$$

and

$$\frac{\partial \tilde{Q}}{\partial \rho_{ij}}(\theta; \theta^{old}) = \begin{cases} \frac{Z_{ij} + Z_{\bar{j}\bar{i}}}{\rho_{ij}} - \mu_i - \mu_{\bar{j}} & \text{if } i \neq \bar{j} \\ \frac{Z_{ij}}{\rho_{ij}} - \mu_i & \text{if } i = \bar{j} \end{cases} \tag{9}$$

For ease of notation, let $z_{ij} = Z_{ij} + Z_{\bar{j}\bar{i}}$ and $g_i = G_i + G_{\bar{i}}$. Setting the partial derivatives to zero, we obtain

$$\lambda = \frac{1}{2}\left(\mu_{\mathbf{i}} + \mu_{\bar{\mathbf{i}}} - \frac{g_i}{\pi_i}\right) \tag{10}$$

and

$$(\mu_i + \mu_{\bar{j}})\rho_{ij} = z_{ij} \tag{11}$$

(note that Equations (10) and (11) also hold if $i = \bar{i}$ and if $i = \bar{j}$). Summing over i and j in (11) yields

$$\begin{aligned}
\sum_i \mu_i \sum_j \rho_{ij} + \sum_j \mu_{\bar{j}} \sum_i \rho_{\bar{j}\bar{i}} &= \sum_{i,j} z_{ij} \\
\sum_i \mu_i \pi_i + \sum_j \mu_{\bar{j}} \pi_{\bar{j}} &= 2T \\
\sum_k \mu_k \pi_k &= T \qquad (12)
\end{aligned}$$

Moreover,

$$\begin{aligned}
\sum_i g_i &= \sum_i (G_i + G_{\bar{i}}) \\
&= \sum_i \sum_{t=1}^{T-1} (\gamma_t(i) + \gamma_t(\bar{i})) = \sum_{t=1}^{T-1} 2 \\
&= 2(T-1) \qquad (13)
\end{aligned}$$

Multiplying (10) by π_i and summing over i yields

$$\begin{aligned}
\lambda &= \sum_i \lambda \pi_i \\
&= \sum_i \frac{1}{2}\left(\mu_i \pi_i + \mu_{\bar{i}} \pi_i - g_i\right) \\
&= -\frac{1}{2}\sum_i g_i + \frac{1}{2}\sum_i \mu_i \pi_i + \frac{1}{2}\sum_i \mu_{\bar{i}} \pi_{\bar{i}} \\
&\overset{(12,13)}{=} -\frac{1}{2} \cdot 2(T-1) + \frac{1}{2}T + \frac{1}{2}T \\
&= 1 \qquad (14)
\end{aligned}$$

Substituting $\lambda = 1$ back into (10) and rearranging terms, we get

$$\mu_i + \mu_{\bar{i}} = \frac{g_i}{\pi_i} + \lambda = \frac{g_i}{\pi_i} + 2 \tag{15}$$

Plug (15) into Equation (11) to obtain

$$\begin{aligned}
\frac{z_{ij}}{\rho_{ij}} = \mu_i + \mu_{\bar{j}} &= (\mu_i + \mu_{\bar{i}}) + (\mu_j + \mu_{\bar{j}}) - (\mu_j + \mu_{\bar{i}}) \\
&= \frac{g_k}{\pi_i} + 2 + \frac{g_j}{\pi_j} + 2 - \frac{z_{ji}}{\rho_{ji}} \qquad (16)
\end{aligned}$$

Use $\tau_{ij} = z_{ij}\rho_{ij}^{-1}$

$$\tau_{ij} + \tau_{ji} = \frac{g_i}{\pi_i} + \frac{g_j}{\pi_j} + 4 \tag{17}$$

for τ_{ij} and hence determine ρ_{ij}. We guess an approximate solution

$$\tau_{ij} = \frac{g_i + \gamma_0(i) + \gamma_T(\bar{i})}{\pi_i} \tag{18}$$

or

$$\rho_{ij} = \frac{\pi_i z_{ij}}{g_i + \gamma_0(i) + \gamma_T(\bar{i})} \tag{19}$$

Note, that by choosing this specific solution, we are able to guarantee that the updated parameters satisfy all constraints of a bdHMM (see below). However, we cannot guarantee that the updated parameter set will have a marginal likelihood larger or equal to the previous parameter set θ^{old}.

With this choice for ρ_{ij}, condition (5) is fulfilled:

$$\begin{aligned}
\sum_j z_{ij} &= \sum_j (Z_{ij} + Z_{\bar{j}\bar{i}}) = \sum_{t=1}^{T} \sum_j (\zeta_t(i,j) + \zeta_t(\bar{j},\bar{i})) \\
&= \sum_{t=1}^{T} (\gamma_t(i) + \gamma_t(\bar{i})) \\
&= \gamma_0(i) + \sum_{t=1}^{T-1} (\gamma_t(i) + \gamma_t(\bar{i})) + \gamma_T(\bar{i}) \\
&= \gamma_0(i) + G_i + G_{\bar{i}} + \gamma_T(\bar{i}) \\
&= g_i + \gamma_0(i) + \gamma_T(\bar{i}) \qquad (20)
\end{aligned}$$

And consequently,

$$\sum_j \rho_{ij} = \frac{\pi_i}{g_i + \gamma_0(i) + \gamma_T(\bar{i})} \sum z_{ij} = \pi_i \tag{21}$$

The solution ρ, π therefore satisfies all conditions of a bdHMM. In case of convergence (so far we have not seen an exception, but we have not checked extensively), $\gamma_0(i) = \pi_i = \pi_{\bar{i}} = \gamma_T(\bar{i})$, and the approximation in (18) becomes an exact solution. We have then found a local maximum θ.
